# Tuning the Surface: Screen-Printed Flexible Porous Nanocomposite Electrodes with Programmable Electrochemical Performances for Wearable Platforms

**DOI:** 10.1021/acssensors.4c03519

**Published:** 2025-02-28

**Authors:** Adisak Pokprasert, Natcha Rasitanon, Irlesta Rahma Lani, Itthipon Jeerapan

**Affiliations:** † Center of Excellence for Trace Analysis and Biosensor, Prince of Songkla University, Hat Yai, Songkhla 90110, Thailand; ‡ Division of Physical Science, Faculty of Science, Prince of Songkla University, Hat Yai, Songkhla 90110, Thailand; § Center of Excellence for Innovation in Chemistry, Faculty of Science, Prince of Songkla University, Hat Yai, Songkhla 90110, Thailand; ∥ The ijE Electrochemistry for All Laboratory, Hat Yai, Songkhla 90110, Thailand

**Keywords:** surface engineering, surface electrochemistry, selective interaction, flexible electrodes, screen printing, porous nanocomposites, electrochemical sensors

## Abstract

Flexible electrodes fabricated through cost-effective thick-film strategies are important for developing electrochemical devices, such as sensors. Properly engineered nanocomposite electrodes can enhance the electrochemically active surface area, facilitate mass and charge transport, and allow for tailored surface chemistry and structure. Although great efforts have been devoted to developing porous nanocomposite electrodes, a facile method to achieve screen-printed porous nanocomposite electrodes in the form of flexible electrodes with tunable electrochemical performance has been overlooked. This article introduces a strategy for fabricating flexible porous electrodes using screen printing and electrochemical surface treatments, resulting in enhanced surface chemistry and electrochemical properties. By applying selective etching and anodization, the electrode’s surface area increases by 214% compared to a nontreated electrode, enabling programmable sensitivity to specific molecules. The engineered electrode improves the hydroquinone-to-salicylic acid detection ratio from less than 1 to over 10, allowing selective detection of neutral and positively charged molecules while rendering the electrode inactive for negatively charged species. This flexible sensor can be integrated into a wearable glove for rapid analysis and has also been successfully implemented in a second-generation glucose biosensor. This approach holds significant potential for advancing surface electrochemistry, offering new possibilities for tailoring electrode surfaces for diverse analytical applications.

## Graphical abstract

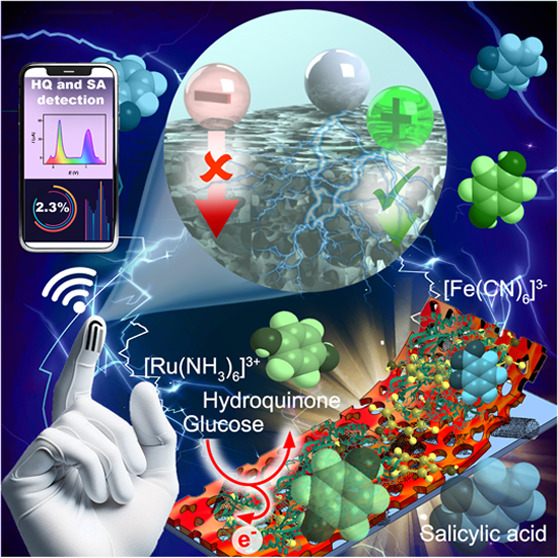

Surface engineering strategies have been developed to tailor desirable surface properties such as adhesion, wettability, corrosion resistance, and catalytic activity.^1,2^ Notably, engineering the electrochemical surfaces of nanocomposite electrodes is crucial for enhancing the performance of energy storage and conversion devices by governing essential parameters such as electrochemically active surface, charge–discharge behavior, energy density, and cycling stability.^3^ Additionally, surface engineering plays a significant role in improving electrochemical sensors by enhancing sensitivity, response time, limit of detection, and selectivity.^4^ Surface electrochemistry is particularly significant because the electrode surface determines how it interacts with electrolytes and desirable electroactive species, and controls electron transfer at the interfacial layer. For sensors, tuning sensitivity, selectivity, and detection limits which are influenced by surface electrochemistry can be achieved through the introduction of advanced materials or functional groups onto the surface.^5^ To further improve the performance of electrodes, in addition to integrating nanomaterials, careful research into engineering techniques is essential to exploit.

Developing efficient electrochemical sensors requires enhancing the sensitivity by designing porous electrodes with a large surface area to maximize electron transfer efficiency and strengthening the electroanalytical signal.^2,6^ Techniques such as chemical vapor deposition, dealloying, electrodeposition, and lithography, although effective, are impractical for large-scale production of flexible electrodes due to their complexity and cost.^7,8^ For instance, graphite/platinum cluster electrodes on a porous poly­(tetrafluoroethylene) substrate were fabricated using magnetron sputtering codeposition for sensors^9^ Another approach involved using vapor-phase dealloying to create three-dimensional nanoporous cobalt, which increased the surface area.^10^ A graphene electrode fabricated using CVD and liquid etching techniques was also developed to increase the porosity of the otherwise rigid electrode.^11^ However, an alternative technique should be simple, scalable, and economical. Printing technology meets these criteria by enabling the deposition of functional inks onto substrates. However, there have been limited efforts to create porous nanocomposite structures on flexible substrates using the screen-printing technique.

In addition to sensitivity, tuning selectivity is essential to ensure that the sensor primarily responds to the analyte while minimizing deviated readings caused by interfering agents. Nanocomposites can enhance sensor sensitivity but may also increase interference from unwanted species, posing a significant challenge in analytical chemistry when analyzing real samples. Traditional large and complex separation instruments are impractical for on-site analysis. Advanced electrochemical sensors offer a portable, cost-effective alternative; however, achieving both selectivity and sensitivity remains highly challenging and requires careful material engineering. Therefore, it is crucial to design surfaces that selectively transport and interact with desired species. Strategies should focus on enhancing the mass transfer of target analytes by engineering the electrode surface to attract oppositely charged species while repelling interfering species with the same charge. For example, surface coating with charged polymers (e.g., chitosan, poly­(lactide-*co*-glycolide), and Nafion) can selectively attract or repel charged species based on their electrostatic interactions.^12,13^ Electrodeposited overoxidized poly­(1,2-phenylenediamine) on a carbon fiber microprobe creates negatively charged surfaces to measure cationic dopamine while repelling anionic ascorbic acid.^14^ Similarly, vertically aligned silica mesochannels with positively charged ammonium groups and PEDOT/Nafion composite coatings enable selective detection of dopamine, enhancing permselectivity.^15^ Tuning selectivity requires a thorough investigation and systematic control of surface electrochemistry, as it dictates the behavior between the electrode and the species in the testing solution. This is particularly important in complex mixtures with multiple electroactive components, where these interactions can influence the detection of each compound. For example, in real-world applications, the simultaneous presence of hydroquinone (HQ) and salicylic acid (SA) in skincare products—commonly used for their skin-lightening properties—requires careful analysis, necessitating the tuning of the electrode sensor’s selectivity. The tuning and programming of selectivity by combining enhanced sensitivity and practical adaptability offers an approach to overcome limitations of traditional methods. The electrode’s ability to suppress interfering species while amplifying target analyte signals tackles real-world challenges in complex matrices, such as skincare products, where coexisting analytes often hinder accurate detection.

Flexibility is crucial for electrodes in modern applications such as soft electronics and robots, and wearable sensors. For example, wearable electrodes must be flexible to adapt to changes in shape as the wearer moves, ensuring stable signal acquisition. This flexibility also extends the device’s lifespan and effectiveness by withstanding mechanical distortion, offering significant advantages over rigid platforms.^16–18^ Furthermore, comparing the literature on electrochemical sensors employing diverse materials for analyte detection (outlined in Table S1) reveals that, to date, there are no reports on any facile strategies on fabricating the flexible, porous, surface-engineered nanocomposite electrodes with programmable surface properties including systematically programming desirable electrochemical properties and tuning selective interactions The unique features set our work apart from previous studies, which have overlooked strategies to effectively control sensitivity and selectivity on flexible electrodes, a critical gap that limits their practical applicability.

This article presents a strategy to systematically tune the electrochemical properties of flexible, porous nanocomposite electrodes prepared using the screen-printing technique combined with selective acid etching and electrochemical surface engineering. The porous electrode characteristics are identified through morphology observation, gas adsorption, and electrochemical studies, including analysis of the electrochemically active surface area. The aim is to explore insights into systematically programming desirable electrochemical surface behaviors by adjusting applied potentials during surface treatment for sensitive and selective detection of differently charged species, such as standard redox probes, HQ, and SA. Furthermore, the flexible, surface-engineered electrochemical sensor is printed on a wearable glove platform to demonstrate rapid on-demand screening of skincare samples. Another demonstration also employs porous nanocomposite electrodes for active mediator adsorption, aimed at developing second-generation biosensors. The implications are expected to extend across various domains, such as enabling selective analyte monitoring and advancing the performance of flexible electrode technologies.

## Results and Discussion

### Concept of Screen-Printed Porous Nanocomposite Electrodes and Its Applications

Carbon nanotubes (CNTs) were incorporated into the composite due to their high electrical conductivity and large surface area, both essential for enhancing electrode performance.^19^ The incorporation of CNTs overcomes the low electrical conductivity limitations of conventional screen-printing inks. Graphite was also incorporated to provide further electrical conductivity and stability to the electrode. Its layered structure facilitates good electron mobility, complementing the conductive properties of CNTs.^20^ Styrene-ethylene-butylene-styrene (SEBS) serves as a binding agent, imparting mechanical flexibility to the electrode and preventing the detachment of active conducting materials from the substrate.^21^ This flexibility is particularly vital for applications where the electrode needs to withstand deformation. Toluene was added to ensure homogeneous dispersion of the composite components and to maintain the desired viscosity of the modified ink, making it suitable for the screen-printing method. The dispersion underwent sonication and high-speed mixing to achieve a uniform and stable ink formulation, ensuring even distribution of all components. Finally, the CNT electrode was fabricated by screen-printing the electrode ink onto a flexible substrate. An unmodified screen-printing electrode was used to prepare a control electrode using the same procedure but with ink lacking CNTs and additional SEBS.

The CNT electrode was further utilized as the bottom underlying layer to construct the porous structure on top. Porosity is a key design feature in advanced electrode materials, as it directly enhances the electrochemically active surface area by introducing interconnected pores, facilitating ion transport, analyte diffusion, and electron transfer at the electrode surface. To introduce additional porosity into the printed materials, sodium hydrogen carbonate, acting as a porogen, was incorporated into the printable material matrix (Figure 1A). This was achieved by mixing the porogen with conductive ink and CNTs. Sodium hydrogen carbonate was ground before adding to ensure consistent particle sizes, ensuring uniformity of the porosity in the electrode. Subsequently, these porogens were selectively etched out, leaving a network of pores within the printed material. This etching involves the thermodynamically favorable reaction of sodium hydrogen carbonate with an acid (the reaction constant^22^ of 1.25 × 10^6^ and the Gibbs free energy change (Δ*G*°) of −36.2 kJ), producing carbon dioxide bubbles that create a porous structure, increasing the electrochemically active surface area and facilitating mass transfer of electrolyte and desired species.

**Figure 1:**
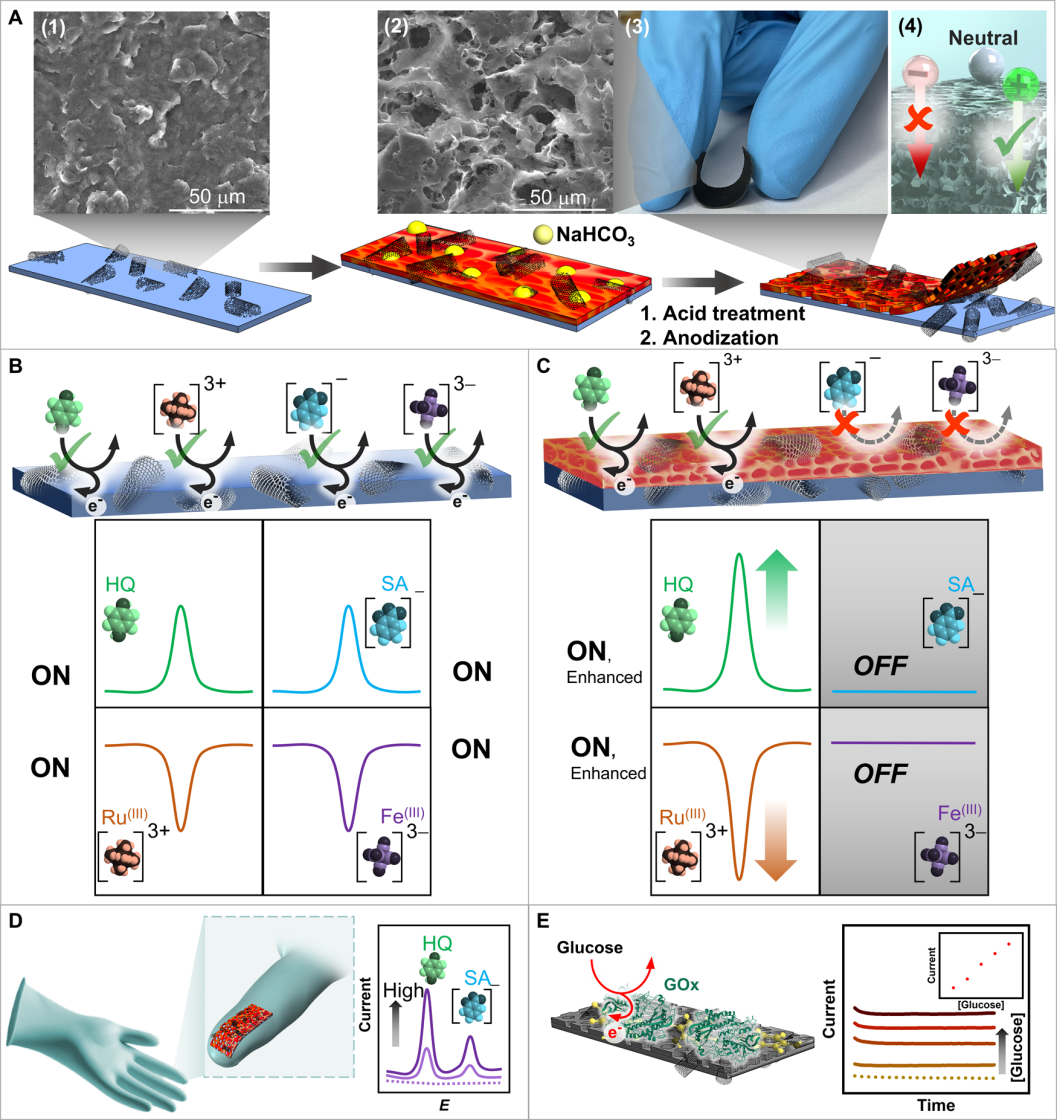
Concept of screen-printed porous nanocomposite electrode and its applications. (A) Fabrication of screen-printed porous nanocomposite electrode prepared by acid treatment and anodization, including SEM images of (1) CNT electrode (2) PCNT_2.0_ electrode. (3) The photo of PCNT_2.0_ electrode on a flexible substrate and (4) the illustration of selective effect toward positively charged, neutral, and negatively charged analytes on the surface of the PCNT_2.0_ electrode. (B–C) Conceptual illustration comparing. (B) the CNT electrode surface and (C) the PCNT_2.0_ electrode surface. The illustrations show the oxidation reactions of HQ and SA, and the reduction reactions of hexaammineruthenium­(III) ion ([Ru­(NH_3_)_6_]^3+^) and hexacyanoferrate­(III) ion ([Fe­(CN)_6_]^3–^) on their respective surfaces, with conceptual SWV responses. The PCNT_2.0_ electrode demonstrates enhanced sensitivity and selective rejection in the SWV responses. (D) Illustration of a flexible PCNT_2.0_ electrode printed on a wearable glove for on-finger electrochemical detection, revealing analytical responses to a sample containing HQ and SA. (E) Flexible porous glucose biosensor relying on adsorbed HQ redox mediator and surface-coated glucose oxidase (GOx) enzyme biomolecules, enabling amperometric glucose detection.

To evaluate the effectiveness of the selective etching process and observe its effects on the electrode surface, morphological changes were examined using SEM. The resulting printed material of the CNT electrode shows a compact morphology with a rough surface compared to the smoother, unmodified electrode, as shown in SEM images in Figure 1A­(1) and Figure S1. The CNT electrode refers to a flexible, screen-printed electrode modified with CNTs (see Supporting Experimental Procedures). In contrast, the PCNT_2.0_ electrode exhibits a porous, rough morphology (Figure 1A­(2)). This PCNT_2.0_ electrode (with the ‘2.0′ subscript) is a flexible, porous CNT-based nanocomposite electrode that has undergone electrochemical treatment across a potential range of 1.75 to 2.25 V, with a midpoint of 2.0 V (see Supporting Experimental Procedures).

This comparison highlights the successful introduction of porosity on the printed surface of the electrode. Furthermore, Figure 1A­(3) shows a photograph of a porous electrode printed on a thin plastic sheet, demonstrating its flexibility during bending.

While the addition of CNTs enhances the electrical conductivity and surface area of the electrodes, inherent limitations such as poor electron transfer of untreated surfaces and insufficient surface functionalization to interact with desired chemical species (e.g., analytes) necessitate additional surface modifications to facilitate electron transfer. Various techniques for surface modification, including polishing,^23^ mechanical approaches,^24^ ozone treatment,^25^ and plasma treatment,^26^ have been demonstrated. However, polishing and mechanical methods can be labor-intensive, impractical for small and flexible electrodes, and challenging for achieving the uniformity needed for consistent electrochemical performance. Strong acid treatments can damage the substrate, particularly soft materials used in flexible electrodes. Ozone and plasma treatments may also be harsh, potentially compromising the integrity of both the electrode material and the supporting flexible substrate. Therefore, in this work, we leveraged anodization, mildly performed by applying oxidation potentials in a Na_2_CO_3_ solution, to introduce oxygenated species on the carbon-based surface,^27^ improving sensitivity for positively charged and neutral species while repelling negatively charged ones (Figure 1A­(4)). Moreover, anodization enhances the adsorption of targets (positively charged and neutral species) and facilitates electron transfer during electrochemical reactions. Our approach integrates the use of porous nanocomposite electrodes with anodization to tune the desirable surface of the electrode, leading to altered interaction with target analytes and tuning performance in electrochemical sensing.

Figure 1B,C provide a simplified illustration depicting the contrasting electrochemical performances of CNT and PCNT_2.0_ electrodes. The “ON” state denotes the electrode’s active mode, capable of sensitively detecting target analytes, while the “OFF” state signifies its inactive mode, resulting in a low electrochemical response. Our strategies to tune the surface electrochemistry demonstrate that the PCNT_2.0_ electrode enhances the detection of neutral analytes (e.g., HQ) and positively charged analytes (e.g., Ru­(III) complex ion), while repelling negatively charged species (e.g., Fe­(III) complex ion and the negative form of SA).

The OFF state is a feature of the PCNT_2.0_ electrode, designed to minimize interference by suppressing electrochemical signals from nontarget species (such as SA). This selective modulation is achieved through advanced surface engineering techniques (Figure 1) By minimizing cross-talk and interference from undesired electroactive species, the OFF state enhances the system’s selectivity.

In electrochemical sensors, the selectivity and sensitivity of electrodes can be improved through surface modifications. For instance, surface-activated porous carbon electrodes have been proposed for the sensitive detection of dopamine in the presence of interfering species, such as ascorbic acid and uric acid.^28^ Additionally, electrode surfaces can be modified to enhance the selective detection of positively charged dopamine and negatively charged ascorbic acid mixed in biological fluids, as well as heavy metal ion pollutants (e.g., Pb^2+^, Hg^2+^, and Ag^+^).^14,15,29,30^ The heavy metal detection with enhanced electrode selectivity and sensitivity has been carried out by using functionalized silica films with thiol groups to create negatively charged surfaces (thiolate ion) that attract positively charged metal ions (Hg^2+^). These surface modifications provide selective binding to target analytes.

Developing effective materials capable of detecting multiple analytes in a single sample is crucial for simplifying the analytical process in complex matrices. A practical example is the simultaneous presence of HQ and SA in skincare products, where misuse can lead to significant health risks. HQ, which inhibits melanin production, can cause severe side effects, including dermatitis, inflammation, nausea, kidney damage, cancer, and an increased risk of leukemia.^31,32^ Despite bans by the European Union (EU),^33^ the illegal use of HQ continues in skin-lightening products. Additionally, the Scientific Committee on Consumer Safety (SCCS) of the European Commission has restricted the concentration of SA in skincare products to 0.5–3%.^34^ High concentrations of SA can also cause adverse effects such as redness, burning, pigmentation issues, and salicylate intoxication. These health concerns necessitate careful regulation and monitoring to ensure safety. The simultaneous dual-detection of HQ and SA offers it advantageous providing a practical solution particularly in skincare products where they often coexist, by simplifying the analytical workflow and reducing reliance on multiple instruments and complex procedures.

Additionally, HQ and SA are not only widely used in skincare products but also involved in diverse fields, including pharmaceuticals,^35,36^ environmental monitoring,^37^ and food control.^38,39^ Their relevance extends far beyond skincare analysis, emphasizing the critical need for effective monitoring to support broader applications across these areas.

Although techniques such as high-performance liquid chromatography (HPLC) and gas chromatography–mass spectrometry (GC-MS) are considered the gold standard, they have limitations, including complicated sample preparation, high costs, complex setups, the need for expert handling, time consumption, and impracticality for on-site or portable analysis.^40,41^ Similarly, methods, e.g., UV–vis spectroscopy and fluorescence-based probes, while effective, often lack portability and on-site practicality.^42–45^ These drawbacks make traditional techniques less suitable for applications requiring rapid, cost-effective, and user-friendly detection. Therefore, alternative methods offering user-friendly interfaces, rapid response, and sensitive and selective detection are needed. Electrochemical sensors meet these criteria, providing high sensitivity and selectivity by targeting specific electrochemical reactions of HQ and SA, with the added benefits of rapid response, on-demand practicality, and cost-effectiveness.^46,47^

In this work, we used neutral HQ and negatively charged SA as model analytes to investigate the selective detection capabilities of our flexible, porous electrodes, even though electrochemical sensors for detecting HQ or SA individually have been previously reported. The unique focus of our study aims to combine the flexibility of porous nanocomposite electrodes with electrochemical programmability, enabling fine-tuned selectivity toward the chosen analytes through electrochemical surface engineering on the screen-printed flexible electrodes.

We further demonstrate the application of our flexible PCNT_2.0_ electrochemical sensor by embedding it on a wearable glove to show its practicality in rapid and effective on-demand scenarios, as illustrated in Figure 1D. The skincare sample containing HQ and SA can be quickly analyzed by observing the electrooxidation peak signals. In addition to HQ and SA electroanalysis, the enhanced sensitivity of the flexible PCNT_2.0_ electrode toward the HQ response also contributes to advancements in second-generation glucose biosensors utilizing redox mediator and enzyme. Improving the immobilization of HQ mediators on the PCNT_2.0_ electrode enhances the performance of glucose enzymatic biosensors, as depicted in Figure 1E.

### Characteristics of Screen-Printed Flexible Electrodes

#### Electrochemical and Surface Characterizations of Screen-Printed Electrodes

We investigated the electrochemical behavior of printed electrodes—an unmodified electrode, a CNT electrode, and a PCNT_2.0_ electrode—by analyzing their electrochemical impedance spectroscopy (EIS). This study aimed to understand the charge-transfer resistance and the elements of the modeled circuit that characterize the electrodes’ electrochemical properties. An alternating current with a small potential perturbation of 10 mV was applied to record the EIS in a solution containing the Ru­(NH_3_)_6_Cl_3_ probe. It has to be noted that a typical potential perturbation of 10 mV was used in EIS measurements to maintain the system within its linear response range, ensuring accurate assessment of intrinsic interfacial properties such as charge transfer resistance without significantly perturbing or causing damage to the electrode material. The Nyquist plots shown in Figure 2A represent the electrochemical behavior of the unmodified electrode, CNT electrode, and PCNT_2.0_ electrode including equivalent circuit model as shown in Figure S2 comprising of the following elements: R_e_ (electrolyte resistance), *R*_ct_ (charge transfer resistance), CPE_dl_ (constant phase element for the double layer), and W_d_ (Warburg impedance) to account for ion diffusion. The summarized fitted parameters was provided Table S2. The PCNT_2.0_ electrodes have a lower charge transfer resistance (*R*_ct_) of 49.4 Ω, compared to the unmodified and CNT electrodes, which exhibit *R*_ct_ values of 124 and 96.4 Ω, respectively. Additionally, PCNT_2.0_ exhibited the lowest Bode modulus (approximately 76–85 Ω) across the frequency range of 10^3^ to 10^5^ Hz, outperforming both the CNT electrode (139–251 Ω) and the unmodified electrode (170–181 Ω), as illustrated in Figure S3. The lowest Bode modulus observed in the PCNT_2.0_ electrode indicates reduced surface resistance and enhanced electron transfer, particularly at low frequencies, highlighting its enhanced electrochemical performance.

**Figure 2:**
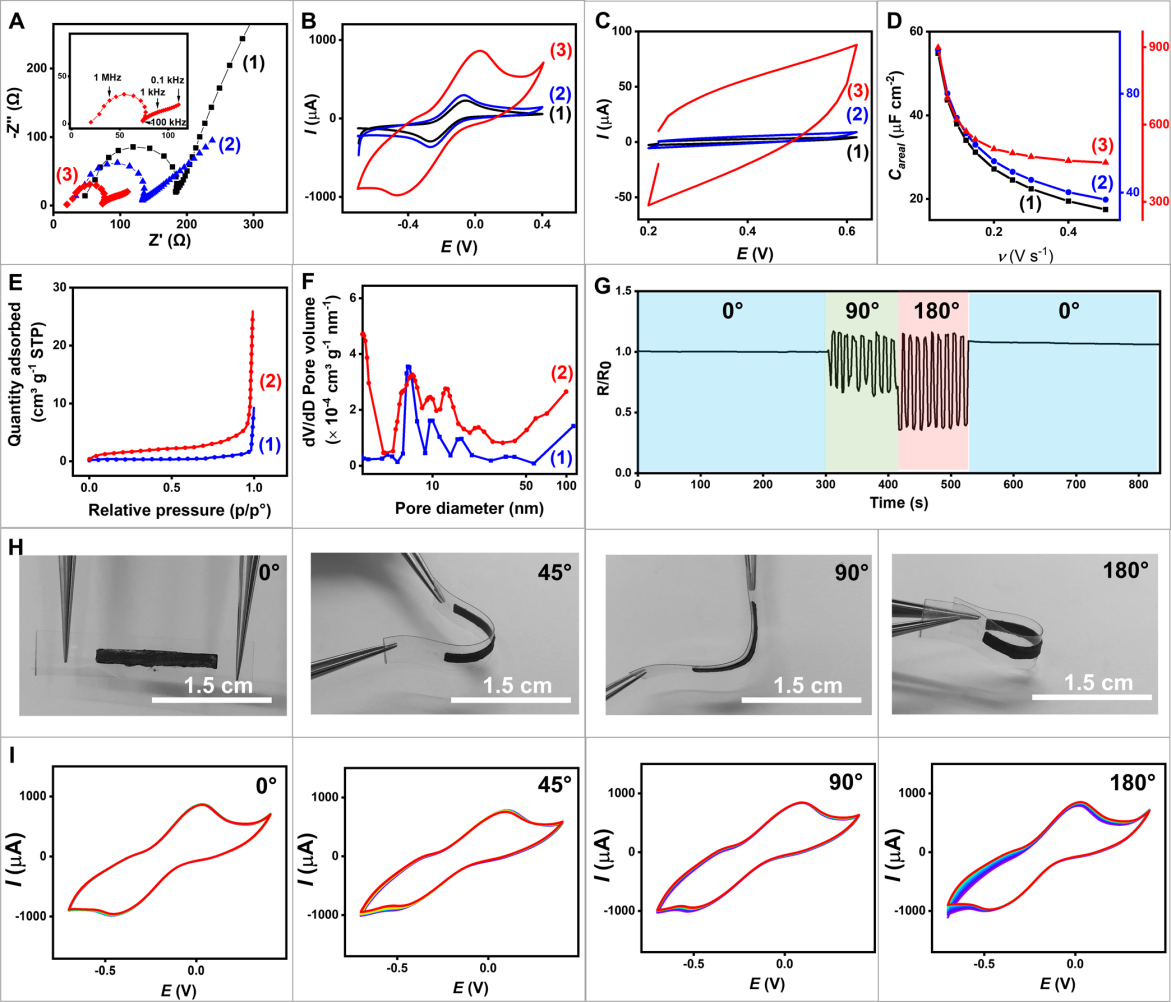
Characterization of Electrochemical Properties, Surface Analysis, and Mechanical Flexibility of Screen-Printed Electrodes. (A) Nyquist plots of (1) unmodified electrode, (2) CNT electrode, and (3) PCNT_2.0_ electrode in 0.1 M KCl containing 10 mM [Ru­(NH_3_)_6_]­Cl_3_. The inset shows zoomed-in EIS plot of the PCNT_2.0_ electrode. (B) Cyclic voltammograms (CVs) of (1) unmodified electrode, (2) CNT electrode, and (3) PCNT_2.0_ electrode in 0.1 M KCl containing 10 mM [Ru­(NH_3_)_6_]­Cl_3_ at a scan rate of 100 mV s^–1^. (C) CVs of (1) unmodified electrode, (2) CNT electrode, and (3) PCNT_2.0_ electrode at a scan rate of 500 mV s^–1^. (D) Capacitance of (1) unmodified electrode, (2) CNT electrode, and (3) PCNT_2.0_ electrode plotted at various scan rates (50 to 500 mV s^–1^). (E) Relationship of N_2_ adsorption isotherms versus relative pressure (p/p°) for (1) CNT electrode and (2) the top layer of the PCNT_2.0_ electrode. (F) Pore size distribution of (1) CNT electrode and (2) the top layer material of the PCNT2.0 electrode. (G) Electrical resistance of the PCNT_2.0_ electrode under repeated mechanical deformations. Measurements were taken at the relaxed state (0°), followed by repeated bending at 90 and 180°, and returning to 0°. (H) Photographs showing mechanical resiliency of the PCNT_2.0_ electrode by bending at 0, 45, 90, and 180°. (I) CVs recorded after 100 repeated bending cycles at angles of (1) 0°, (2) 45°, (3) 90°, and (4) 180° in a 0.1 M KCl solution containing 10 mM [Ru­(NH_3_)_6_]­Cl_3_ at a scan rate of 100 mV s^–1^.

This reflects a low charge *R*_ct_. Although the presence of CNTs improves the conductivity of the CNT electrode, it still exhibits higher resistance than the PCNT_2.0_ electrode, primarily due to the absence of a porous structure. The low resistance observed in PCNT_2.0_ implies its high effectiveness for electron transfer. To further investigate the electrochemical behavior of printed electrodes, we employed cyclic voltammetry (CV) in a redox standard solution (Ru­(NH_3_)_6_Cl_3_) to analyze peak positions (*E*_pa_ and *E*_pc_), peak currents (*I*_pa_ and *I*_pc_), and reversible behavior across three types of electrodes: unmodified, CNT, and PCNT_2.0_ (as depicted in Figure 2B). The CV data offers insights into the electron transfer properties, with peak current serving as an indicator of electrode surface area. The potential gap between anodic and cathodic peaks (Δ*E*_p_) insights into electron transfer efficiency and the active surface area. A smaller peak separation potential and increased peak currents indicate favorable electron transfer and a larger active surface area. The unmodified electrode shows small baseline-subtracted *I*_pa_ and *I*_pc_ of 223–290 μA, with a Δ*E*_p_ of 0.207 V. The CNT electrode demonstrates improvements, with larger *I*_pa_ and *I*_pc_ of 255–306 μA, and a smaller Δ*E*_p_ of 0.196 V. Remarkably, the PCNT_2.0_ electrode exhibits significantly enhanced anodic and cathodic peak currents, approximately 270% higher than the plain electrode (*I*_pa_ and *I*_pc_ of 731 and 806 μA). The results suggest quasi-reversible behavior for all electrodes, as indicated by the ratio between *I*_pa_ and *I*_pc_, which approaches 1. Furthermore, the Randles-Sevcik relationship can be applied to compare the electrochemically active surface area, which is proportional to the peak currents (Supporting Note S1). The PCNT_2.0_ electrode demonstrates the highest electrochemically active surface area of 0.29 cm^2^ compared to other electrodes (0.09 cm^2^ for the unmodified electrode and 0.10 cm^2^ for the CNT electrode).

We also examined the surface capacitive behavior to understand the active surface area of printed carbon-based electrodes in 0.1 M PBS (pH 7.0). Figure 2C shows the CV of the PCNT_2.0_ electrode compared to the unmodified and CNT electrodes. The CV results indicate that the PCNT_2.0_ electrode outperformed the others in terms of current response (Figures 2C and S4A–C) and apparent capacitance (*C*_app_), estimated based on Supporting eq S2 (Figure S4D–F). The significant increase in current response is attributed to the porous structure of the PCNT_2.0_ electrode, which enhances the surface area and facilitates greater ion accessibility, providing more active sites.

Additionally, Figure 2D presents the areal capacitance (*C*_areal_) of the three electrodes (details and calculations provided in Supporting Note S3 and eq S3). The PCNT_2.0_ electrode exhibits the highest *C*_areal_ of 898 μF cm^–2^ at a scan rate of 50 mV s^–1^ and maintains good capacitance retention, with a decrease of 50% at a fast scan rate of 500 mV s^–1^. In contrast, the unmodified and CNT electrodes show larger capacitance decays of 68 and 62%, respectively. The smaller decay at higher scan rates for PCNT_2.0_ suggests its stability due to enhanced ion accessibility provided by its highly porous surface structure. The large surface area and interconnected pores ensure facile ion transport and reduced resistance, underscoring the superior performance of the PCNT_2.0_ electrode.

Characterization of the surface area is crucial for optimizing the fabrication of porous electrodes. The surface area of the electrodes was evaluated using BET analysis. The adsorption isotherms (Figure 2E) indicate that the top layer of the PCNT_2.0_ electrode adsorbs a higher quantity of gas across all relative pressure ranges compared to the CNT electrode. Such an increased adsorption capacity of the PCNT_2.0_ electrode confirms its enhanced surface area. Moreover, the adsorption behavior of both the CNT electrode and the top layer of the PCNT_2.0_ electrode was correlated with density functional theory (DFT) model. The combination of BET and DFT analyses provides detailed insights into the surface and pore properties of materials, facilitating the calculation of pore size distribution and surface area. The BET surface area measurements reveal that the top layer of the PCNT_2.0_ electrode has a surface area of 5.7681 m^2^ g^–1^, which is larger than that of the CNT electrode by 213.9% (1.8373 m^2^ g^–1^). This enhanced surface area of PCNT_2.0_ correlates with its higher pore volume and adsorption capacity, as illustrated in the cumulative pore volume plot (Figure S5). The pore size distribution observed in Figure 2F, ranging from 3 to 120 nm, shows that the top layer of the PCNT_2.0_ electrode exhibits a pore diameter pattern similar to that of the CNT electrode, except that the CNT electrode lacks pores of 13 and 22 nm. The additional mesoporous formation in the PCNT_2.0_ electrode could be due to gas bubbles generated during the selective etching of NaHCO_3_ and anodization.

#### Mechanical Flexibility and Electrochemical Stability of Printed Electrodes for Enduring Electrochemical and Electrical Properties

To evaluate the mechanical resilience of the PCNT_2.0_ electrode, crucial for its practical applicability expected to undergo mechanical strain and movement, we conducted two sets of experiments. First, we investigated the change in resistance of the PCNT_2.0_ electrode during repetitive bending cycles at 0, 90, 180°, and returning to 0° (as shown in Figure 2G). Additionally, we further assessed its mechanical resilience by inspecting the electrode and recording CV data at varying bending angles: 0, 45, 90, and 180° (as depicted in Figure 2H,I). Figure 2G illustrates the change in relative resistance (R/R_0_) of the PCNT_2.0_ electrode over multiple bending cycles at different angles. During carrying out this experiment, the PCNT_2.0_ electrode was bent repeatedly from 0° to 90° to 180° and then back to 0° at a rate of 12 s per cycle. When the electrode was bent from 0 to 90°, the R/R_0_ value showed slight fluctuations as the electrode was distorted and then returned to its relaxed state. As the bending angle reached 180°, the resistance fluctuations became more pronounced. These fluctuations in resistance during bending are due to changes in the microscopic distance between conductive particles (MWCNTs and graphite).^48^ Remarkably, no major damage was observed, indicating mechanical durability. Notably, the graph demonstrates a consistent response to distortion, with the resistance consistently returning to its original value after each bending cycle. This recovery indicates the electrode’s ability to withstand repetitive mechanical stress without compromising electrical conductivity. Figure 2H illustrates the appearance of the PCNT_2.0_ electrode under different bending angles, ranging from 0° (no bending) to 180°. The electrochemical data in Figure 2I further support this observation, as the electrochemical response of the PCNT_2.0_ electrode remains consistent across varying bending angles. Despite multiple iterations of applied mechanical stress, minimal changes in peak positions and current intensities were detected, showing the electrode’s ability to maintain its electrochemical properties. This is advantageous for robust applications in scenarios involving movement and distortion.

We explored the effects of electrode surface modification through anodization by assessing water contact angles. The anodization process was conducted within an applied range from 1.75 to 2.25 V. Our investigation revealed that the CNT electrode without anodization (Figure S6A(1)) displayed a higher contact angle (104°) compared to the CNT_2.0_ electrode (75°) (Figure S6A(2)), indicating enhanced hydrophilicity. Note that the additional ‘2.0′ subscript in the electrode’s name indicates that the CNT electrode underwent electrochemical treatment at a midpoint potential of 2.0 V (within a range of 1.75 to 2.25 V). Similarly, the PCNT electrode exhibited a comparable trend, albeit with a less pronounced change in water contact angle when comparing the pristine PCNT with the PCNT_2.0_ electrode (with contact angles of 50 and 37° respectively, as shown in Figure S6B(1) and B(2)). The slight variation in contact angle for PCNT, compared to CNT, is attributed to the pre-existing surface treatment with acid. This decrease in water contact angle highlights the further enhanced hydrophilicity after anodization, suggesting that anodization introduces functional groups or structural changes that improve hydrophilicity. This observation is consistent with the expected effect of anodization on altering surface chemistry, by introducing oxygen-containing groups.^27,49,50^ The observed changes in surface wettability highlight the significant role of anodization in modifying the surface characteristics of electrodes, which is crucial for designing flexible printed electrodes with specific wettability.

The electrochemical stability of the PCNT_2.0_ electrode was also evaluated (Figure S7), showing over 85% current retention during repeated use and minimal performance degradation. Additionally, consistent redox activity with stable peak potentials and currents in CVs further highlights its robustness.

#### Electrochemical Behavior of HQ and SA on the Surface of Printed Electrodes

We investigated the electrocatalytic activity toward sensing HQ and SA using CV to understand their electrochemical behavior by examining the catalytic reactions at the heterogeneous interface between the electrode and the solution containing HQ or SA. Figure 3A presents the CV results illustrating the electrochemical behavior of the unmodified electrode, CNT_2.0_ electrode, and PCNT_2.0_ electrode toward HQ redox. The unmodified electrode exhibits a large peak separation (Δ*E*_p_) of 0.599 V. In contrast, the CNT_2.0_ electrode shows a Δ*E*_p_ of 0.120 V (*E*_pa_ at 0.180 V and *E*_pc_ at 0.060 V), indicating improved electron transfer kinetics. Notably, the PCNT_2.0_ electrode exhibits the strongest redox response with a small Δ*E*_p_ of 0.080 V (*E*_pa_ at 0.160 V and *E*_pc_ at 0.080 V). The decreased Δ*E*_p_ and enhanced peak current indicate the improved performance of the porous PCNT_2.0_ electrode in detecting HQ.

**Figure 3:**
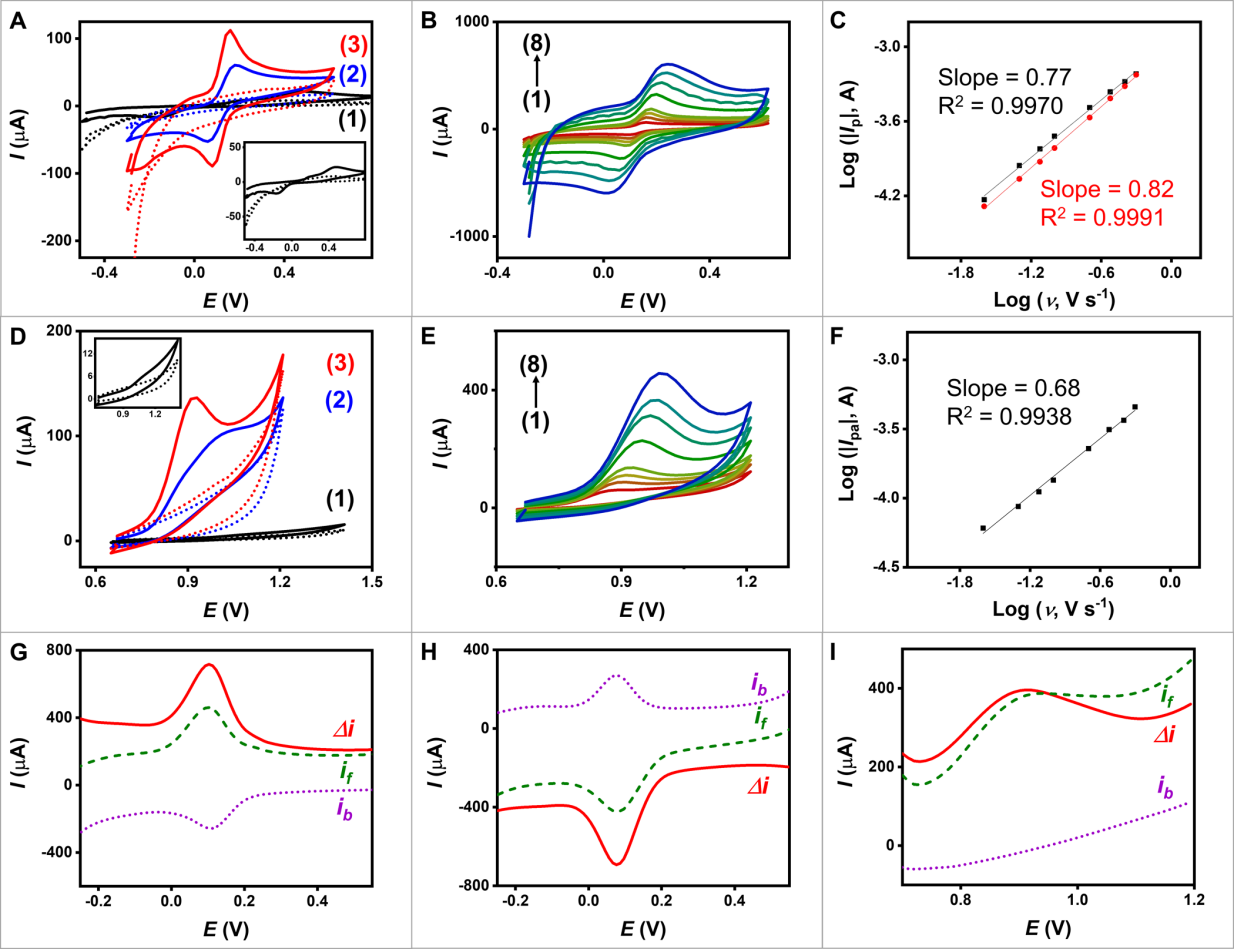
Electrochemical behavior of HQ and SA on the surface of printed electrodes. (A) CVs in 500 μM HQ for (1) unmodified electrode, (2) CNT_2.0_ electrode, and (3) PCNT_2.0_ electrode at a scan rate of 100 mV s^–1^. The inset shows zoomed-in CVs of the unmodified electrode. (B) CVs of the PCNT_2.0_ electrode in 500 μM HQ at various scan rates: (1–8) 25, 50, 75, 100, 200, 300, 400, and 500 mV s^–1^ (with potential step size of 20 mV). (C) Logarithmic plot of peak current versus scan rate for the PCNT_2.0_ electrode in 500 μM HQ. Black squares represent anodic peaks, and red dots represent cathodic peaks. (D) CVs in 2000 μM SA for (1) unmodified electrode, (2) CNT_2.0_ electrode, and (3) PCNT_2.0_ electrode at a scan rate of 100 mV s^–1^. The inset shows zoomed-in CVs of the unmodified electrode. (E) CVs of the PCNT_2.0_ electrode in 2000 μM SA at various scan rates: (1–8) 25, 50, 75, 100, 200, 300, 400, and 500 mV s^–1^ (with a potential step of 20 mV). (F) Logarithmic plot of peak current versus scan rate for the PCNT_2.0_ electrode in 2000 μM SA. Black squares represent anodic peaks. (G–H) SWVs for the detection of the PCNT_2.0_ electrode in 500 μM HQ in the oxidation. (G) and reduction (H) directions, showing response (Δ*i*), forward current (*i*_f_), and backward current (*i*_b_). (I) SWVs for the detection of the PCNT_2.0_ electrode in 2000 μM SA in the oxidation direction, showing response (Δ*i*), forward current (*i*_f_), and backward current (*i*_b_). Parameters for panels G-I: step potential 10 mV, amplitude 75 mV, and frequency 5 Hz.

At the molecular level, the reversible electrochemical redox reaction of HQ involves its oxidation to benzoquinone, losing two electrons and two protons and converting the hydroxyl groups into carbonyl groups. Its reduction back to HQ occurs by gaining two electrons and two protons.^51^ In our study, we also examined how the proton concentration in the supporting electrolyte solution influences the electrooxidation of HQ across a pH range of 4–10 (Figure S8A). As the pH of the supporting electrolyte increases, the anodic potentials for HQ oxidation shift toward more negative values. This observation indicates the involvement of protons in the oxidation process of HQ. Additionally, the peak potentials in different pH media exhibit a linear dependence on pH changes, with a slope of 68 mV, which is close to the theoretical Nernstian slope (Figure S8B). This observation suggests that the voltammetric oxidation of the analytes involves an equivalent number of protons and electrons.^52^

To further explore the electrochemical behavior of HQ on the modified electrode, we analyzed the number of protons and electrons transferred. The electron transfer kinetics of HQ on the PCNT_2.0_ electrode were investigated by varying the scan rates (Figure 3B), and the relationship between peak potential and the logarithm of the scan rate is shown in Figure S9. As the scan rate increased, the peak potential also shifted to higher values. Using Supporting eq S4, we calculated the number of electrons involved in HQ oxidation. The slope of the plot, defined as 2.303RT/αnF, yielded an αn value of 0.90 for HQ. For an irreversible electrode process, α is 0.5, leading to an estimated number of electrons transferred as two. This result indicates that the oxidation of HQ involves the transfer of two protons (2H^+^) and two electrons (2e^–^).

To further investigate the electrochemical behavior, we studied the effect of varying scan rates on the PCNT_2.0_ electrode (Figure 3B) compared to the unmodified electrode and CNT_2.0_ electrode (Figure S10A and S10C). The peak current increased with the scan rate for all electrodes.

The logarithmic scale plot of peak current versus scan rate reveals that the unmodified electrode and CNT_2.0_ electrode exhibit a slope of 0.5 (Figure S10B and D), indicating diffusion-controlled behavior. In contrast, the PCNT_2.0_ electrode exhibits a slope of around 0.7–0.8 (Figure 3C), suggesting mixed behavior between surface controlled and diffusion-controlled processes. The formal potentials, obtained from Supporting eq 5, show that the unmodified electrode has a formal potential of 0.15–0.16 V, while the CNT_2.0_ electrode and PCNT_2.0_ electrode exhibit around 0.13 V. The formal potential remains almost independent of the potential scan rate, suggesting facile charge transfer kinetics across porous electrode configurations.^53^ The electron transfer constant (*k* ^0^) of the electrodes toward HQ for the unmodified electrode, CNT_2.0_ electrode, and PCNT_2.0_ electrode were found to be 1.67 × 10^–3^ cm s^–1^, 6.66 × 10^–4^ cm s^–1^, and 1.36 × 10^–3^ cm s^–1^, respectively (refer to Supporting eqs 6 and 7, Supporting Note S6). These values align with those reported in other studies using carbon screen-printed electrodes,^54^ SWCNTs/reduced graphene oxide electrodes,^55^ and SWCNT buckypaper electrodes.^56^ Additionally, we calculated the catalytic rate constant (*K*_cat_) for the oxidation of HQ on the electrodes (referring to experimental data in Figure S11A–C and Supporting eq S8). The *K*_cat_ of the unmodified electrode is 1.37 × 10^5^ M^–1^ s^–1^, while the *K*_cat_ of CNT_2.0_ and PCNT_2.0_ increased by 2 orders of magnitude (2.90 × 10^7^ M^–1^ s^–1^) and 3 orders of magnitude (7.39 × 10^8^ M^–1^ s^–1^), respectively. The porous structure of the PCNT_2.0_ electrode significantly influences its electrochemical behavior, with implications for designing electrodes in sensor technology requiring efficient electron transfer.

CVs in SA solutions were also recorded (Figure 3D). The unmodified electrode exhibits an oxidation peak potential (*E*_pa_) at 1.110 V, whereas the CNT_2.0_ electrode shows an *E*_pa_ at 1.010 V, which is 0.100 V lower than the unmodified electrode. The PCNT_2.0_ electrode exhibits an *E*_pa_ of 0.930 V, which is 0.180 V lower than the unmodified electrode. These lower oxidation peak potentials observed for the CNT_2.0_ and PCNT_2.0_ electrodes correlate with an increase in oxidation current, indicating improved catalytic efficiency and a higher current response due to enhanced kinetics. The oxidation of SA involves the loss of two electrons and two protons from its hydroxyl and carboxyl groups, leading to the formation of reactive intermediates such as catechol and quinone derivatives.^57^ The electron transfer kinetics of SA on the electrode were analyzed at scan rates ranging from 25 to 500 mV s^–1^ (Figure 3E). As the scan rate increased, the peak potential shifted accordingly (Figure S12). Using eq S4, the number of electrons involved in the oxidation of SA was estimated to be close to 2, indicating that the oxidation of SA proceeds via a two-electron process. Moreover, we calculated the catalytic rate constant (*K*_cat_) toward SA oxidation for the unmodified electrode as 5.05 × 10^2^ M^–1^ s^–1^. The *K*_cat_ for the CNT_2.0_ and PCNT_2.0_ electrodes were found to be 7.27 × 10^3^ M^–1^ s^–1^ and 1.68 × 10^4^ M^–1^ s^–1^, respectively (Figure S13 and Supporting Note S6). The effect of varying scan rates in SA solution was studied using the unmodified electrode (Figure S14A), CNT_2.0_ electrode (Figure S14B), and PCNT_2.0_ electrode (Figure 3E). The logarithmic scale plot of peak current versus scan rate reveals that both the unmodified electrode and CNT_2.0_ electrode show a slope of 0.30 (Figure S14B and S14D). The PCNT_2.0_ electrode exhibits a slope of 0.68 (Figure 3F).

The analysis of the slope in the logarithmic plot of peak currents versus scan rates provides valuable insight into the mechanisms occurring at the electrode surfaces (Supporting Note S8 and eq S9). For the PCNT_2.0_ electrode, the slopes for HQ (0.77–0.82) (Figure 3C) and SA (0.68) (Figure 3F) are higher compared to the unmodified and CNT_2.0_ electrodes, which show slopes closer to 0.50 (Figures S10B, S10D, S13B, and S13D). This suggests a shift from a purely diffusion-controlled process to one more influenced by capacitive behavior. Despite this shift, the range of slopes observed for PCNT_2.0_ indicates that diffusion-controlled contributions are still present, though to a lesser extent. The higher slopes of PCNT_2.0_ reflect its enhanced surface interaction properties, due to its porous structure, which facilitates more efficient molecular movement at the interface. The porous structure of the PCNT_2.0_ electrode plays an important role in enhancing electrochemical performance by increasing the active surface area, facilitating efficient mass transport, and enabling selective analyte interactions.

Furthermore, the SWV response of the PCNT_2.0_ electrode was also studied. Figure 3G presents the SWV response showing the corresponding anodic peak of HQ; additionally, Figure 3H illustrates the SWV response in the reduction direction. These current responses in both oxidation and reduction directions indicate the redox reversibility of HQ. For SA, the oxidation peak is observed in Figure 3I while the absence of a reduction peak (Figure S15), indicating the nonreversibility of the electron transfer process of SA. This observation corresponds with the CVs in Figure 3D, further demonstrating the nonreversibility behavior of SA in redox reactions.

To further assess electrochemical kinetics, a Tafel plot was constructed using data from the rising portion of the current–voltage curve at a low scan rate. A linear relationship between the logarithm of the current and the potential was observed. The Tafel slope for the PCNT_2.0_ electrode was 16 V^–1^, compared to 7 V^–1^ for the CNT electrode and 3 V^–1^ for the unmodified electrode (Figure S16A–C), confirming the superior catalytic efficiency of the PCNT_2.0_ electrode in HQ oxidation due to its enhanced surface area. For SA, the PCNT_2.0_ electrode had a slope of 6 V^–1^, while the CNT_2.0_ and unmodified electrodes showed slopes of 7 and 3 V^–1^, respectively (Figure S17A–C). This highlights the PCNT_2.0_ electrode’s improved surface area and rejection capability toward SA.

We also conducted experiments to detect HQ and SA using a fast scan rate of 750 mV s^–1^. This rapid voltammetric scan took about 2 s to detect HQ and SA. A clear HQ signal was observed (Figure S18A). In case of SA, the signal was detectable, however, it was not as clear as HQ (Figure S18B).

Understanding the molecular states of HQ and SA at different pH levels is crucial for interpreting their electrochemical and adsorption behavior. HQ exhibits a neutral charge at pH 7.0 due to its p*K*_a_ of 9.9 and 11.6.^58^ Conversely, SA, with a p*K*_a_ of 2.97, is predominantly negatively charged at pH 7.0 due to its carboxylic group.^59^ Here, we used neutral HQ and negatively charged SA as models to investigate the selective detection capabilities of our flexible, porous electrodes. In addition to examining the individual electrochemical behaviors of HQ and SA (previously discussed), studying their mixed solution offers further insights into their interactions and detection capabilities. This investigation aims to demonstrate the advantages of our electrode design in detecting differently charged species within a mixed sample through surface tuning.

The electrochemical response of unmodified, CNT_2.0_, and PCNT_2.0_ electrodes toward a mixed solution of HQ and SA was evaluated using CV (Figure S19). The CNT_2.0_ electrode showed significant enhancements in *I*_p_ for HQ and *I*_p_ for SA by 108 and 257%, respectively, compared to the unmodified electrode (which has *I*_p_ for HQ of 19 μA and *I*_p_ for SA of 17 μA). The PCNT_2.0_ electrode exhibited even greater current peak increases of 198% for HQ, and 90% for *I*_p_ SA compared to the CNT_2.0_ electrode. These enhancements reflect improved electrocatalytic activity and the porous structure of the PCNT_2.0_ electrode, which facilitates an enhanced electrochemically active surface area.

Additionally, the significantly decreased Δ*E*_p_ values for the CNT_2.0_ and PCNT_2.0_ electrodes—improving by 83% (with Δ*E*_p_ of 0.112 V) and 86% (with Δ*E*_p_ of 0.090 V), respectively—compared to the unmodified electrode (with Δ*E*_p_ of 0.668 V) indicate favorable electron transfer and an enhanced electrochemically active surface area. The variations in peak positions and current peak intensities among the electrodes highlight the importance of surface tailoring in enhancing electrochemical performance, confirming a promising approach for advanced programmable electrochemical sensors. Note that the weaker and less defined signal observed for SA as compared to HQ is due to the anodization-induced surface functionalization of the electrode.

#### Engineering Selective Effects toward Different Electrochemically Active Species on Printed Electrodes

To explore strategies for engineering electrode surfaces and systematically tailoring the sensitivity of electrocatalysts for different active species, we designed experiments to assess the electrochemical performance of four different electrodes (Figure 4A). Each electrode was fabricated either to serve as a control (without the addition of CNT and anodization) or to explore the effects of material compositions and engineered surface structures. The unmodified electrode shown in Figure 4A­(1) served as our control for comparison with the modified electrodes. The CNT electrode was fabricated by printing engineered ink containing CNTs onto a flexible substrate, aiming to study the impact of CNT addition on enhancing electrochemical sensitivity (Figure 4A­(2)). To further enhance performance, this pristine nonporous CNT electrode then underwent anodization within a potential window of 1.75 to 2.25 V (passing the midpoint of 2.0 V) to yield the CNT_2.0_ electrode (Figure 4A­(3)). Finally, as illustrated in Figure 4A­(4), the PCNT_2.0_ electrode was designed to combine a porous structure with surface modifications achieved through acid treatment and anodization.

**Figure 4:**
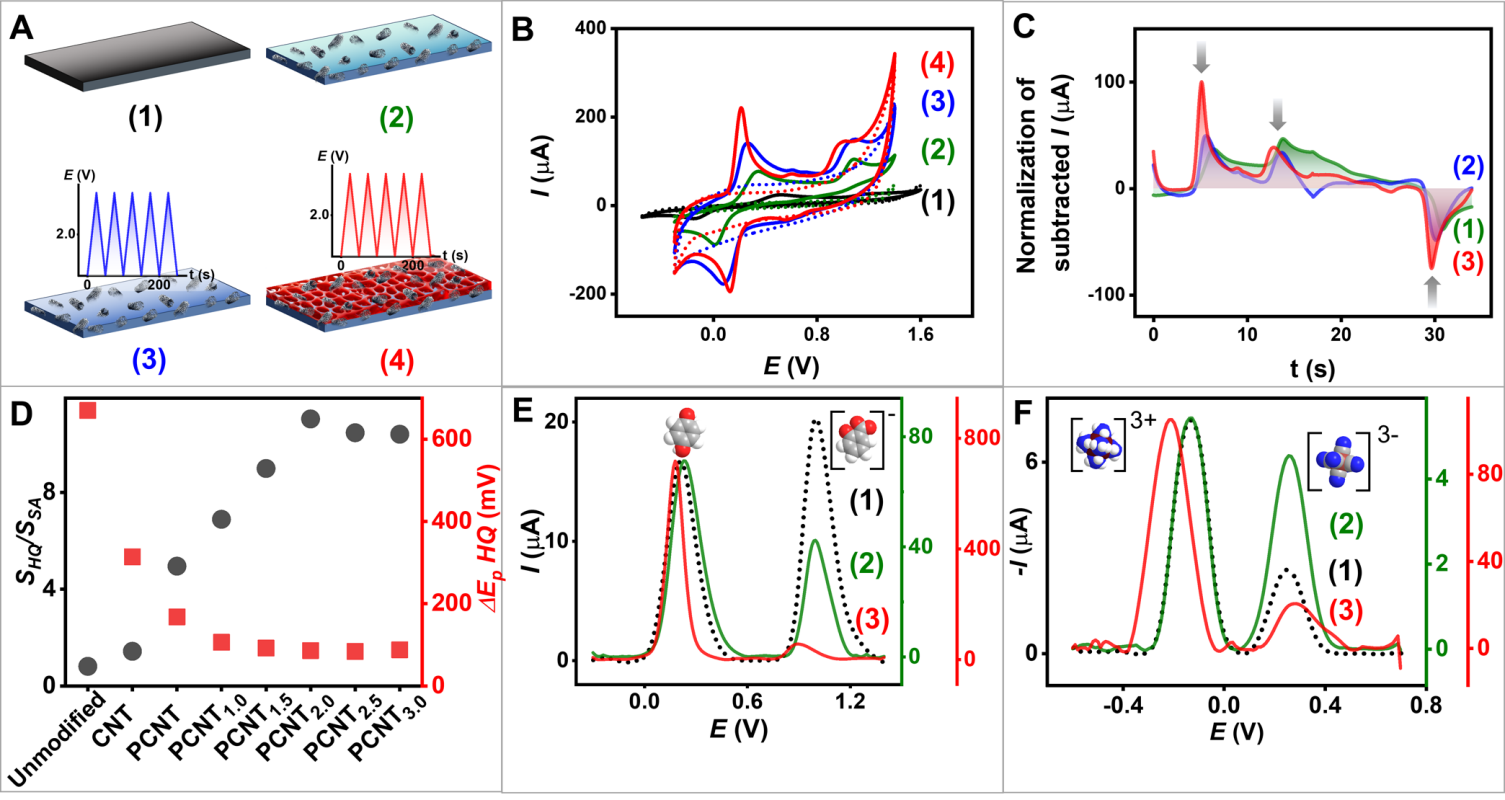
Engineering selective effects for detecting various electrochemically active species on printed electrodes. (A) Schematic illustration of (1) unmodified electrode, (2) CNT electrode, (3) CNT_2.0_ electrode, and (4) PCNT_2.0_ electrode. (B) CV at a scan rate of 100 mV s^–1^ of (1) unmodified electrode, (2) CNT electrode, (3) CNT_2.0_ electrode, and (4) PCNT_2.0_ electrode. The dotted line represents the solution without HQ and SA, while the solid line represents the solution containing 500 μM HQ and 500 μM SA. (C) Normalized subtracted current versus time plot of (1) CNT electrode, (2) CNT_2.0_ electrode, and (3) PCNT_2.0_ electrode. (D) Relationship between sensitivity ratio of HQ to SA and Δ*E*_p_ HQ versus various electrodes. (E) Background-subtracted square wave voltammograms (SWVs) in a solution containing 500 μM HQ and 500 μM SA of (1) unmodified electrode, (2) CNT electrode, and (3) PCNT_2.0_ electrode. (F) Background-subtracted SWVs in 0.1 M KCl containing 100 μM [Ru­(NH_3_)_6_]^3+^ and 100 μM [Fe­(CN)_6_]^3–^ in the reduction direction of (1) unmodified electrode, (2) CNT electrode, and (3) PCNT_2.0_ electrode (Parameters for panel E and F: step potential of 10 mV, amplitude of 75 mV, frequency of 5 Hz).

Figure 4B illustrates the CV responses obtained from the four electrodes tested in a mixture of HQ and SA. To evaluate electrochemical responses from each CV, the peak current was subtracted with electrochemical response in the solution without HQ and SA (yielding subtracted *I*_p_). Compared to the unmodified electrode, the CNT electrode exhibited a significant decrease in Δ*E*_p_ for HQ by approximately 332 mV (a 50% decrease from 0.670 to 0.338 V), indicating improved HQ detection (comparing Figure 4B­(1) and (2)). The CNT electrode also showed enhanced catalytic currents, with a 272–283% increase in both subtracted *I*_pa_ and *I*_pc_ for HQ and a 386% increase in *I*_pa_ for SA (Figure 4B­(2)) compared to the unmodified electrode. This suggests enhanced catalytic activity of the incorporated CNTs for detecting HQ and SA.

Anodizing the CNT electrode to create the CNT_2.0_ electrode further decreased Δ*E*_p_ for HQ by 59% (a 198 mV decrease from 0.338 to 0.140 V) (Figure 4B­(3)). The subtracted I_pa_ and I_pc_ for HQ, as well as *I*_pa_ for SA, increased to 96, 92, and 60 μA, respectively, representing 22–35% increase in subtracted *I*_pa_ and *I*_pc_ for HQ, and a subtracted 27% decrease in *I*_pa_ for SA compared to the pristine CNT electrode.

Compared to the unmodified electrode, the CNT_2.0_ electrode shows a significant decrease in Δ*E*_p_ for HQ of 79%, with an enhanced *I*_pa_ and *I*_pc_ for HQ of 378–391 and an enhanced *I*_pa_ for SA of 289%. The combination of CNTs and anodization in the CNT_2.0_ electrode indicates enhanced electrochemical signals in terms of decreased Δ*E*_p_ for HQ and increased peak current for both HQ and SA compared to the unmodified electrode (see Figure 4B­(3) and B(1)).

Furthermore, the PCNT_2.0_ electrode (Figure 4B­(4)) showed the lowest Δ*E*_p_ of 0.110 V for HQ, an 84% decrease compared to the unmodified electrode, with clearly increased *I*_pa_ and *I*_pc_ for HQ of 144–191 μA (around 648–880% improvement). However, for *I*_pa_ for SA, the PCNT_2.0_ electrode exhibits an increase of 380% compared to the unmodified electrode. The combination of the porous structure and anodization of the PCNT_2.0_ electrode significantly facilitates electron transfer for HQ, whereas the negative charge on the engineered surface repels negatively charged species, leading to a smaller increase in sensitivity toward SA oxidation.

Electrochemical surface anodization enables the programmable adjustment of electrochemical properties and selective interactions with chemical species. Future studies will systematically investigate the impact of different anodization potentials on the electrochemical performance of electrodes. Therefore, we employed a range of anodization potentials spanning from 0.75 to 3.25 V. The CV profiles of various electrodes were recorded in 1.0 M Na_2_CO_3_ to study the onset oxidation involving anodization treatment (as shown in Figure S20). The unmodified electrode exhibited a sharp onset of the oxidation anodic wave in the CV curve at 1.28 V (Figure S20A), while the CNT electrode showed an onset rise at a slightly lower potential of 1.26 V (Figure S20B), and the PCNT electrode also displayed a lower oxidation wave onset at 1.17 V (Figure S20C). The sharp increase in oxidation current (Figure S20A–C) correlates with the generation of gas bubbles during anodization. The enhanced current response of the PCNT electrode during the anodization process, compared to other electrodes, confirms an increased active surface area due to its porous structure. These bubbles and anodic current result from water electrolysis and the oxidation of electrode materials, leading to the formation of oxygen gas, protons, and electrons, as well as introducing oxygen-containing groups.^27,60,61^ The increase in the anodization potential window correlates with an increased anodic current response and fluctuations in CVs during anodization, particularly at high anodic potential windows (Figure S21). Such noisy fluctuations are due to gas bubbles generated during surface modification.

After anodization of electrode surfaces, these anodized electrodes were utilized to detect the redox reaction of HQ and the oxidation of SA for studying the effect of surface engineering on the electrochemical performance. The CVs and square wave voltammograms (SWVs) of PCNT electrodes in a solution containing a mixture of HQ and SA (Figures S22 and S23) further highlight the improved electrochemical properties of anodized electrodes.

Figure 4C illustrates the normalization of subtracted current over time for the electrodes, demonstrating their dynamic electrochemical responses in the mixed HQ and SA solution when sweeping at a constant rate of ramping up and down. The potential of each printed electrode was ramped up linearly from −0.30 to +1.40 V and then, at *t* = ∼17 s, reversed back toward −0.30 V. The results can be noted at arrows indicating the points corresponding to high HQ oxidation at around *t* = 6 s, SA oxidation at approximately *t* = 13 s, and HQ reduction at around *t* = 30 s.

The PCNT_2.0_ electrode exhibits sharp peaks showing the highest current and the most negative current responses at *t* = 5.1 and 29.7 s respectively, indicating enhanced sensitivity to HQ compared to the CNT and CNT_2.0_ electrodes. This enhancement is attributed to the surface-engineered structure of the PCNT_2.0_ electrode. Moreover, at time = 12.8 s, the negatively charged surface of the CNT_2.0_ and PCNT_2.0_ electrodes leads to a decreased SA oxidative signal compared to the nonanodized CNT electrode.

Furthermore, simultaneous detection of HQ and SA at concentrations up to 500 μM was conducted to determine the slope relating the SWV peak current to concentration (Figures S23, S25, and S28). Subsequently, regression analysis was performed on the obtained data to derive the slope, providing information on the quantitative relationship between the SWV peak current and the concentration of HQ or SA. This slope was used to evaluate the ratio of sensitivities toward HQ to SA for each electrode. Figure 4D summarizes the sensitivity ratio toward HQ to SA (S_HQ_/S_SA_) as well as the Δ*E*_p_ HQ obtained from CVs (Figures S22, S24, and S25). Sensitivity was determined from the slope of the SWV peak current at concentrations up to 500 μM for both HQ and SA (Figures S23, S25, and S28). This analysis highlights the sensitivity of HQ and SA detection across different anodization potential windows and emphasizes the rejection of charge effects.

Anodization improves the preferential detection of HQ over SA because the generation of oxygenated species results in a negative charge. This negative charge repels SA, which also carries a negative charge. Comparing the HQ-to-SA sensitivity ratio (S_HQ_/S_SA_) obtained from unmodified material to the ratio obtained from the CNT electrode, we observed a significant enhancement in S_HQ_/S_SA_ by 77%. Notably, when comparing the S_HQ_/S_SA_ ratios between unmodified material and the PCNT_2.0_ electrode, the PCNT_2.0_ electrode exhibited an OFF state toward the detection of SA while demonstrating an ON state with an enhanced electrocatalytic signal for HQ oxidation. We found a remarkable 3398% increase in sensitivity toward HQ, whereas the sensitivity toward SA detection only increased by 208%, reflecting the preference to detect HQ and showing the rejection against SA molecules from the anodized porous surface. Moreover, the Δ*E*_p_ HQ of the PCNT_2.0_ electrode clearly decreased down to 0.086 V (from 0.670 V for the unmodified electrode). These results indicate that the anodization process significantly enhances the sensitivity and electron transfer efficiency of the electrodes, particularly for the detection of HQ while rejecting SA.

We studied the anodization potential windows to determine how such applied energy can tune electrode sensitivity and selectivity by evaluating S_HQ_/S_SA_ ratios. PCNT electrodes with varying midpoint potentials ranging from 1.0 to 3.0 V were examined. If the study aimed at detecting HQ, the optimal window of anodization could be defined as the range of potentials that maximizes S_HQ_ while minimizing S_SA_, leading to a maximized S_HQ_/S_SA_ ratio and minimized Δ*E*_p_ HQ of the electrodes. The suitable window was found to be between 1.75 and 2.25 V (with a midpoint potential of 2.0 V), which minimized the Δ*E*_p_ HQ to 0.086 V and maximized the S_HQ_/S_SA_ ratio to 11.0 due to the introduction of oxygenated groups and a porous structure. Higher anodization potential windows might lead to overoxidation and degradation of the electrode material, resulting in slightly decreased S_HQ_/S_SA_ ratios. The anodization voltage range was systematically optimized to balance surface functionalization and structural integrity. This optimization is attributed to the controlled formation of oxygen-containing groups on the porous surface. Anodization outside this range resulted in either under-functionalized surfaces at lower voltages or overoxidation and structural degradation at higher voltages, which negatively impacted both sensitivity and selectivity. Figure 4E illustrates the unmodified electrode, CNT electrode, and PCNT_2.0_ electrode, emphasizing the different S_HQ_/S_SA_ ratios. The combination of a porous structure and optimized anodization results in the selective detection of neutral HQ and rejection of negatively charged SA. The finely tuned electrode surface ensures enhanced charge transfer, lowering Δ*E*_p_ HQ, and increasing the S_HQ_/S_SA_ ratio for improved specificity toward HQ oxidation. The sensitivities obtained from SWV results align with the peak currents of both HQ and SA oxidations in CV studies. The voltammetric profiles in Figure 4E further highlight the selectivity achieved through this optimization. The PCNT_2.0_ electrode amplifies the signal for neutral analytes like HQ while suppressing the response for negatively charged analytes like SA, demonstrating the programmable surface properties essential for complex sample analysis.

Additionally, apart from the optimization of anodization condition to maximize HQ signal response while suppressing SA signal response, we studied the effect of pH on the HQ detection (Figure S8A). pH 7.0 was chosen for this study as it represents mild conditions. While at pH 8.0 produces a stronger peak current (Figure S8A) and lower peak potential (Figure S8B), pH 7.0 was selected to ensure compatibility with mild wearable devices, providing a safe and practical balance between performance and usability for real-world applications. Note that HQ could deprotonate in alkaline media leading to a decreased adsorption capacity of HQ on the electrode surface resulting in the decrease of peak current at pH 10.0.

To further study the charge-selective effect, we conducted an electrochemical analysis using square wave voltammetry (SWV) in standard redox probe solutions containing a cationic complex ([Ru­(NH_3_)_6_]^3+^) and/or an anionic complex ([Fe­(CN)_6_]^3–^). Figure 4F illustrates the SWV of the printed electrodes in a mixed solution of [Ru­(NH_3_)_6_]^3+^ and [Fe­(CN)_6_]^3–^ in the reduction direction. The PCNT_2.0_ electrode exhibits the largest difference in current response signals between the peaks of the cationic and anionic complexes, showing enhanced sensitivity to the cationic complex analyte and selective rejection of anionic complex species.

Additionally, to support our observations, we recorded three components in both the oxidation and reduction directions during each SWV scan: the response components (Δ*i*), forward current (*i*_f_), and backward current (*i*_b_). The charge effects of the electrodes in solutions containing either cationic or anionic complexes were investigated separately (Figure S29). To evaluate the electrochemical response from SWV, we quantified the baseline-subtracted peak current. The unmodified electrode displayed a small peak current of 6 μA for the cationic complex at a potential of −0.127 V, while the anionic complex showed a peak current of 3 μA at a potential of 0.286 V in the oxidation direction (Figure S29A). In the reduction direction, similar peak potentials were observed for both the cationic and anionic complexes (Figure S29D).

The CNT electrode (Figure S29B and S29E) exhibited a response signal similar to that of the unmodified electrode in both oxidation and reduction directions. Notably, the PCNT_2.0_ electrode showed a significant amplification in peak current, with levels of 292 μA for the cationic complex and 22 μA for the anionic complex in the oxidation direction (Figure S29C). A similar trend was observed in the reduction direction (Figure S29F). This confirms that electrode modification, particularly with a porous structure and anodization, substantially enhances sensitivity to cationic complexes while rejecting negatively charged complexes.

Furthermore, the study of electrochemical behaviors in mixed cationic and anionic complexes indicates that the porous structure and surface modification of the PCNT_2.0_ electrodes not only amplify the electrochemical signal (e.g., a large cathodic peak current for cationic complex species, a 1376% increase compared to the unmodified electrode) but also improve selectivity toward positively charged species in solutions containing mixed complexes (with only a small peak current for anionic complex species) indicating an OFF state toward the detection of anionic complex (Figure S30). The ability to switch between ON and OFF states based on the analyte’s charge enables the PCNT_2.0_ electrode to act as a versatile and programmable platform. This capability is particularly critical for applications in complex matrices, such as skincare products, where multiple electroactive species often coexist.

#### Electroanalytical Detection of HQ and SA Using the SWV Technique

We first investigated how adjusting SWV parameters—pulse amplitude and frequency—affects electroanalytical detection in a mixed solution of HQ and SA. The step potential remained constant at 15 mV, while we varied the pulse amplitude from 12.5 to 150 mV and frequencies from 2.5 to 30 Hz. Figure 5A illustrates the impact of changing pulse amplitude on peak current at different frequencies. Increasing the pulse amplitude led to a rise in peak current, enhancing electron transfer facilitation. However, broader peaks were also observed due to the larger capacitive background signal, indicating a trade-off between peak intensity and resolution.

**Figure 5:**
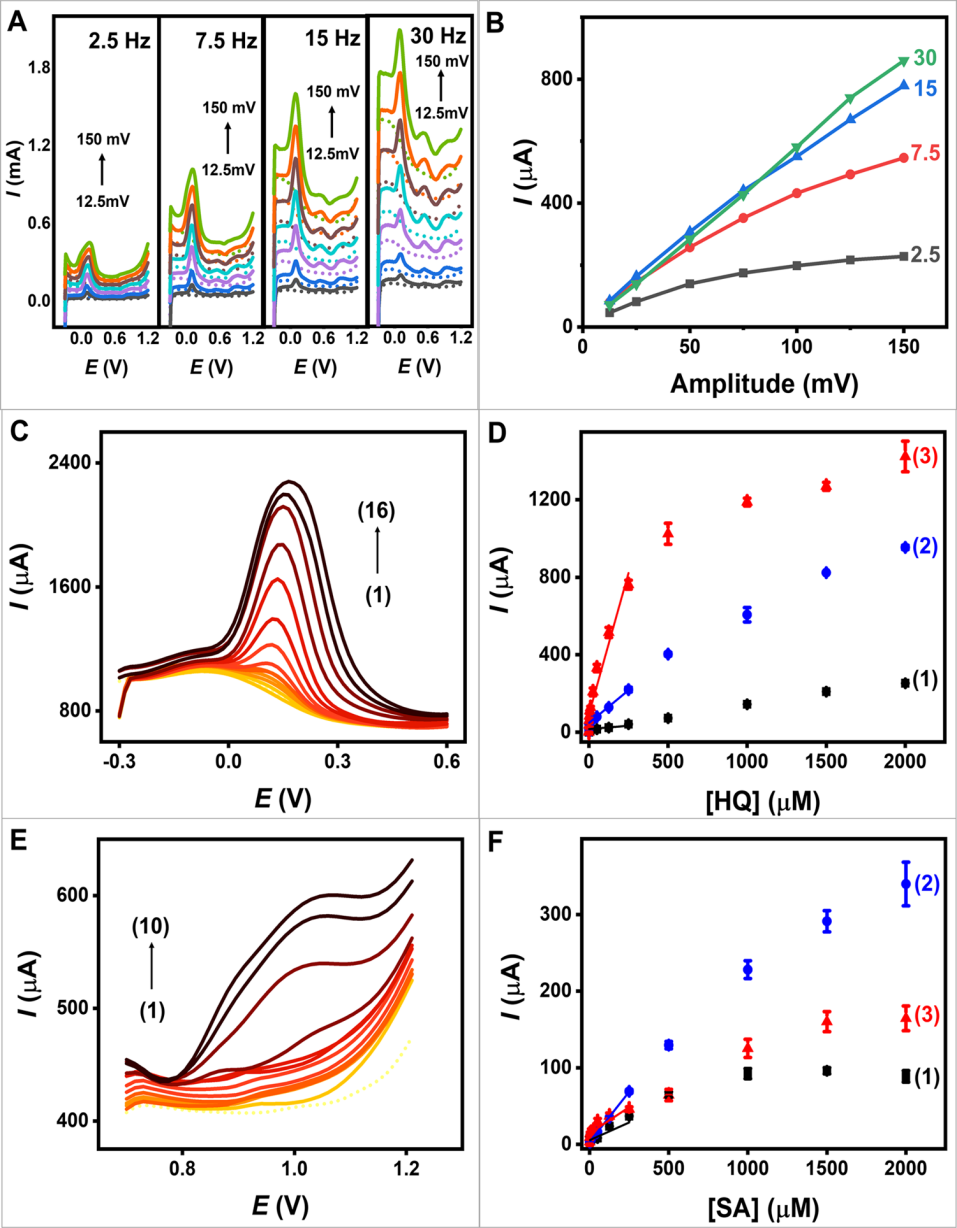
Electroanalytical detection of HQ and SA. (A) The effects of amplitudes and frequencies of PCNT_2.0_ electrode in 250 μM HQ and 500 μM SA by varying amplitude of 12.5, 25, 50, 75, 100, 125, and 150 mV with the step potential at 15 mV and the different frequencies (2.5, 7.5, 15, and 30 Hz). The short dot represents the background scans. (B) SWV current response of HQ for PCNT_2.0_ electrode at various amplitudes and frequencies. (C) SWVs of PCNT_2.0_ electrode (with a step potential of 15 mV, an amplitude of 125 mV, and a frequency of 15 Hz) in HQ with various concentrations: (1–16) 0, 0.1, 0.5, 1, 2.5, 3.75, 5, 10, 25, 50, 125, 250, 500, 1000, 1500, and 2000 μM. (D) Calibration curves in the presence of 0.1 to 250 μM HQ of (1) unmodified electrode, (2) CNT_2.0_ electrode, and (3) PCNT_2.0_ electrode. Error bars represent the standard deviations (*n* = 3). (E) SWVs of PCNT_2.0_ electrode (with a step potential of 15 mV, an amplitude of 125 mV, and a frequency of 15 Hz) in SA with various concentrations: (1–10) 0, 10, 25, 50, 125, 250, 500, 1000, 1500, and 2000 μM. (F) Calibration curves in the presence of 2.5 to 250 μM SA of (1) unmodified electrode, (2) CNT_2.0_ electrode, and (3) PCNT_2.0_ electrode. Error bars represent the standard deviations (*n* = 3).

Figure 5B summarizes the impact of SWV parameters on the current response of HQ. Based on the findings, we identified the optimal SWV parameters as a step potential of 15 mV, a pulse amplitude of 125 mV, and a frequency of 15 Hz. These conditions offer a balance between achieving high sensitivity and maintaining resolution without significant peak broadening or distortion. Additionally, these conditions enable fast analysis, with an effective scan rate of 225 mV s^–1^, allowing for a short detection time of less than 7 s. These optimized SWV parameters will be applied in further electrochemical characterization.

#### HQ and SA Detection Using Optimized SWV Parameters

We utilized SWV to quantitatively measure HQ for evaluating sensitivity obtained from calibration curve. Figure 5C shows the SWV responses of the PCNT_2.0_ electrode, while Figure S31 displays the responses of the unmodified electrode, and Figure S32 presents the responses of the CNT_2.0_ electrode in the solution containing HQ with concentration, ranging from 0.1 to 2000 μM. We found that the peak current response increases with concentration of HQ. The calibration curve of HQ is presented in Figure 5D.

We calculated the limit of detection (LOD) using the eq 3S_b_/m, where *S*_*b*_ represents the standard deviation of the blank, and *m* is the slope of the calibration curve.^62^ We found that the LOD of the PCNT_2.0_ electrode is 0.046 μM. Over the concentration range of 1–250 μM, the sensitivity of HQ detection increases by 568% for the CNT_2.0_ electrode (0.682 ± 0.009 μA μM^–1^) and significantly by 2624% for the PCNT_2.0_ electrode (2.779 ± 0.228 μA μM^–1^) compared to the unmodified electrode, which exhibits a low sensitivity of 0.102 ± 0.013 μA μM^–1^. This substantial increase in sensitivity is attributed to favorable electron transfer and an enhanced electrochemically active surface area in the PCNT_2.0_ electrode.

Comparing our electrode for HQ detection to other existing arts (Table S2), the PCNT_2.0_ electrode offers a reasonable LOD and a wide detection range (0.1 to 2000 μM). Advantageously, our CNT-modified ink allows for printability on flexible substrates. Furthermore, unlike traditional rigid electrodes, our porous electrode offers both flexibility and wearability. These properties are achieved through screen-printing followed by selective etching, highlighting features that other sensors do not address. These characteristics make our electrode suitable for a wide range of applications, providing a robust and sensitive tool for on-site and on-body monitoring, as well as a versatile platform for flexible electronic devices.

Figure 5E shows the SWV responses of the PCNT_2.0_ electrode, while Figures S33 and S34 display the responses of the unmodified and CNT_2.0_ electrodes, respectively, in SA solutions. Surface engineering via anodization achieves a negatively charged surface on the electrodes, which tends to repel negatively charged SA from the electrode surface. This repulsion is evident in the LOD of the PCNT_2.0_ electrode for SA detection, which is higher at 8.51 μM compared to the unmodified electrode (LOD of 2.64 μM). When comparing our electrode for SA detection to others, the PCNT_2.0_ electrode exhibited a comparable LOD with a detection range of 10 to 2000 μM. Additionally, it offers desirable aspects, such as a flexible, porous platform that can be fabricated using a screen-printing technique.

#### Interference Study

When detecting analytes in complex cosmetic samples, interference from matrix components is a critical concern, as it can lead to false peaks and inaccurate results. This necessitates careful attention to ensure reliable measurements. This study examines the SWV electrochemical signals of the PCNT_2.0_ electrode in solutions containing substances commonly added to skincare products to assess potential interference effects. The results, presented in Figure S35, demonstrate the selectivity of the PCNT_2.0_ electrode in various solutions, such as riboflavin, ascorbic acid (AA), Na^+^, Mg^2+^, K^+^, Zn^2+^, Fe^3+^, Cu^2+^, Ca^2+^, Cl^–^, CO_3_^2–^, H_2_O_2_, and ethylenediaminetetraacetic acid, no observable peaks interfered with the detection. These findings confirm that the PCNT_2.0_ electrode, when used with the optimized SWV technique, allows for the selective detection of HQ and SA without interference from common skincare ingredients.

### Application of the Printed Flexible Porous Electrode on a Wearable Glove for Skincare Sample Screening and Its Use in Glucose Biosensor Development

#### Integrated Wearable HQ and SA Sensor for Cosmetic Sample Analysis

To demonstrate its practical utility in scenarios such as on-demand skincare sample screening, we fabricated a flexible PCNT_2.0_ electrode on a wearable glove. Figure 6A depicts the flexible electrodes, including the PCNT_2.0_ electrode (working electrode), the CNT electrode (counter electrode), and the reference electrode, printed on the index fingertip surface of the wearable glove, with the thumb fingertip attached with a PVA-electrolyte cryogel. Real samples spiked with varying amounts of HQ and SA were tested, as illustrated in Figure 6B, revealing clear and distinct oxidation peaks for HQ and SA (Figure 6B­(1–4)). The results indicate that with increasing concentrations of spiked HQ and SA, the peak currents also increase, demonstrating a proportional relationship between concentration and current response. Furthermore, mixtures containing both HQ and SA were tested to evaluate the electrode’s capability for simultaneous detection (Figure 6B­(5–6)). The SWV responses displayed combined oxidation peaks at the expected potentials. This lab-on-a-glove sensing approach underscores the PCNT_2.0_ electrode’s ability to perform successful simultaneous multianalyte detection, which is crucial for practical applications in rapid skincare sample screening.

**Figure 6:**
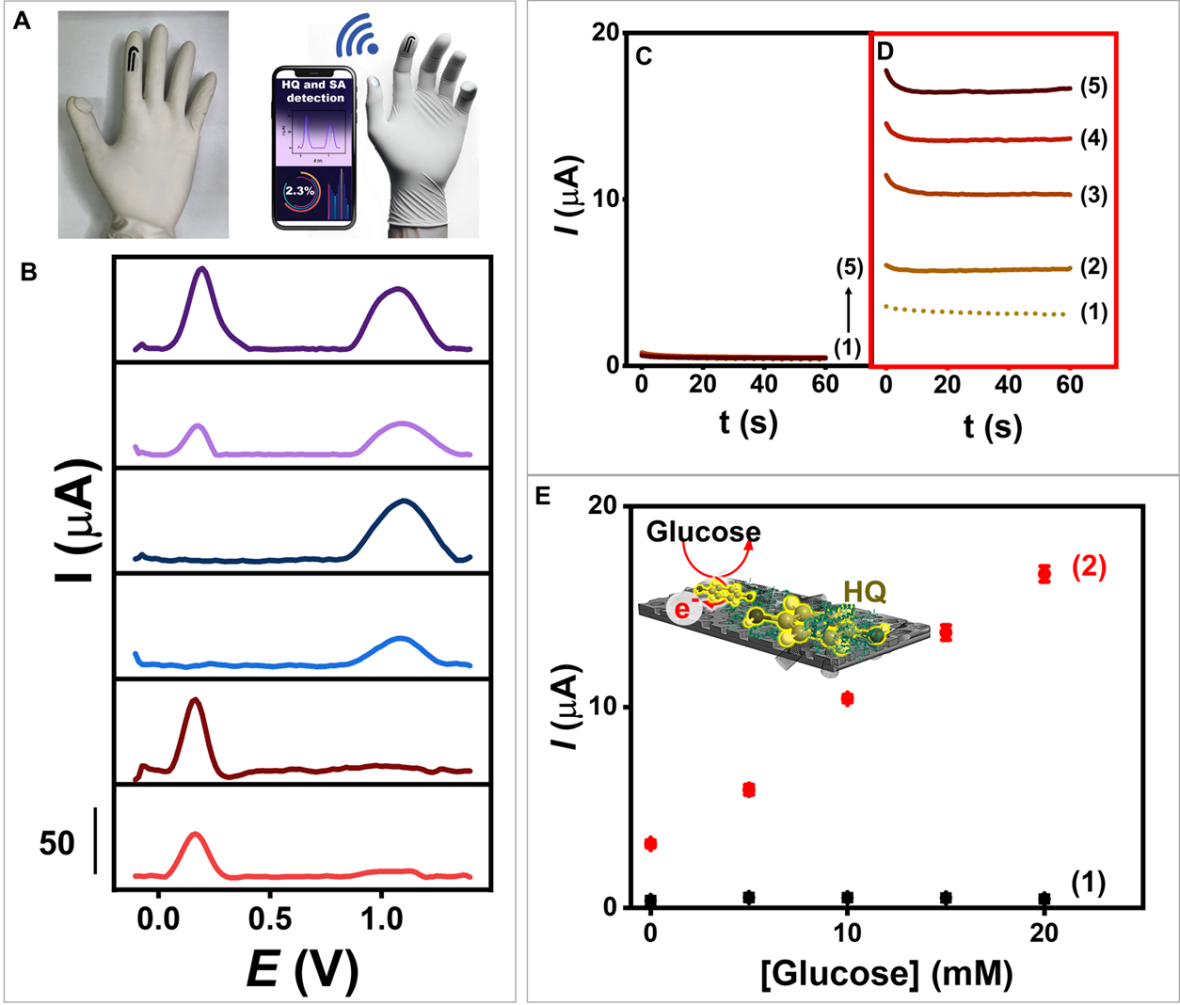
PCNT_2.0_ electrode on a wearable glove for screening of skincare products and its use in the glucose biosensor. (A) Photograph and the concept of screen-printed electrodes on a wearable glove for skincare sample screening. (B) Baseline-subtracted SWV of the PCNT_2.0_ electrode for on-demand detection of HQ and SA on a glove fingertip using PVA-electrolyte support wiped with real samples spiked with (1) 0.5% HQ, (2) 1.0% HQ, (3) 2.5% SA, (4) 5.0% SA, (5) 0.5% HQ and 2.5% SA, and (6) 1.0% HQ and 5.0% SA. (C–D) Amperograms in solutions containing various glucose concentrations: (1–5) 0, 5, 10, 15, and 20 mM obtained from (C) GOx/HQ/unmodified electrode and (D) GOx/HQ/PCNT_2.0_ electrode. Applied potential of 0.4 V. (E) Corresponding calibration plot of the current response and glucose concentrations for (1) GOx/HQ/unmodified electrode and (2) GOx/HQ/PCNT_2.0_ electrode.

We also evaluated the printed electrode designed for the electrochemical detection of SA in real samples. The SA in a skincare sample was taken out using a buffer solution, resulting in a solution containing the dissolved SA. Measurements using our printed PCNT_2.0_ electrodes showed a concentration of 303.8 ± 8.4 μM SA (0.55 ± 0.02%). To validate this result, the standard titration method was employed to determine the total acid content in the skincare product sample. The titration revealed 573.7 μM of acids in the supernatant extracted from the skincare product. The SA content in the sample was estimated to be 1.03 ± 0.12% (*n* = 3). The difference between the titration method and the electrochemical analysis can be attributed to the fact that titration measures the total acid content, which may include other acids present in the skincare product, resulting in a relatively higher concentration. In contrast, our SWV technique detects only SA. This comparative analysis demonstrates the effectiveness of our method for rapid screening of skincare samples, offering advantages in cost-effectiveness and suitability for field applications compared to traditional methods.

We also conducted a recovery determination experiment to evaluate the sensor’s effectiveness in quantifying the analytes within a complex sample matrix. Standard solutions of HQ and SA were spiked to the samples, and their concentrations were analyzed using the sensor. Recovery percentages were then considered. When spiking with 25 μM of HQ and SA, the CNT_2.0_ sensor exhibited recoveries of 109 ± 4% for HQ and 103 ± 1% for SA, while the PCNT_2.0_ sensor showed recoveries of 108 ± 7% for HQ and 108 ± 3% for SA. For spiking with 75 μM of HQ and SA, the CNT_2.0_ sensor demonstrated recoveries of 105 ± 6% for HQ and 106 ± 2% for SA, whereas the PCNT_2.0_ sensor achieved recoveries of 104 ± 13% for HQ and 102 ± 4% for SA. These results indicate the acceptable sensor’s performance in screening analytes from a complex matrix in skincare samples.

#### Application of the Printed Flexible Porous Electrode in Glucose Biosensor Development

Given the use of redox mediators in second-generation glucose biosensors for glucose detection, we propose leveraging the enhanced HQ adsorption capability of the surface-engineered porous nanocomposite PCNT_2.0_ electrode to enhance the performance of glucose enzymatic biosensors. The enhanced sensitivity for detecting HQ with the flexible printed PCNT_2.0_ electrode is attributed to its surface engineering, which increases surface area and creates a porous structure for maximizing HQ adsorption. We conducted a comparative study between the GOx/HQ/unmodified electrode and GOx/HQ/PCNT_2.0_ electrode, measuring current responses across various glucose concentrations via amperometry (Figure 6C,D, respectively).

The GOx/HQ/PCNT_2.0_ electrode demonstrated increasing current responses with glucose concentrations (sensitivity of 0.699 μA mM^–1^), whereas the GOx/HQ/unmodified electrode showed unchanged current responses (Figure 6E). This highlights the importance of enhanced HQ redox mediator and the effective accumulation of GOx enzyme biomolecules on the electroactive electrode surface to improve the performance of mediator-based second-generation glucose biosensors. Specifically, the current response of the GOx/HQ/PCNT_2.0_ electrode showed a 3662% enhancement compared to the GOx/HQ/unmodified electrode at a glucose concentration of 20 mM. These findings highlight the potential for advancing biosensors using surface-engineered porous nanocomposite electrodes, showing their versatility in developing enzymatic sensing methodologies.

## Conclusions

We fabricated screen-printed, flexible porous nanocomposite electrodes using a low-cost, scalable strategy to enhance their electrochemical performance. By integrating CNTs into the ink and introducing porosity, we increased the electrochemically active surface area, which improved mass and charge transport. Surface area analysis showed a 214% increase compared to plain electrodes, aligning with improved surface morphology and reduced charge transfer resistance. Systematic adjustments during surface engineering allowed programmable control over electrochemical properties. We achieved a sensitivity ratio of HQ to SA greater than 10, compared to less than 1 for plain electrodes, enabling precise “ON” or “OFF” detection of target molecules—essential for analyzing complex samples with multiple species. For instance, the surface-engineered electrode demonstrated enhanced sensitivity for HQ detection, with charge transfer kinetics and catalytic rate constants improved by 3 orders of magnitude compared to the plain electrode. The electrodes also exhibited mechanical resilience under repeated bending cycles. This capability was applied in developing a successful wearable platform for screening skincare cream samples. Additionally, the high-performance porous PCNT_2.0_ electrode showed potential for sensitive second-generation glucose enzymatic biosensors using the HQ redox mediator. This work represents a promising, economical, and facile fabrication strategy that advances flexible electrode performance and optimizes the nanocomposite surface for diverse applications, including electroanalytical detection and electrochemical energy applications. The integration of the PCNT_2.0_ electrode with custom-designed, miniaturized potentiostats could further enhance its potential for on-demand detection systems, thereby expanding its applicability in real-world scenarios.

## Supplementary Material



## Notes

Descriptions of the chemicals and reagents used, the preparation of CNT and PCNT electrode inks, and the fabrication of both CNT and anodized PCNT electrodes. Additionally, the preparation of PVA cryogel and skincare cream samples is outlined, along with the fabrication of flexible porous electrodes integrated into a wearable glove for application studies. The titration protocol for determining SA in skincare samples, as well as the preparation of electrodes for glucose biosensor development, is also included. Electrochemical studies and characterizations (PDF)
