# Supplementary material for: Tuning the Surface: Screen-Printed Flexible Porous Nanocomposite Electrodes with Programmable Electrochemical Performances for Wearable Platforms
Source: ACS Sens. 2025 Feb 28;10(6):3976–92. doi: 10.1021/acssensors.4c03519 (PMC12210251; doi:10.1021/acssensors.4c03519)
Supplement: Supplementary file 1 [file se4c03519_si_001.pdf]

Supporting Information

Tuning Surface: Screen-Printed Flexible Porous Nanocomposite Electrodes with Programmable Electrochemical Performances for Wearable Platforms

Adisak Pokprasert[1,4], Natcha Rasitanon[1,2,4], Irlesta Rahma Lani[1,4], Itthipon Jeerapan[1,2,3,4,*]

[1]Center of Excellence for Trace Analysis and Biosensor, Prince of Songkla University, Hat Yai, Songkhla 90110, Thailand

[2]Division of Physical Science, Faculty of Science, Prince of Songkla University, Hat Yai, Songkhla 90110, Thailand

[3]Center of Excellence for Innovation in Chemistry, Faculty of Science, Prince of Songkla University, Hat Yai, Songkhla 90110, Thailand

[4]The ijE Electrochemistry for All Laboratory, Prince of Songkla University, Hat Yai, Songkhla 90110, Thailand

*Correspondence: itthipon.j@psu.ac.th

ORCID of Itthipon Jeerapan: *https://orcid.org/0000-0001-8016-6411*

Supporting Information

Contents

1. Supporting figures 3

2. Supporting tables 28

3. Supporting notes 33

3.1. Note S1: Randles-Sevcik relationship 33

3.2. Note S2: Apparent capacitance 33

3.3. Note S3: Areal capacitance 33

3.4. Note S4: The number of electrons involved in the reaction 33

3.5. Note S5: Formal potentials 34

3.6. Note S6: Electron transfer constant 34

3.7. Note S7: Catalytic rate constant 34

3.8. Note S8: The *b*-value 34

4. Supporting experimental procedures 35

4.1. Chemicals and reagents 35

4.2. Preparation of CNT electrode ink and CNT electrode 35

4.3. Preparation of PCNT electrode ink and PCNT electrode 36

4.4. Anodization of PCNT electrodes 36

4.5. Fabrication of PVA cryogel 37

4.6. Preparation of skincare cream sample 37

4.7. Fabrication of flexible porous electrodes on a wearable glove and its application 37

4.8. Titration protocol for determining SA in the skincare sample 37

4.9. Preparation of $PCNT_{2.0}$-HQ-GOx electrode and glucose biosensor 38

4.10. Electrochemical Studies and Characterizations 39

5. Supporting References 39

1. Supporting figures

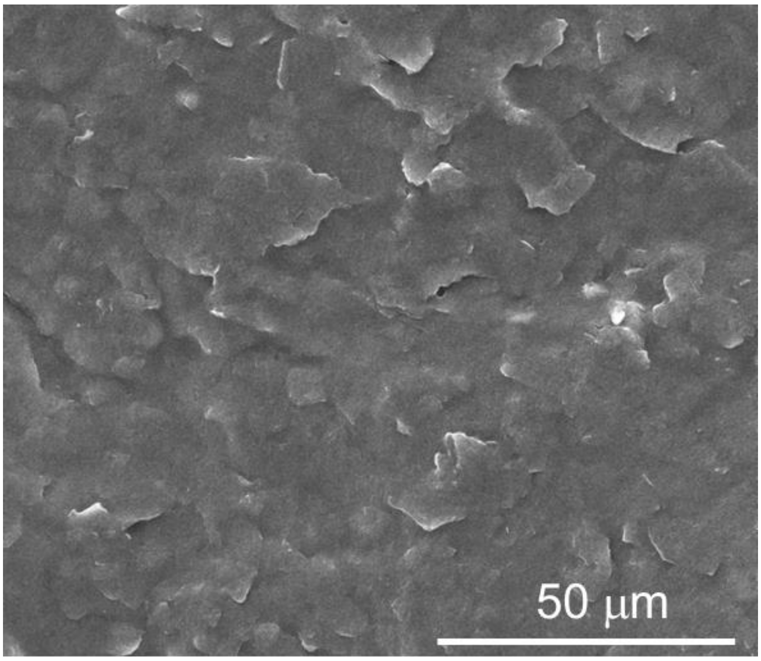

Figure S1. SEM image of the unmodified electrode.

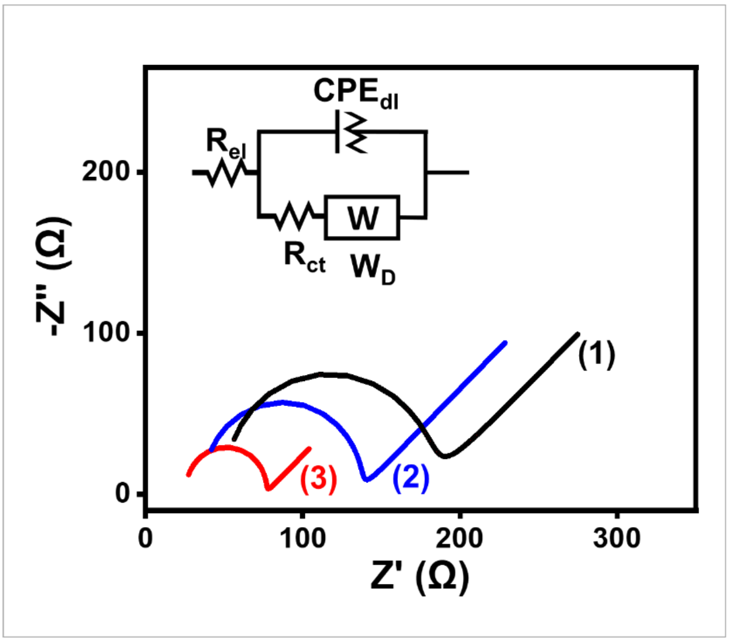

Figure S2. Fitted Nyquist plots of (1) unmodified electrode, (2) CNT electrode, and (3) $PCNT_{2.0}$ electrode in a solution of 10 mM $Ru(NH_3)_6Cl_3$ in 0.1 M KCl solution. The inset shows the fitted circuit of the EIS plot.

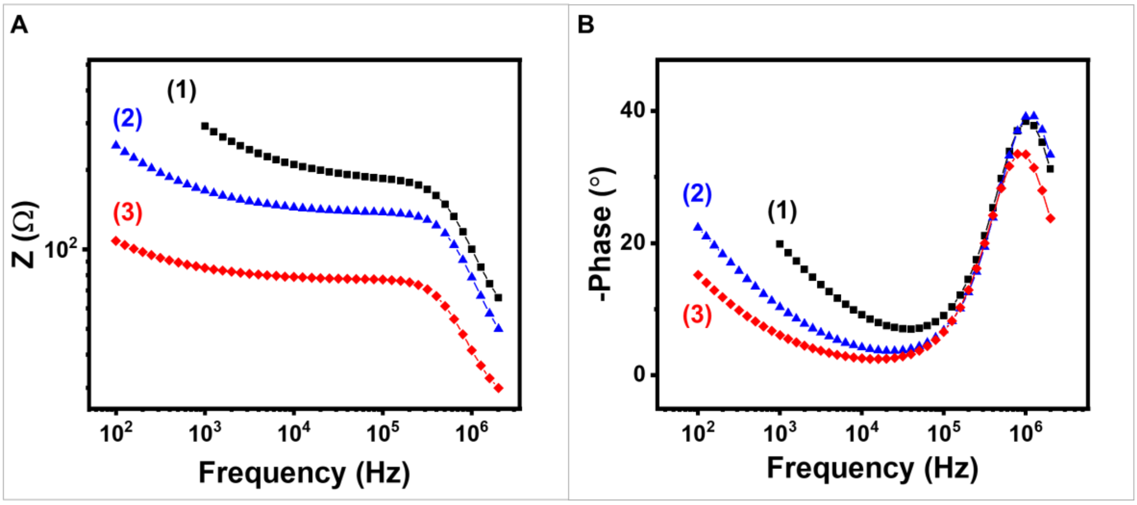

Figure S3. EIS plots of (1) unmodified electrode, (2) CNT electrode, and (3) $PCNT_{2.0}$ electrode in a mixed solution of 10 mM $Ru(NH_3)_6Cl_3$ in 0.1 M KCl solution, using an amplitude of 10 mV, 0.05 V DC for unmodified electrode with a frequency range of 1×10^3 – 2×10^6 Hz, 0.05 V DC for CNT electrode and $PCNT_{2.0}$ electrode with a frequency range of 1×10^2 – 2×10^6 Hz; (A) Bode plots. (B) phase angle versus frequency plots.

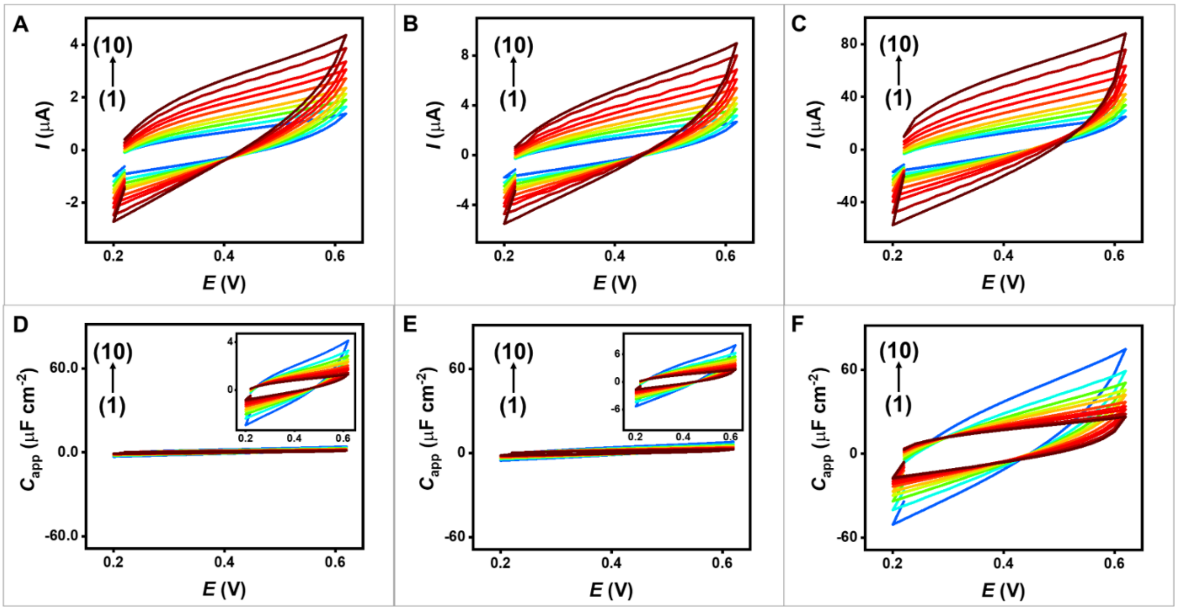

Figure S4. (A-C) CVs of a screen-printed flexible electrode at various scan rates ranging from 50 to 500 mV s^{-1}. The scan rates (1–10) used were 50, 75, 100, 125, 150, 200, 250, 300, 400, and 500 mV s^{-1} for (A) unmodified electrode, (B) $CNT_{2.0}$ electrode, and (C) $PCNT_{2.0}$ electrode. (D-F) Apparent capacitance profiles at various scan rates ranging from 50 to 500 mV s^{-1} for (D) unmodified electrode, (E) $CNT_{2.0}$ electrode, and (F) $PCNT_{2.0}$ electrode.

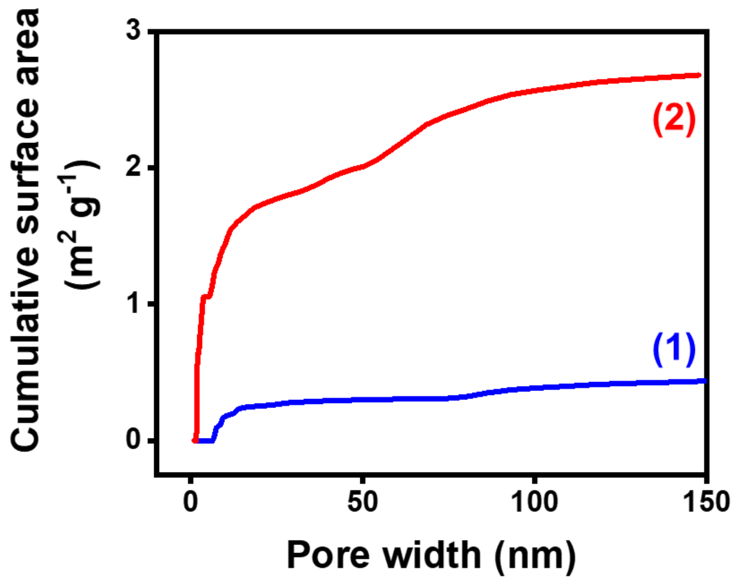

Figure S5. Relationship between cumulative surface area and pore width for (1) CNT electrode and (2) the top layer material of the $PCNT_{2.0}$ electrode.

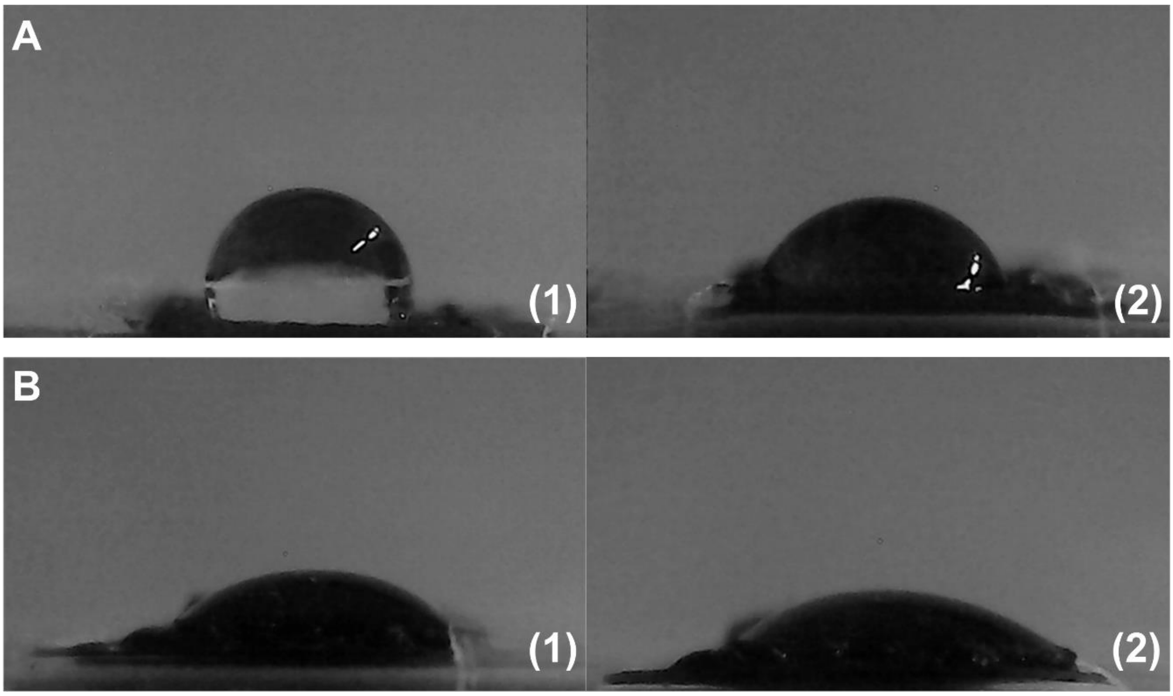

Figure S6. Water droplet contact angles on (A1) CNT electrode, (A2) $CNT_{2.0}$ electrode, (B1) PCNT electrode, and (B2) $PCNT_{2.0}$ electrode.

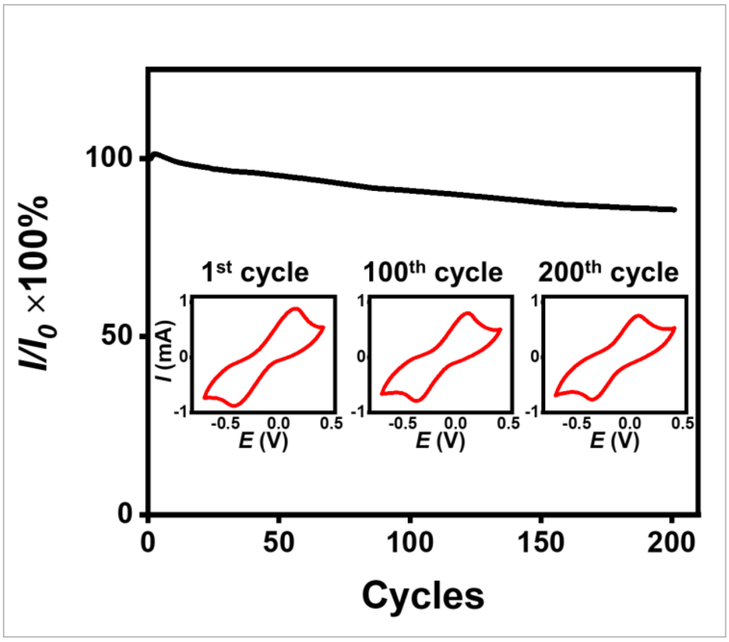

Figure S7. Stability plot of PCNT2.0 electrode at various cycle obtained from the CV in the solution containing 10 mM $[Ru(NH_3)_6]^{3+}$ in 0.1 M KCl with a scan rate of 100 mV s^{-1}. The inset shows corresponding CVs at 1st, 100th, and 200th cycle.

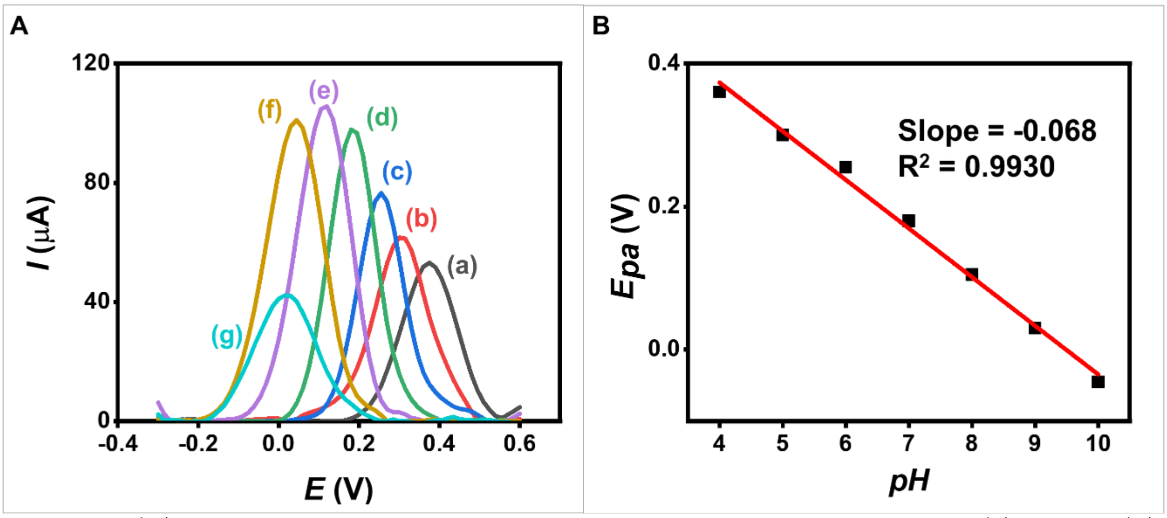

Figure S8. (A) SWVs of PCNT2.0 electrode in 500 μM HQ at different pH values; (a) pH 4.0, (b) pH 5.0, (c) pH 6.0, (d) pH 7.0, (e) pH 8.0, (f) pH 9.0, and (g) pH 10 of 0.1 M buffer solution at a step potential of 15 mV, an amplitude of 125 mV, and a frequency of 15 Hz. (B) The corresponding plot of potential peak (V) vs. pH.

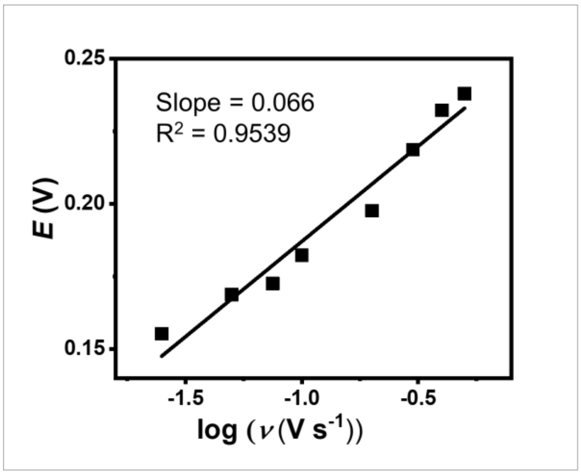

Figure S9. Anodic peak potential plot of the $PCNT_{2.0}$ electrode in 500 μM HQ in function with the log of scan rate (scan rates: 25, 50, 75, 100, 200, 300, 400, and 500 mV s^{-1}) were obtained on the $PCNT_{2.0}$ electrode.

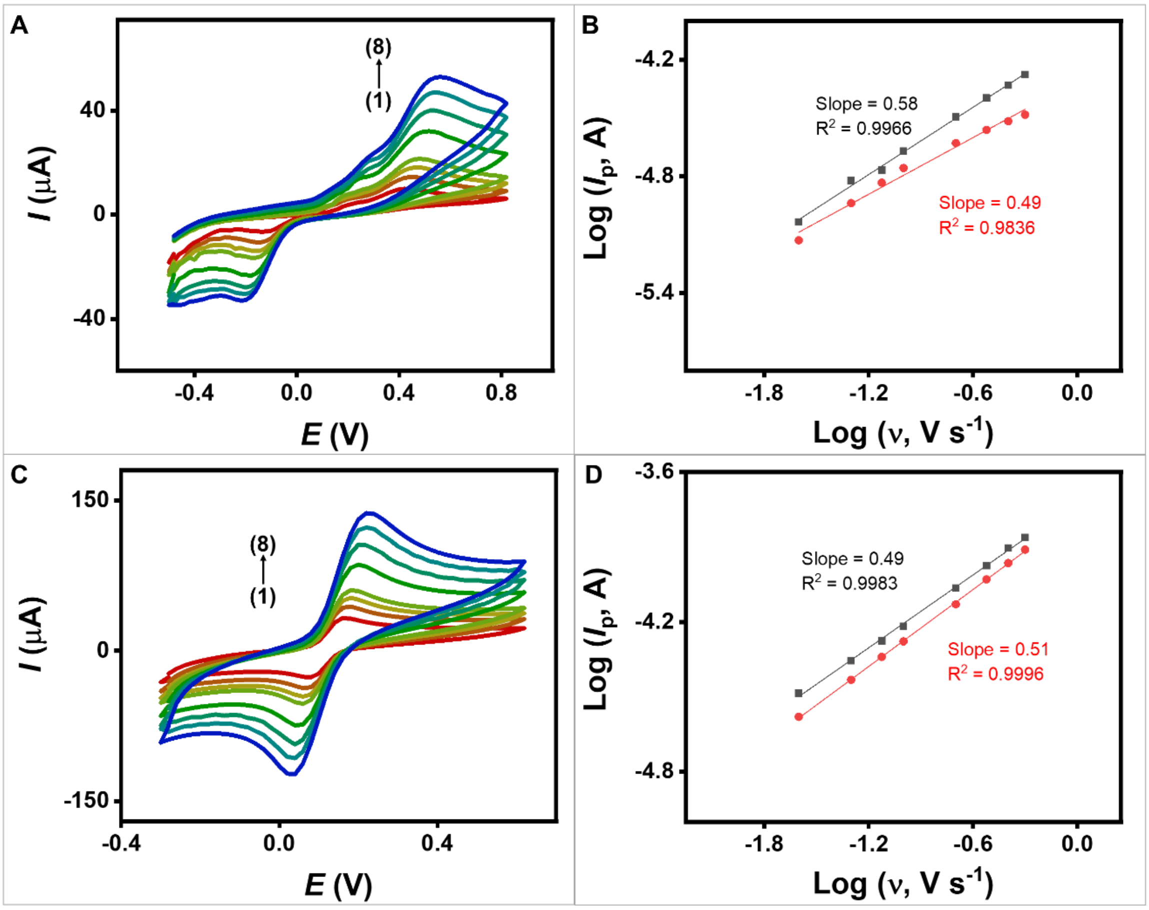

Figure S10. (A) CV recorded in 500 μM HQ of unmodified electrode with various scan rates: (1-8) 25, 50, 75, 100, 200, 300, 400, and 500 mV s^{-1}. (B) Logarithmic scale plot of peak current versus scan rate of unmodified electrode in 500 μM HQ. The black square represents the anodic peaks whereas the red dot represents the cathodic peaks. (C) CV recorded in 500 μM HQ of $CNT_{2.0}$ electrode with various scan rates: (1-8) 25, 50, 75, 100, 200, 300, 400, and 500 mV s^{-1}. (D) Logarithmic scale plot of peak current versus scan rate of $CNT_{2.0}$ electrode in 500 μM HQ. The black square represents the anodic peaks whereas the red dot represents the cathodic peaks.

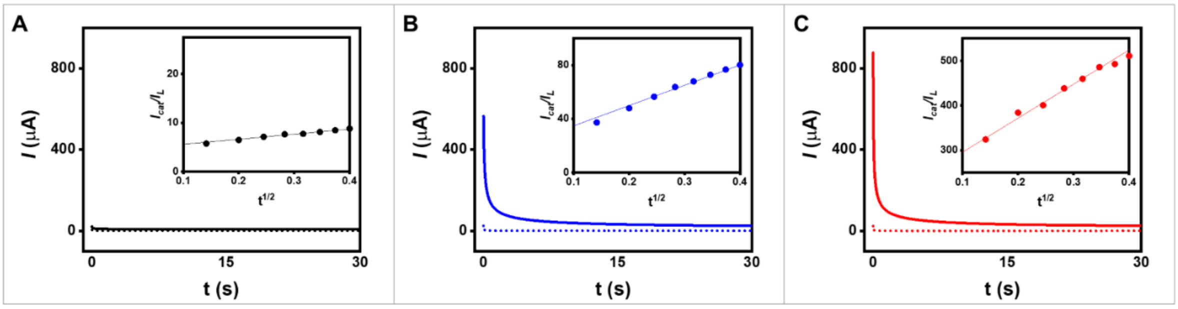

Figure S11. Amperometry plots at a potential of 0.30 V for (A) unmodified electrode, (B) $CNT_{2.0}$ electrode, and (C) $PCNT_{2.0}$ electrode in 0 μM (short dot) and 250 μM (solid line) of HQ. Inset provides the relationship between I_{cat}/I_L and $t^{1/2}$.

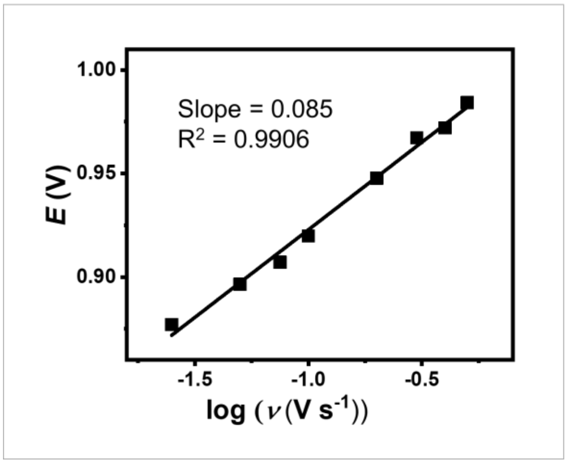

Figure S12. Anodic peak potential plot of the $PCNT_{2.0}$ electrode in 2000 μM SA in function with the log of scan rate (scan rates: 25, 50, 75, 100, 200, 300, 400, and 500 mV s^{-1}) were obtained on the $PCNT_{2.0}$ electrode.

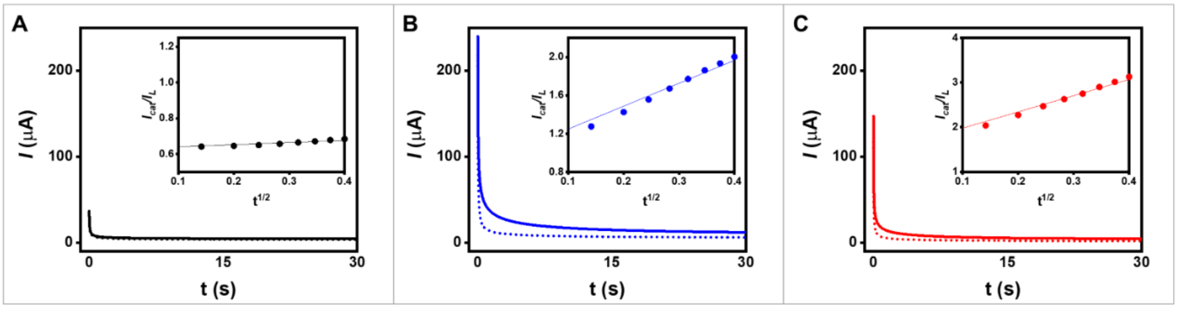

Figure S13. Amperograms obtained from (A) unmodified electrode and (B) $CNT_{2.0}$ electrode, and (C) $PCNT_{2.0}$ electrode with concentration of 0 μM (short dot) and 250 μM (solid line) of SA. Inset providing the relationship between I_{cat}/I_L and $t^{1/2}$. Applied potential of 1.0 V.

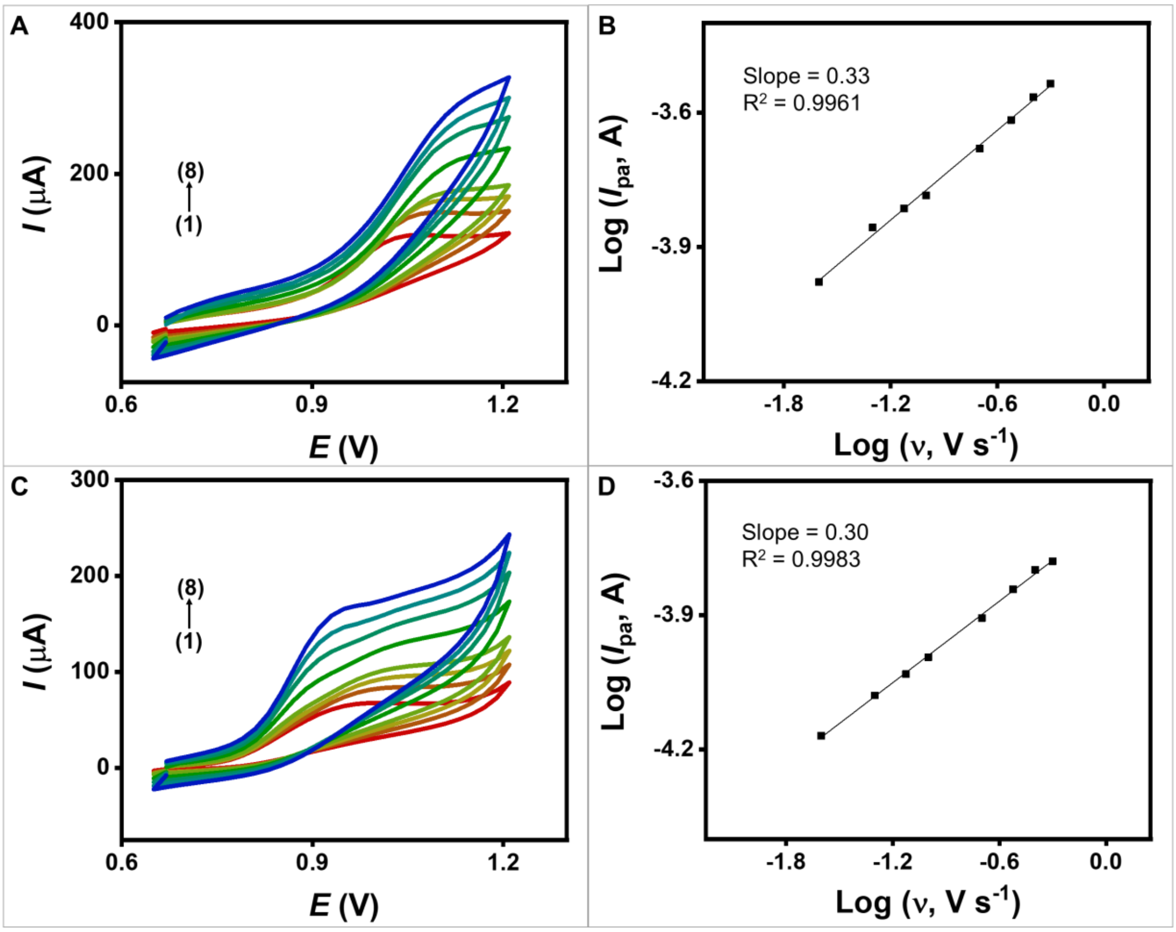

Figure S14. (A) CV recorded in 2000 μM SA of unmodified electrode with various scan rates: (1-8) 25, 50, 75, 100, 200, 300, 400, and 500 mV s^{-1}, (B) Logarithmic scale plot of absolute peak current versus scan rate of unmodified electrode in 2000 μM SA. The black square represents the anodic peaks, (C) CV recorded in 2000 μM SA of $CNT_{2.0}$ electrode with various scan rates: (1-8) 25, 50, 75, 100, 200, 300, 400, and 500 mV s^{-1}, and (D) Logarithmic scale plot of peak current versus scan rate of $CNT_{2.0}$ electrode in 2000 μM SA.

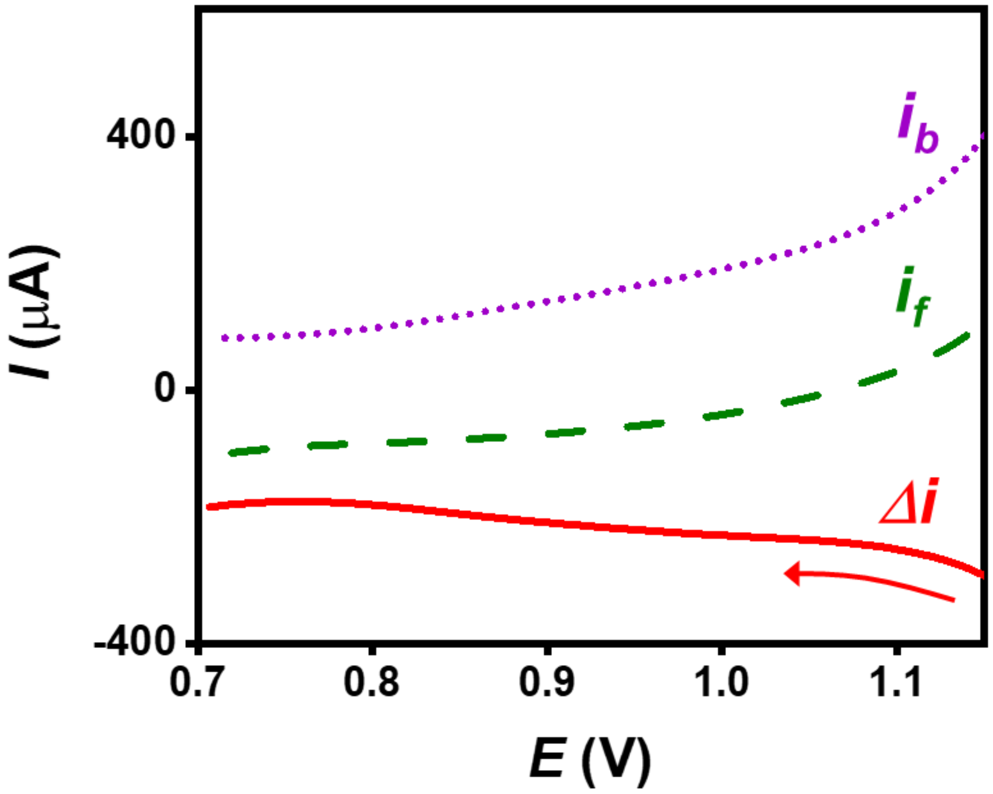

Figure S15. SWVs toward the detection of $PCNT_{2.0}$ electrode in 2000 μM SA toward reduction direction with the response (Δi), the forward (i_f), and backward (i_b) current components with a step potential of 10 mV, an amplitude of 75 mV, and a frequency of 5 Hz.

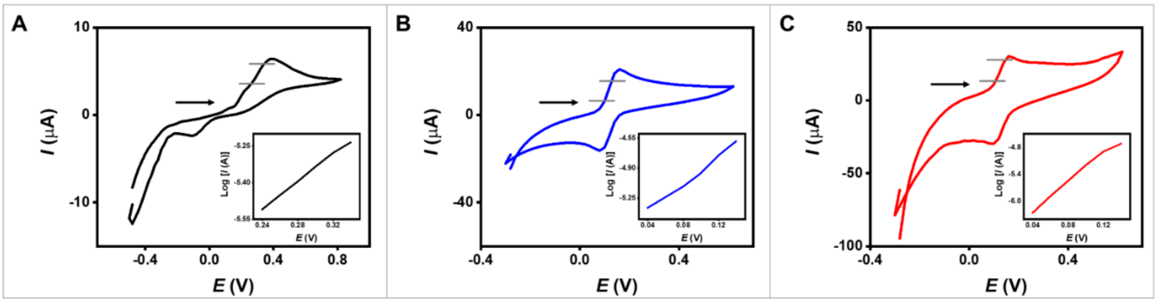

Figure S16. CVs in 500 μM HQ at a scan rate of 12.5 mV s^{-1} of (A) unmodified electrode, (B) $CNT_{2.0}$ electrode, and (C) $PCNT_{2.0}$ electrode. Inset providing the Tafel plot of Log *I* against E.

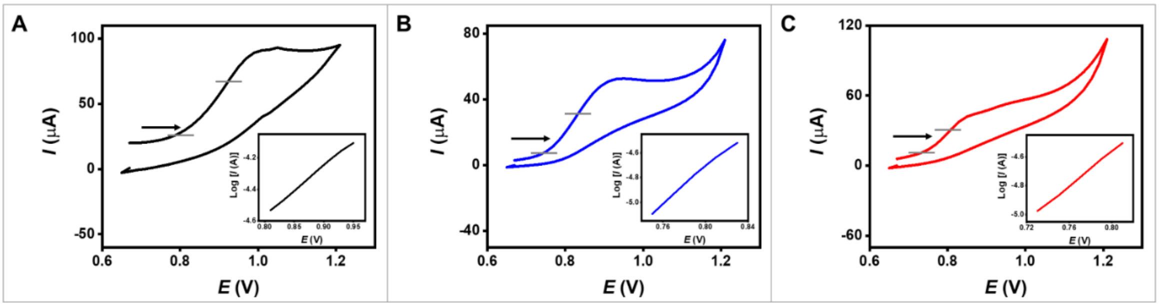

Figure S17. CVs in 2000 μM HQ at a scan rate of 12.5 mV s^{-1} of (A) unmodified electrode, (B) $CNT_{2.0}$ electrode, and (C) $PCNT_{2.0}$ electrode. Insets providing the Tafel plot of Log *I* against E.

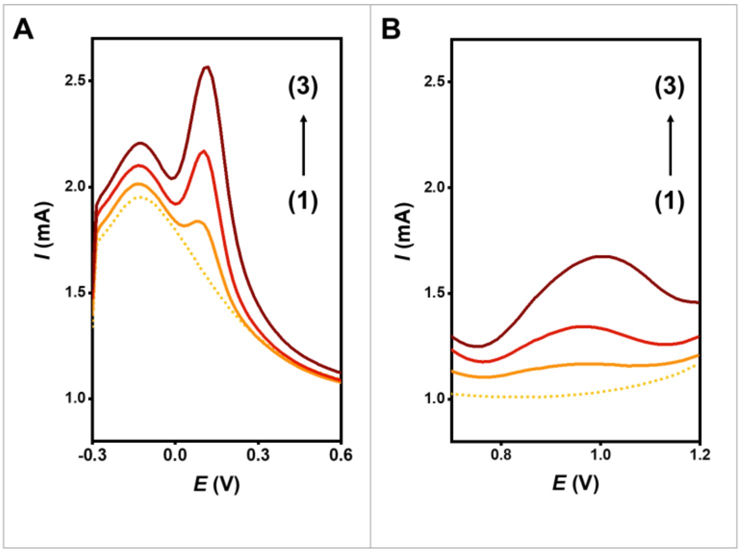

Figure S18. SWVs of $PCNT_{2.0}$ electrode (with a step potential of 15 mV, an amplitude of 125 mV, and a frequency of 50 Hz). The dot plots represent the blank solution whereas the solid lines represent the solution containing various concentrations of (A) HQ: (1-3) 125, 250, and 500 μM. and (B) SA: (1-3) 500, 1000, and 2000 μM.

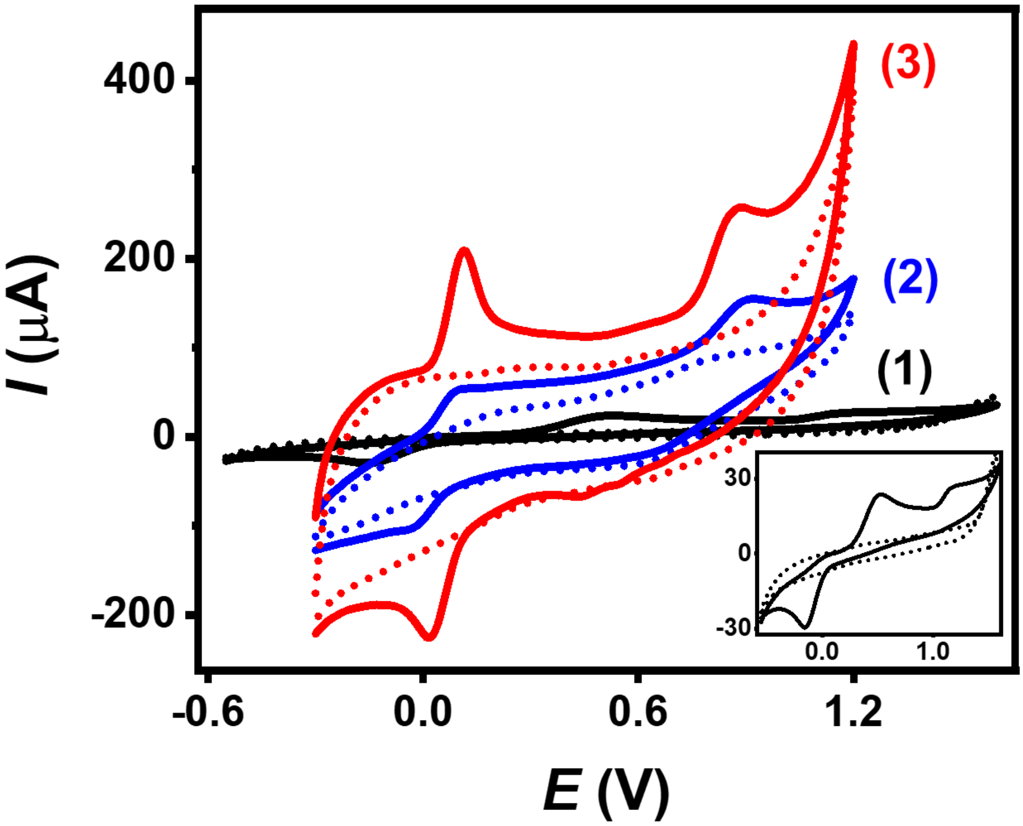

Figure S19. CVs of the (1) unmodified electrode, (2) $CNT_{2.0}$ electrode, and (3) $PCNT_{2.0}$ electrode at a scan rate of 100 mV s^{-1}. The short dot refers to the solution without HQ and SA whereas the solid line refers to the solution containing 500 μM HQ and 500 μM SA.

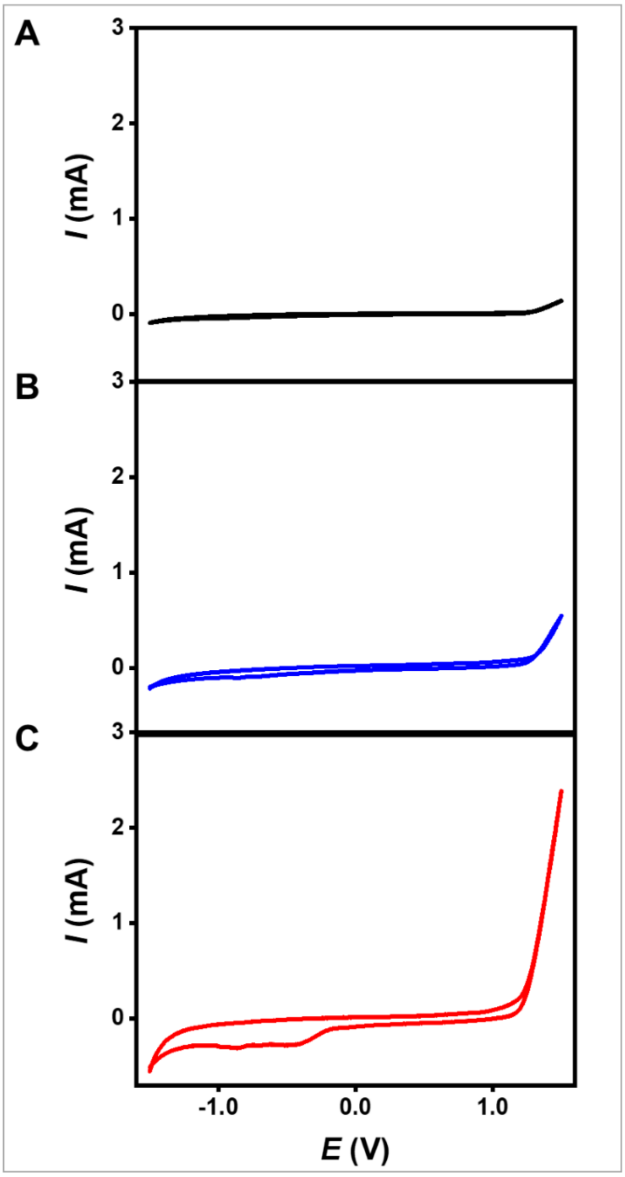

Figure S20. CV responses in 1.0 M Na_2CO_3 of (A) unmodified electrode, (B) CNT electrode, and (C) PCNT electrode with a scan rate of 5 mV s^{-1}.

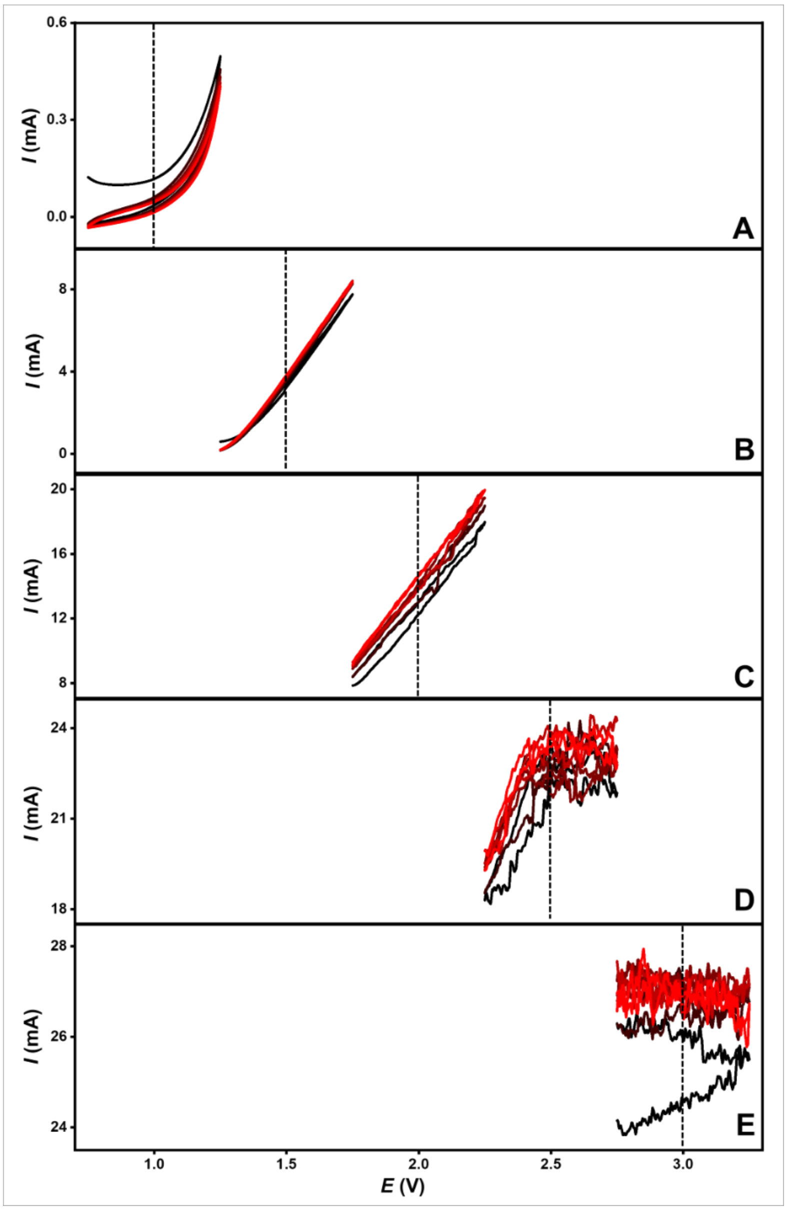

Figure S21. CVs scanned during anodization of the PCNT electrode in 1.0 M Na_2CO_3 at different anodization potential windows, each passing different potential midpoints: (A) 1.0 V (sweeping from 0.75 to 1.25 V), (B) 1.5 V (sweeping from 1.25 to 1.75 V), (C) 2.0 V (sweeping from 1.75 to 2.25 V), (D) 2.5 V (sweeping from 2.25 to 2.75 V), and (E) 3.0 V (sweeping from 2.75 to 3.25 V), with a scan rate of 20 mV s^{-1} for 5 cycles.

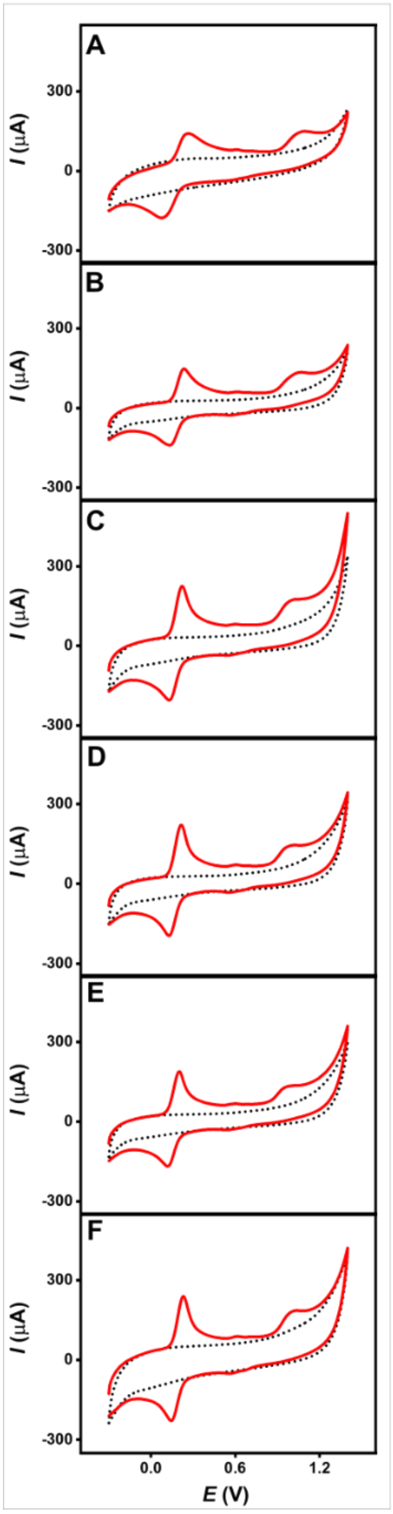

Figure S22. CVs of different electrodes in a mixed solution containing 500 μM HQ and 500 μM SA at a scan rate of 100 mV s^{-1}: (A) PCNT, (B) $PCNT_{1.0}$, (C) $PCNT_{1.5}$, (D) $PCNT_{2.0}$, (E) $PCNT_{2.5}$, and (F) $PCNT_{3.0}$. The black dot represents the electrolyte solution without HQ and SA.

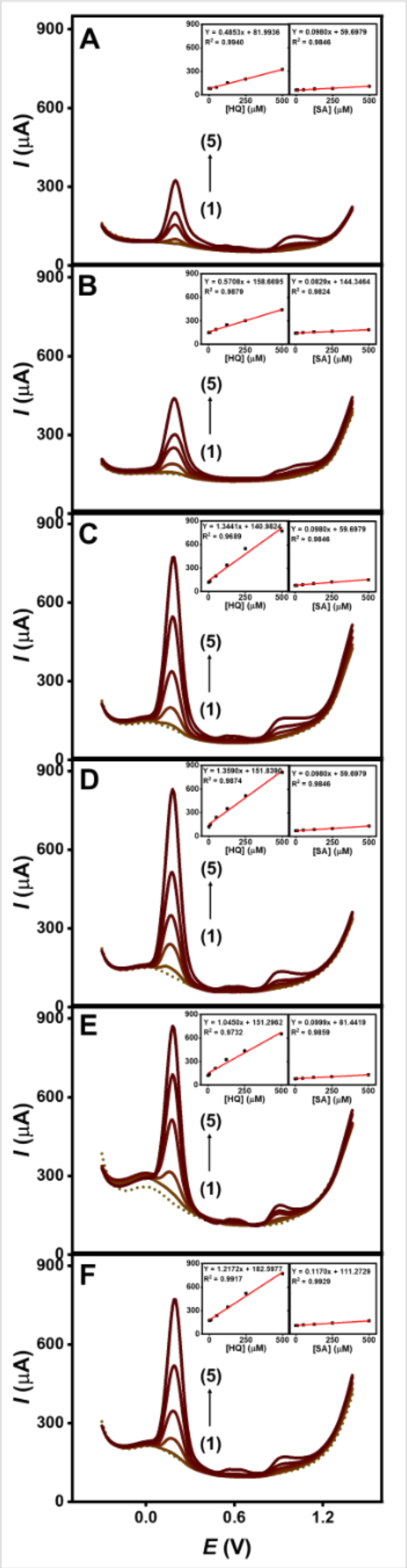

Figure S23. SWVs of electrodes: (A) PCNT, (B) $PCNT_{1.0}$, (C) $PCNT_{1.5}$, (D) $PCNT_{2.0}$, (E) $PCNT_{2.5}$, and (F) $PCNT_{3.0}$ (with a step potential of 10 mV, an amplitude of 75 mV, and a frequency of 5 Hz). The dot plots represent the blank solution whereas the solid lines represent the solution containing various concentrations of HQ and SA: (1-5) 10, 50, 125, 250, and 500 μM. The inset graphs show sensitivity obtained from the slope of the relationship between the SWV peak current and the concentration of HQ or SA.

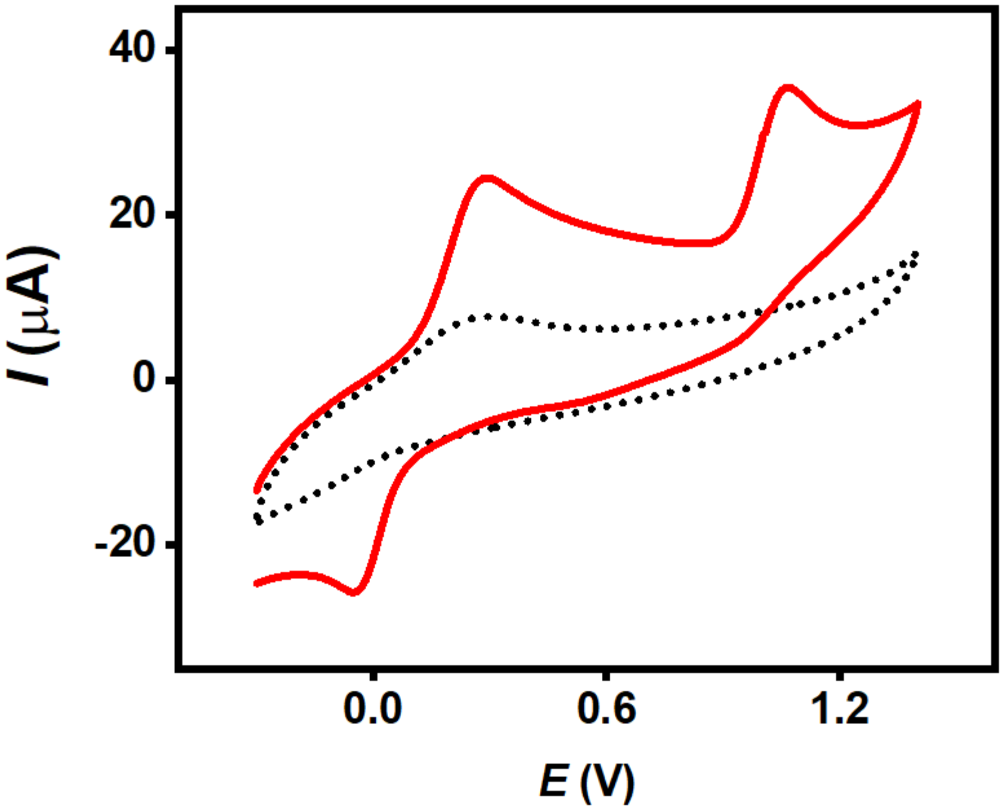

Figure S24. CVs at a scan rate of 100 mV s^{-1} for the unmodified electrode. The solid red line represents a solution with 500 μM HQ and 500 μM SA, while the black dot plot corresponds to the solution without HQ and SA.

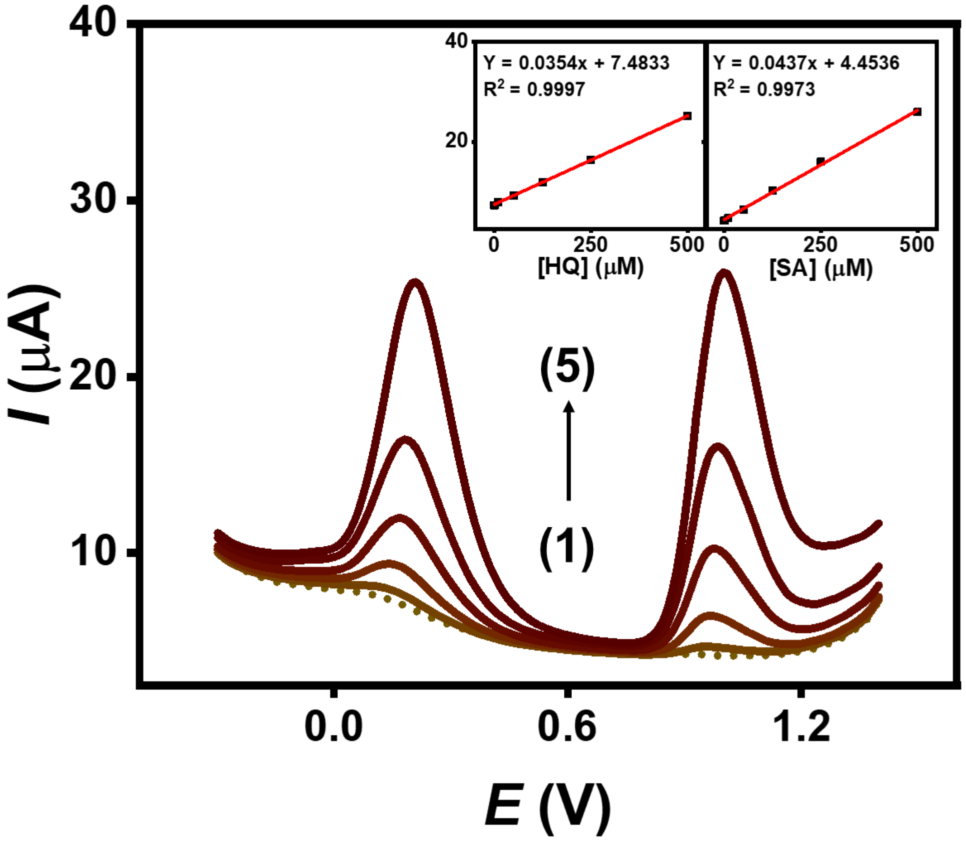

Figure S25. SWVs of the unmodified electrode (with a step potential of 10 mV, an amplitude of 75 mV, and a frequency of 5 Hz). The dot plots represent the blank solution whereas the solid lines represent the solution containing various concentrations of HQ and SA: (1-5) 10, 50, 125, 250, and 500 μM. The inset graphs show sensitivity obtained from the slope of the quantitative relationship between the SWV peak current and the concentration of HQ or SA.

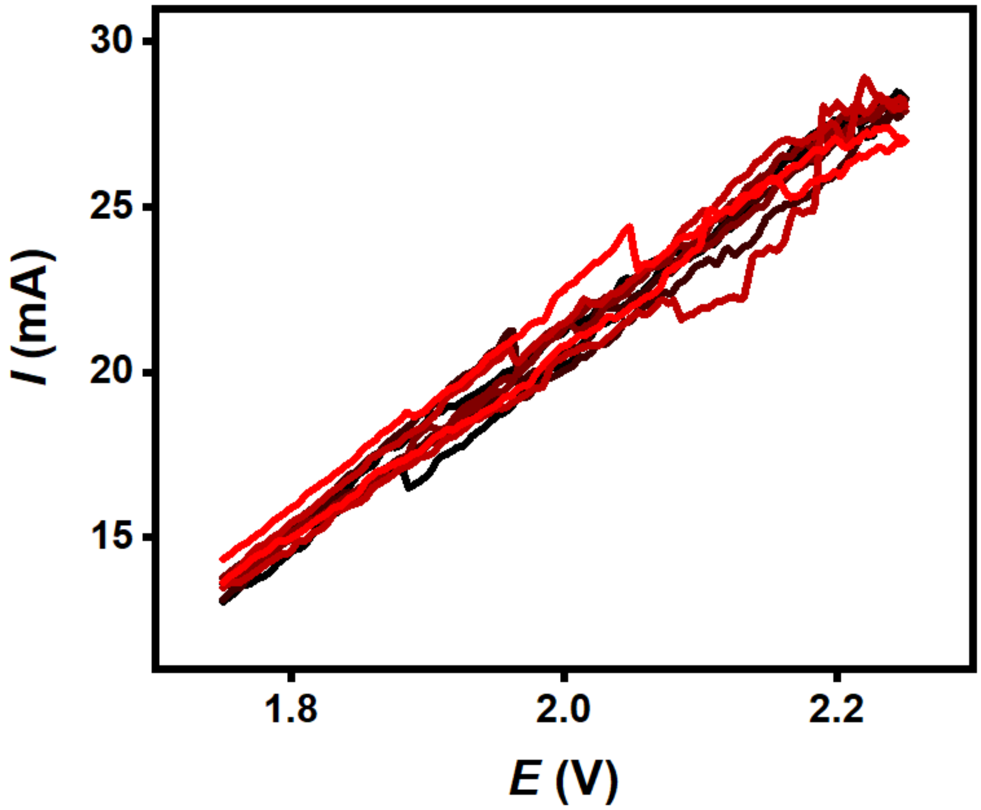

Figure S26. CV response obtained from CNT electrode during anodization in 1.0 M Na_2CO_3 at the midpoint potential window of 2.0 V with a scan rate of 20 mV s^{-1} for 5 cycles.

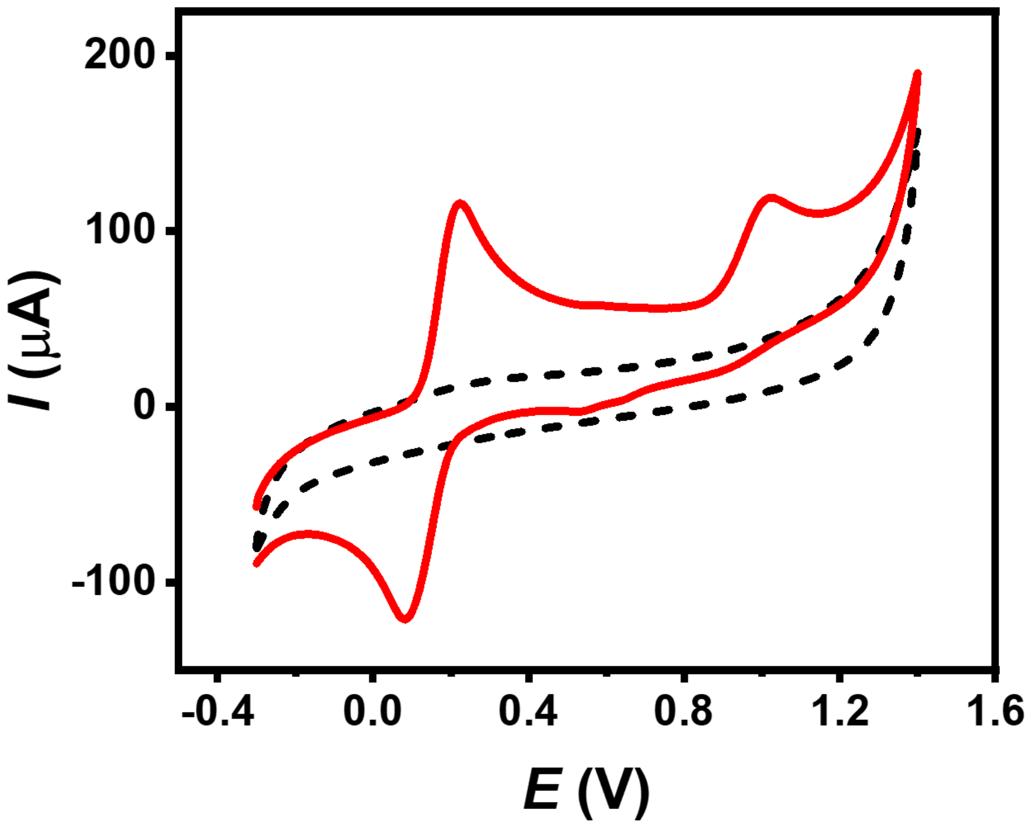

Figure S27. CVs of $CNT_{2.0}$ electrode at a scan rate of 100 mV s^{-1}. The black short dot refers to the solution without HQ and SA whereas the red solid line refers to the solution containing 500 μM HQ and 500 μM SA.

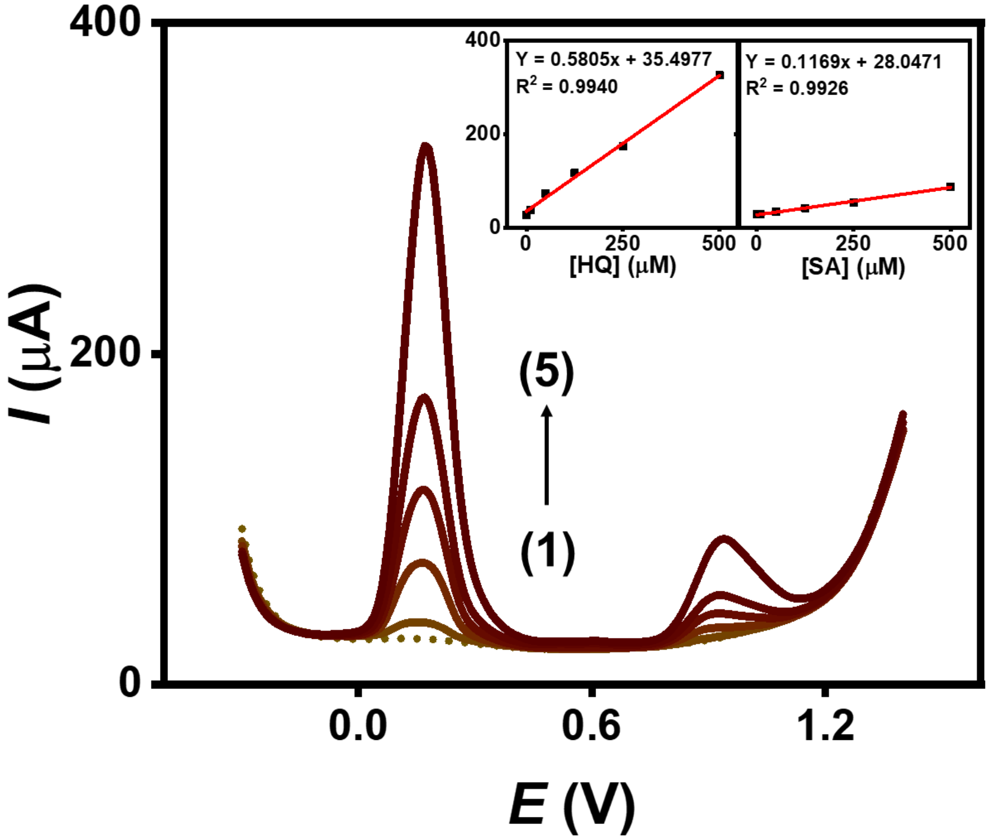

Figure S28. SWVs of the $CNT_{2.0}$ electrode (step potential: 10 mV, amplitude: 75 mV, frequency: 5 Hz). The dotted line represents the solution without HQ and SA, while the solid lines represent the solution with various concentrations of HQ and SA: (1-5) 10, 50, 125, 250, and 500 μM. The inset graphs show sensitivity obtained from the slope of the quantitative relationship between the SWV peak current and the concentration of HQ or SA.

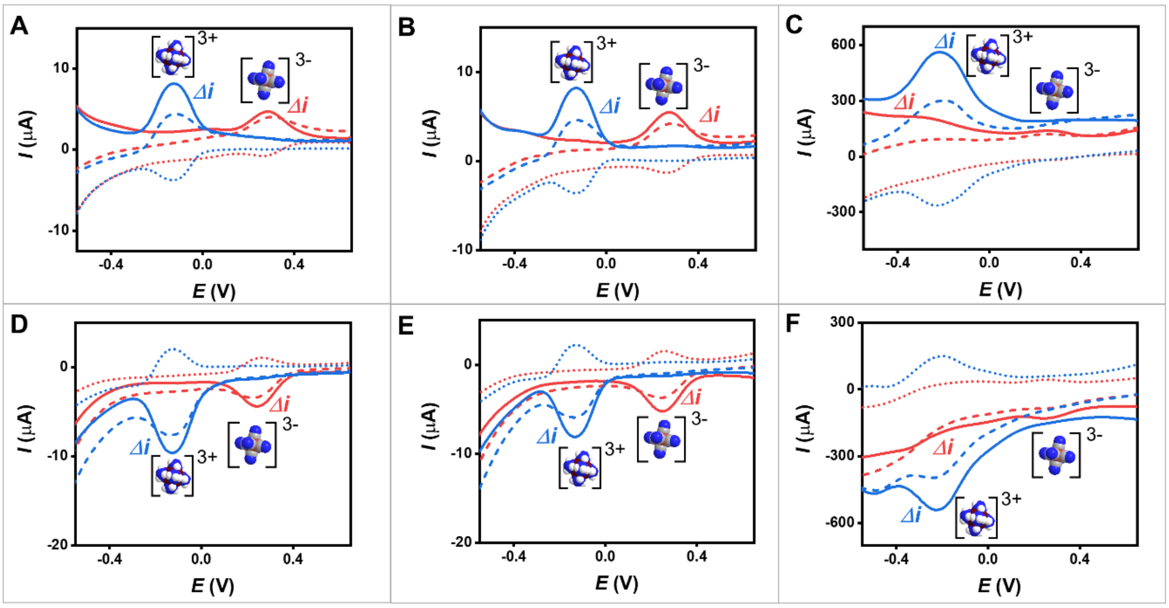

Figure S29. Electroanalysis on different electrodes using SWV with different redox probes, including either 0.1 mM $[Ru(NH_3)_6]^{3+}$ alone (blue plots) or 0.1 mM $[Fe(CN)_6]^{3-}$ alone (red plots) in 0.1 M KCl. (A-C), show SWVs in the oxidation direction for (A) unmodified electrode, (B) CNT electrode, and (C) $PCNT_{2.0}$ electrode. (D-F) show SWVs in the reduction direction for (D) unmodified electrode, (E) CNT electrode, and (F) $PCNT_{2.0}$ electrode. The electroanalytical responses include the net current (Δi, solid lines), the forward current (i_f, dashed plots), and the backward current (i_b, dotted plots).

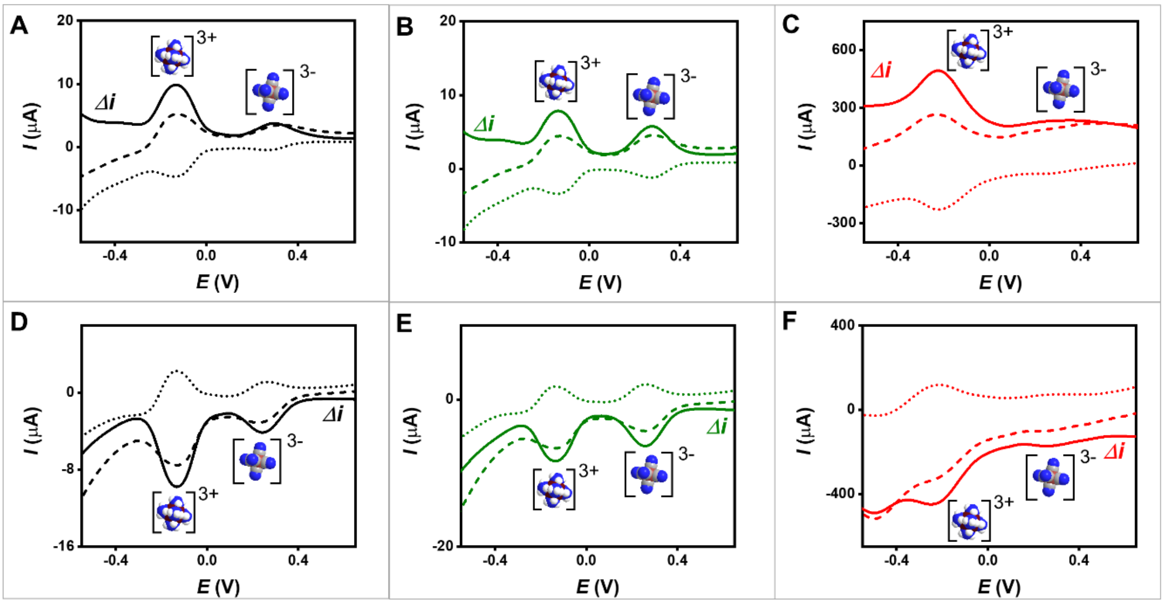

Figure S30. Electroanalysis on different electrodes using SWV with different redox probes, including 0.1 mM $[Ru(NH_3)_6]^{3+}$ and 0.1 mM $[Fe(CN)_6]^{3-}$ in 0.1 M KCl. Panels (A-C) show SWVs in the oxidation direction for (A) unmodified electrode, (B) CNT electrode, and (C) $PCNT_{2.0}$ electrode. Panels (D-F) show SWVs in the reduction direction for (D) unmodified electrode, (E) CNT electrode, and (F) $PCNT_{2.0}$ electrode. The electroanalytical responses include the net current (Δi, solid lines), the forward current (i_f, dashed plots), and the backward current (i_b, dotted plots).

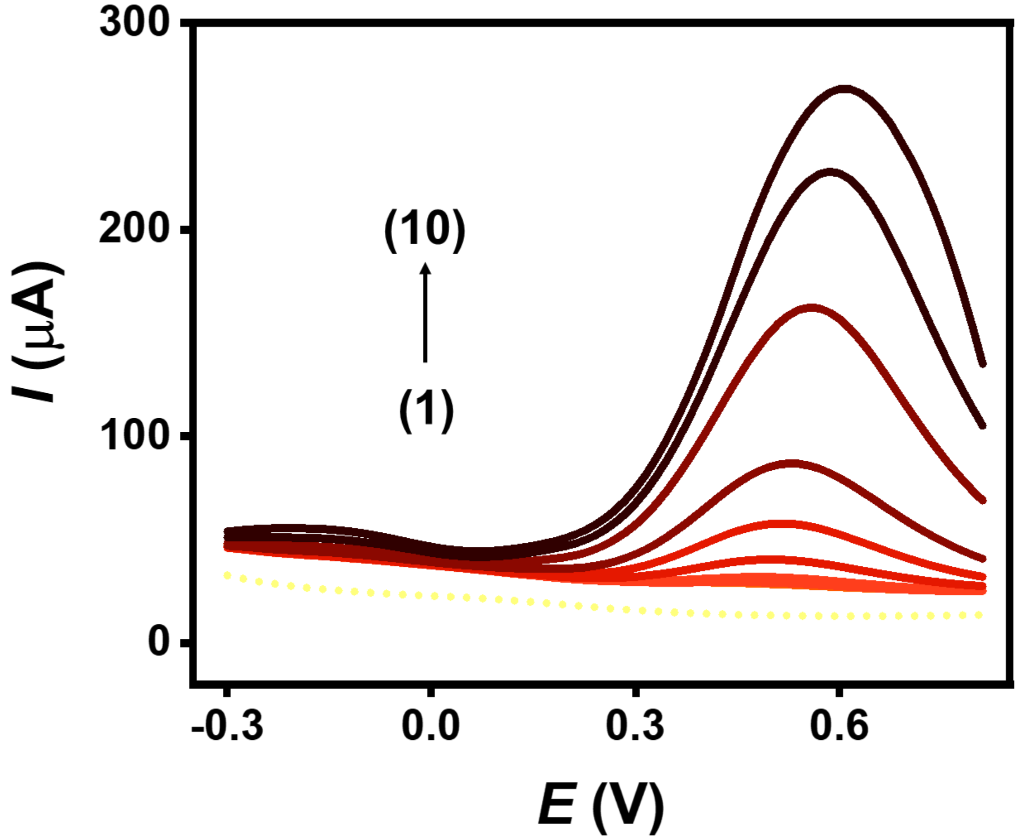

Figure S31. SWVs of unmodified electrode (with a step potential of 15 mV, an amplitude of 125 mV, and a frequency of 15 Hz) in HQ with various concentrations: (1-10) 0, 10, 25, 50, 125, 250, 500, 1000, 1500 and 2000 μM.

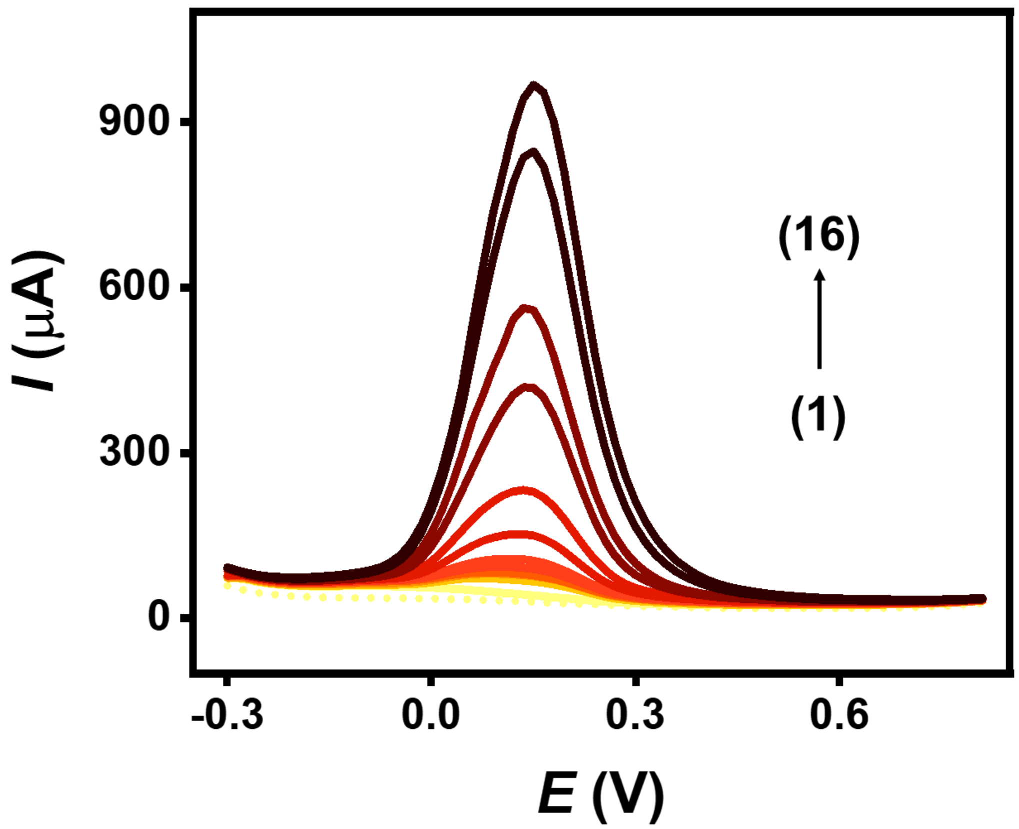

Figure S32. SWVs of $CNT_{2.0}$ electrode (with a step potential of 15 mV, an amplitude of 125 mV, and a frequency of 15 Hz) in HQ with various concentrations: (1-15) 0, 0.1, 0.5, 1, 2.5, 3.75, 5, 10, 25, 50, 125, 250, 500, 1000, 1500 and 2000 μM.

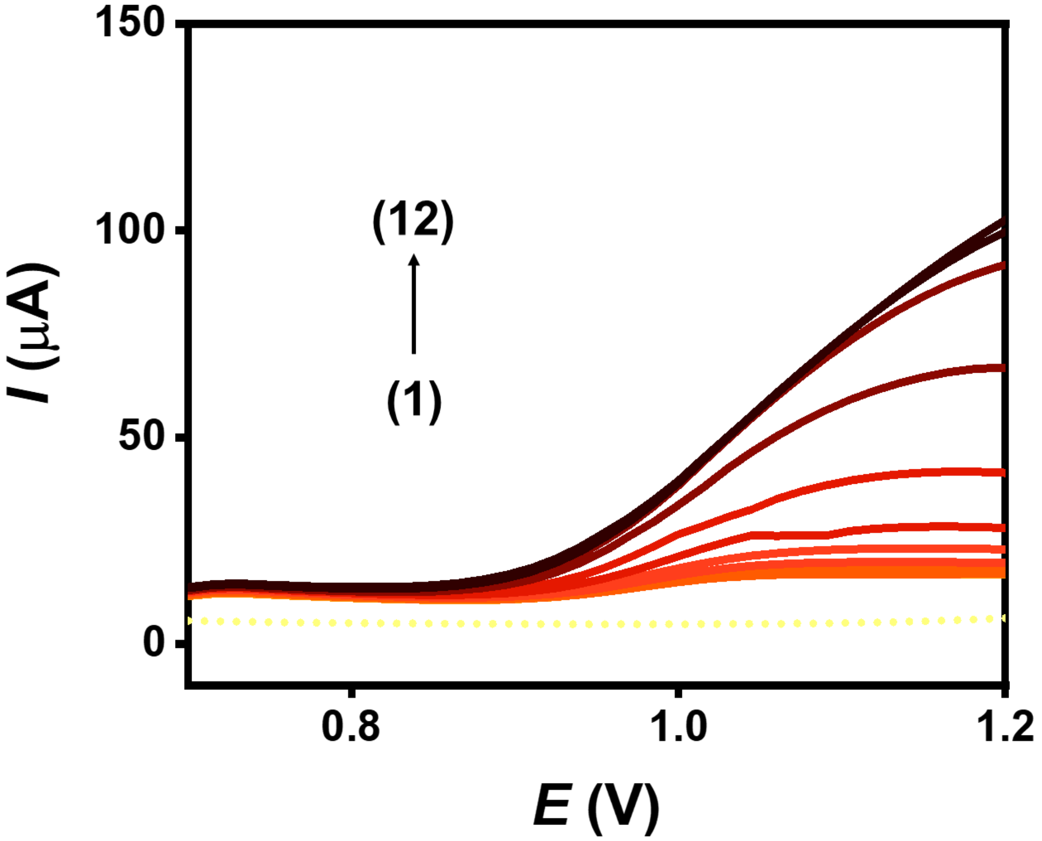

Figure S33. SWVs of unmodified electrode (with a step potential of 15 mV, an amplitude of 125 mV, and a frequency of 15 Hz) in SA with various concentrations: (1-12) 0, 3.75, 5, 10, 25, 50, 125, 250, 500, 1000, 1500 and 2000 μM.

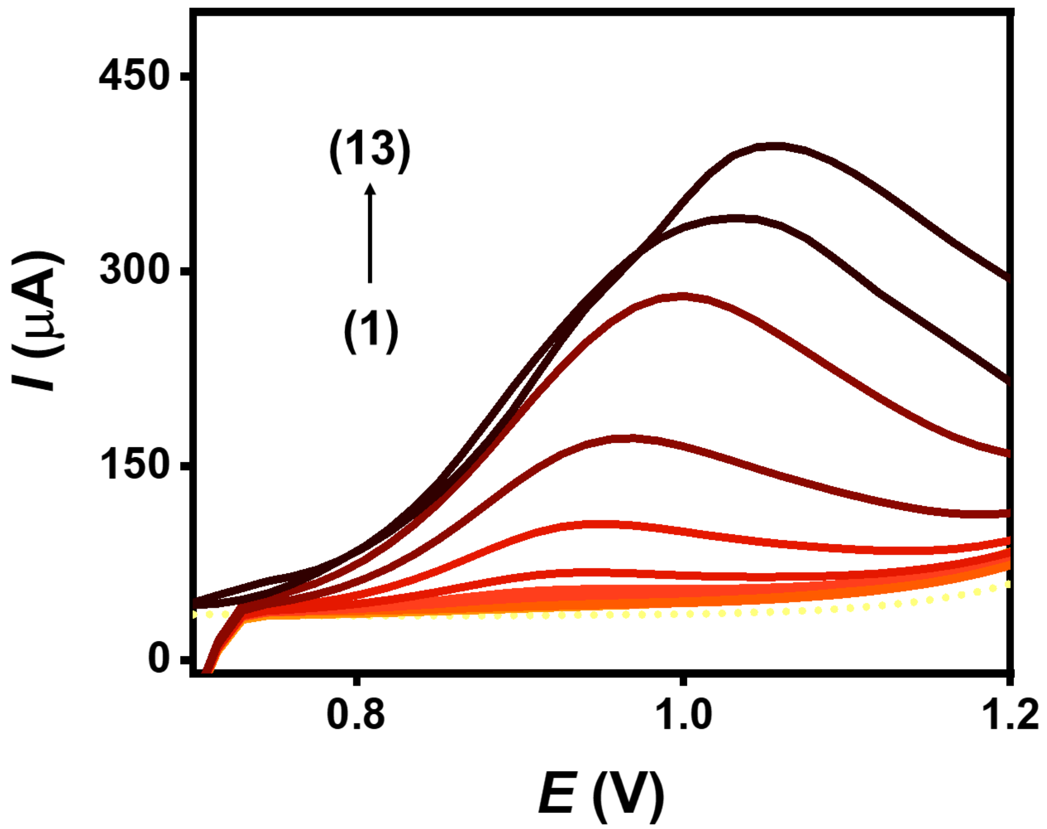

Figure S34. SWVs of $CNT_{2.0}$ electrode (with a step potential of 15 mV, an amplitude of 125 mV, and a frequency of 15 Hz) in SA with various concentrations: (1-12) 0, 2.5, 3.75, 5, 10, 25, 50, 125, 250, 500, 1000, 1500 and 2000 μM.

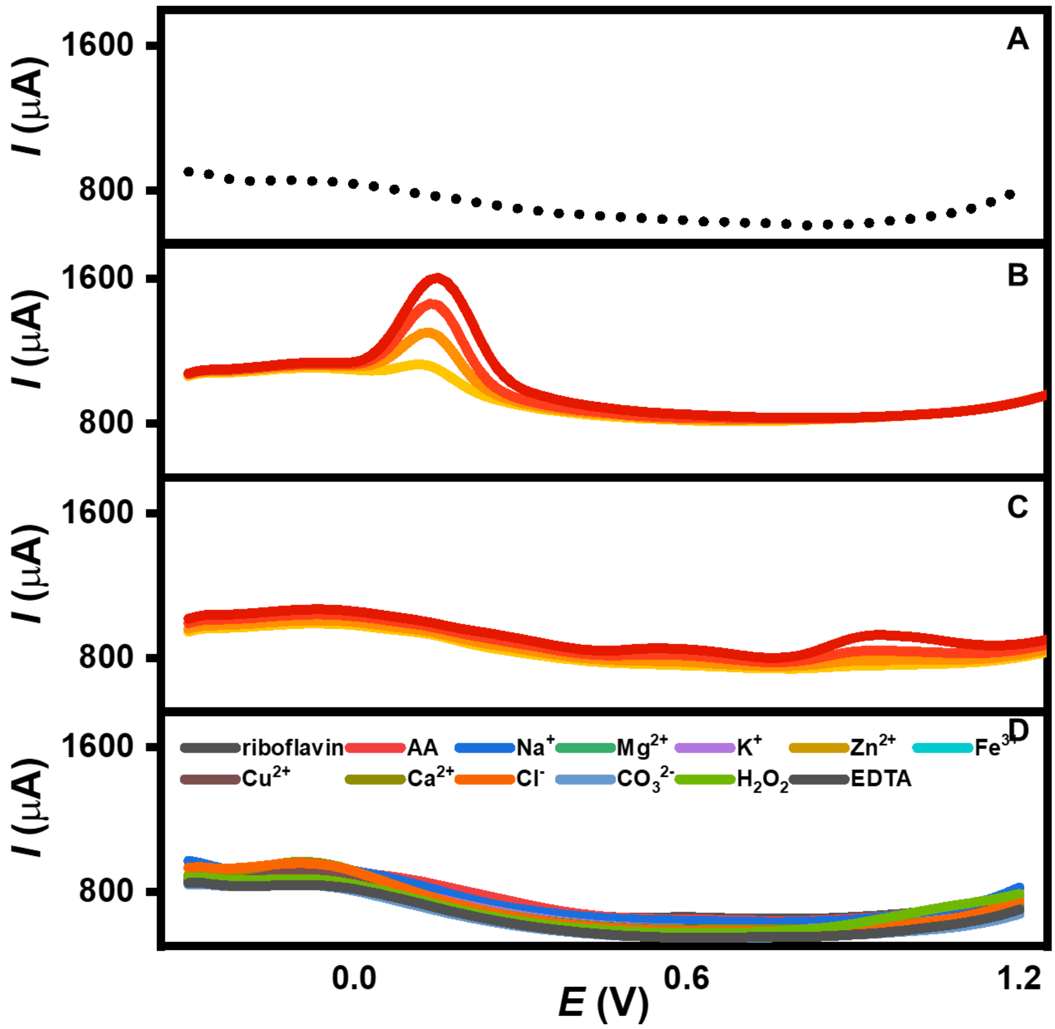

Figure S35. Interference study of $PCNT_{2.0}$ electrode. SWV curves were obtained in (A) 0.1 M PBS (pH 7.0) (B) HQ (100 μM, 250 μM, 500 μM, 1000 μM) (C) SA (100 μM, 250 μM, 500 μM, 1000 μM) (D) potential interferent additives: 1000 μM riboflavin, AA, Na^{+}, Mg^{2+}, K^{+}, Zn^{2+}, Fe^{3+}, Cu^{2+}, Ca^{2+}, Cl^{-}, CO_3^{2-}, H_2O_2, and ethylenediaminetetraacetic acid (EDTA).

2. Supporting tables

Table S1. Electrochemical sensors using various materials for different properties

Electrodes	Flexible	Porous structure	Electrochemical surface engineering	Screen-printing technique	Systematic property tuning through selective interaction	References
Au-CuNPs@PSi/SPCE	✗	✓	✓	✗	✗	1
AuND	✓	✓	✓	✗	✗	2
AuNPs-MPS	✗	✓	✗	✗	✗	3
AuNW	✓	✓	✗	✗	✗	4
CuNPs/PS/SPCE	✗	✓	✗	✗	✗	5
$FeWO_4/SnO_2$/Nafion/GCE	✗	✓	✗	✗	✗	6
GNPs/MWCPE	✗	✓	✓	✗	✗	7
GOD/Pt-LEPG electrode	✓	✓	✓	✗	✗	8
LTOF-800/GCE	✗	✓	✗	✗	✗	9
NOCCs	✓	✗	✗	✗	✓	10
PEDOT:Nafion-CF	✗	✗	✓	✗	✓	11
SMCs	✗	✓	✓	✗	✓	12
Pd@PSi-PPy-C/GCE	✗	✓	✗	✗	✗	13
PNR/P-Gr/GCE	✗	✓	✗	✗	✗	14
rGO/AuNP/MnO_2/SPCE	✓	✓	✓	✗	✗	15
RGO-PDA-cMWCNT/GCE	✗	✓	✗	✗	✗	16
SDAIPC-X/GCE	✗	✓	✗	✗	✗	17
SPEs	✗	✗	✗	✓	✗	18
ZC/GCE	✗	✓	✗	✗	✗	19
Printed flexible CNT-based electrode ($PCNT_{2.0}$)	✓	✓	✓	✓	✓	This work

Abbreviations: materials *Au-CuNPs@PSi/SPCE* Bimetallic nanostructure of gold-copper on the surface of porous silicon/screen-printed carbon electrode, *AuND* Gold nano dendrite, *AuNPs-MPS* Gold nanoparticles mesoporous silica/carbon paste electrode, *AuNW* Gold nanowire sponge, *CuNPs/PS/SPCE* Copper nanoparticles-porous silicon/screen-printed carbon electrode, *$FeWO_4/SnO_2$/Nafion/GCE* Iron tungstate doped tin oxide nanocomposite/nafion/glassy carbon electrode, *GNPs/MWCPE* Gold nanoparticles/multi-walled carbon nanotube/carbon paste electrode, *GOD/Pt-LEPG electrode* Glucose oxidase/Pt-nanoparticles-polyimide-laser-engraved porous graphene electrode, *LTOF-800/GCE* La-Ti Organic Frameworks-800/glassy carbon electrode, *NOCCs* Nitrogen/oxygen-codoped carbon cloths, *PEDOT:Nafion-CF* poly(3,4-ethylenedioxythiophene):Nafion-coated carbon fiber electrodes, *SMCs* highly ordered and vertically oriented silica mesochannels, *Pd@PSi-PPy-C/GCE* Palladium nanoparticles deposited in a porous silicon-polypyrrole-carbon black nanocomposite/glassy carbon electrode, *PNR/P-Gr/GCE* Poly(neutral red)/porous graphene/glassy carbon electrode, *rGO/AuNP/MnO_2/SPCE* Reduced

graphene oxide/gold nanoparticles/manganese dioxide nanocomposite/screen-printed carbon electrode, *RGO-PDA-cMWCNT/GCE* Reduced graphene oxide-polydopamine-carboxylated multi-walled carbon nanotube/glassy carbon electrode, *SDAIPC-X/GCE* Sodium D-isoascorbic acid interconnected porous carbon-x (x represents the carbonizationtemperature)/glassy carbon electrode, *SPEs* Screen-printed carbon electrodes, and *ZC/GCE* Zeolite imidazole ester framework-8/glassy carbon electrode.

Table S2. The EIS results showing parameter values obtained from equivalent circuit settings using different printed electrodes, including the unmodified electrode, CNT electrode, and $PCNT_{2.0}$ electrode.

**Materials**	**Elements**				
	**R_s (Ω)**	**R_{ct} (Ω)**	**CPE_{dl} Y_0 (S . s^N) (nMho)**	**CPE_{dl} N**	**W_D Y_0 (S . $\sqrt{s}$) (μMho)**
Unmodified electrode	52.3	124	0.452	1.1	90.0
CNT electrode	38.2	96.4	0.559	1.1	300.0
$PCNT_{2.0}$ electrode	26.4	49.4	1.290	1.1	1000.0

Table S3. Analytical performances of reported work and our porous nanocomposite electrode for the HQ detection.

Electrodes	Electrochemical techniques	Flexible	Printable	Wearable	Porous	Range (μM)	LOD (μM)	Ref
Activated carbon-CPE	DPV	✗	✗	✗	✓	0.05-1000	0.036	20
aGO1/SPCE	DPV	✗	✗	✗	✗	1-312	0.270	21
AuNPs/EGPE	DPV	✓	✗	✗	✗	0.07-100	0.041	22
Au-Sn alloy/integrated chip	DPV	✗	✗	✗	✓	0.2-100	0.083	23
BGQDs/ZnO NR-CFME	DPV	✓	✗	✗	✗	0.1-100	0.030	24
GN–CN–MT/GCE	DPV	✗	✗	✗	✗	0.1-600	0.040	25
GO–mesoporous MnO_2/GCE	DPV	✗	✗	✗	✓	0.01-1	0.007	26
Graphene/GCE	DPV	✗	✗	✗	✗	1-80	0.800	27
Gr-COOH/GCE	AdASV	✗	✗	✗	✗	0.1-40	0.040	28
LTOF-800/GCE	DPV	✗	✗	✗	✓	0.4-20	0.039	9
N,S-FeNi3/C NPs	SWV	✗	✗	✗	✓	0.1-100	0.048	29
Poly(Phenol red)MPs/GCE	AdASV	✗	✗	✗	✓	0.091-908	0.025	30
Porous reduced graphene oxide/GCE	DPV	✗	✗	✗	✓	5-90	0.080	31
TFPPy-BD-COF/MWCNT/GCE	DPV	✗	✗	✗	✗	4-450	0.380	32
TpBD-COF Modified CPE	DPV	✗	✗	✗	✓	1-2000	0.310	33
$PCNT_{2.0}$	SWV	✓	✓	✓	✓	0.1-2000	0.046	This work

Abbreviations: materials: *activated carbon-CPE* Activated carbon-carbon paste electrodes, *aGO1/SPCE* Activated graphene oxide modifies screen printed carbon electrode, *AuNPs/EGPE* Gold nanoparticles electrodeposition to exfoliated graphite paper electrode, *Au-Sn alloy/integrated chip*

Gold-Tin alloy/integrated chip electrode, *BGQDs/ZnO NR-CFME* Boron-doped graphene quantum dots/ZnO nanorod-carbon fiber microelectrodes, *GN–CN–MT/GCE* Graphene nanosheets and carbon, *GO–mesoporous MnO_2/GCE* Graphene oxide-mesoporous MnO_2 modified glassy carbon electrodes, *Graphene/GCE* Graphene modified glassy carbon electrodes, *Gr-COOH/GCE* Carboxylic acid-functionalized graphene-modified glassy carbon electrode, *LTOF-800/GCE* La-Ti organic frameworks-800°C modified glassy carbon electrodes, *N,S-$FeNi_3$/C NPs* Nitrogen and sulfur co-doped Fe–Ni alloy (N,S-FeNi3/C) nanoparticles, *Poly(Phenol red)MPs/GCE* Poly(phenol red) micro-structure modified glassy carbon electrodes, *Porous reduced graphene oxide/GCE* Porous reduced graphene oxide modified glassy carbon electrodes, *TFPPy-BD-COF/MWCNT/GCE* 1, 3, 6, 8-tetrakis(4-formylphenyl) pyrene and 4,4′-diphenyldiamine-Covalent organic frameworks/multi-walled carbon, TpBD-COF Modified CPE Triformylphloroglucinol-Benzidine Covalent Organic Framework modified carbon paste electrode, and *$PCNT_{2.0}$* Porous nanocomposite electrode.

Table S4. Analytical performances of reported work and our porous nanocomposite electrode for the SA detection.

**Electrodes**	**Electrochemical techniques**	**Flexible**	**Printable**	**Wearable**	**Porous**	**Range (μM)**	**LOD (μM)**	**Ref**
CB-MWNT-Nafion/Fc/CB-MWNT/GC	DPV	✗	✗	✗	✗	25-1000	3.30	[34]
CFE	DPV	✗	✗	✗	✓	2-3000	1.68	[35]
Cu-MOFs-CB-nafion/SPE	DPV	✗	✗	✗	✓	100-900	12.50	[36]
GO-GCE	DPV	✗	✗	✗	✗	25-2250	10.00	[37]
Graphene hydrogel/GCE	LSV	✗	✗	✗	✓	4-200	2.80	[38]
SPCE	DPV	✗	✗	✗	✗	1-200	1.60	[39]
SPE	SWV	✗	✓	✗	✓	16-300	5.60	[40]
SPGrE	CV	✗	✗	✗	✗	10-150	8.70	[41]
$PCNT_{2.0}$	SWV	✓	✓	✓	✓	10-2000	8.51	This work

Abbreviations: materials: *CB-MWNT-Nafion/Fc/CB-MWNT/GC* Carbon black-multi wall carbon nanotubes-nafion/ferrocene/carbon black-multi wall carbon nanotubes/glassy carbon electrode, *CFE* Carbon-fiber electrode, *Cu-MOFs-CB-nafion/SPE* Cu metal-organic frameworks-carbon black-nafion/screen printed electrode, *GO-GCE* Graphene-oxide-modified glassy carbon electrode, *Graphene hydrogel/GCE* Graphene hydrogel/glassy carbon electrode, *SPCE* Screen-printed carbon electrode, *SPE* Screen-printed electrode, *SPGrE* Graphene-oxide-modified glassy carbon electrode, and $PCNT_{2.0}$ Porous nanocomposite electrode.

3. Supporting notes

3.1. Note S1: Randles-Sevcik relationship

The electrochemically active surface area was derived from background-subtracted CV peak currents at 25 °C based on the Supporting Equation S1

$$i_p = (2.69 \times 10^5) n^{\frac{3}{2}} A D^{\frac{1}{2}} v^{\frac{1}{2}} C \qquad \text{(Equation S1)}$$

where i_p is peak current (A), n is number of electrons transferred in the redox event, A is electrochemically active surface area (cm^2), D is diffusion coefficient ($cm^2\ s^{-1}$), v is scan rate ($V\ s^{-1}$), C is concentration ($mol\ cm^{-3}$)[42]. For Ruthenium (III) hexammine chloride in 0.1 M KCl, the D is found to be $8.50\times10^{-6}\ cm^2\ s^{-1}$[43].

3.2. Note S2: Apparent capacitance

Apparent capacitance (C_{app}) of electrodes was evaluated according to CVs data and calculated based on Supporting Equation S2

$$Capp = \frac{I}{v} \qquad \text{(Equation S2)}$$

Where I is current (A), and v is scan rate ($V\ s^{-1}$)

3.3. Note S3: Areal capacitance

Areal capacitance (C_{areal}) of the electrodes was calculated based on Supporting Equation S3

$$C_{areal} = \left(\frac{1}{2\times(V_2-V_1)\times v}\int_{V_1}^{V_2} |I\ (V)|dV\right)\frac{1}{A} = \frac{C}{A} \qquad \text{(Equation S3)}$$

where $I(V)$ is the instantaneous current in each potential (A), V_2 and V_1 are the upper and lower potential of the chosen potential window (V), v is the scan rate ($V\ s^{-1}$), and A is the geometrical area of the electrode which is $0.15\ cm^2$.

3.4. Note S4: The number of electrons involved in the reaction

The determination of the number of electrons involved in an electrode reaction is applied by using Supporting Equation S4.

$$E_p = E_0 + \frac{2.303RT}{\propto nF} log \frac{RTk^0}{\propto nF} + \frac{2.303RT}{\propto nF} \log v \qquad \text{(Equation S4)}$$

Where E_0 is formal potential, v is scan rate ($V\ s^{-1}$), F the faraday constant ($96485\ C\ mol^{-1}$), T the temperature (298 K), k^0 is heterogeneous rate constant, and n is the number of electrons[44].

3.5. **Note S5: Formal potentials**

The formal potentials ($E^{0'}$) were calculated based on Supporting Equation S5

$$E^{0'} = E_{pa} - \alpha(E_{pa} - E_{pc}) \quad \text{(Equation S5)}$$

where $\alpha = 0.5$, E_{pa} is anodic peak potential (V), and E_{pc} is cathodic peak potential (V).

3.6. **Note S6: Electron transfer constant**

The electron transfer constant (k^0) of electrodes was calculated based on Supporting Equation S6 and S7.

$$\psi = k^0[\pi DnF/RT]^{-\frac{1}{2}} \quad \text{(Equation S6)}$$

$$\psi = (-0.6288 + 0.0021\Delta E_p)/(1 - 0.017\Delta E_p) \quad \text{(Equation S7)}$$

where D is the diffusion coefficient of the electroactive species ($m^2 s^{-1}$), n is the number of electrons transferred in the electrochemical reaction, F is the Faraday constant ($C mol^{-1}$), R is the molar gas constant ($J mol^{-1} K^{-1}$), T is the absolute temperature (K), and ΔE_p is the potential gap between anodic and cathodic peaks (mV)[45].

3.7. **Note S7: Catalytic rate constant**

The catalytic rate constant (K_{cat}) for the oxidation of hydroquinone (HQ) and salicylic acid (SA) on electrodes was evaluated based on Supporting Equation S8.

$$\frac{I_{cat}}{I_L} = (\pi K_{cat} Ct)^{\frac{1}{2}} \quad \text{(Equation S8)}$$

Where I_{cat} represents the current of electrode with the presence of analyte such as HQ or SA (A), I_L is in the absence of analyte (A), C is the concentration (M), and t is the time (s)[46,47].

3.8. **Note S8: The *b*-value**

The charge storage mechanisms can be determined by the *b*-value[48], which can be derived from the power law equation (Supporting Equation S9):

$$i = av^b \quad \text{(Equation S9)}$$

Where i is the current (A), v is the scan rate, a and b are coefficients. The *b*-values can be obtained from the slope of the plot between peak currents and scan rates in a log plot.

4. Supporting experimental procedures

4.1. Chemicals and reagents

Multi-walled carbon nanotubes (CNT) with 95% purity, a diameter ranging from 5 to 15 nm, and length between 10 and 30 μm were procured from Louyang Advanced Material Co., Ltd, China. Screen printing conductive ink was obtained from Ceres, Guangzhou Print Area Technology, China. Sodium hydrogen carbonate ($NaHCO_3$), sodium chloride (NaCl), potassium chloride (KCl), and hydrogen peroxide (30%) were from Merck, Germany. Zinc sulfate ($ZnSO_4$) was from Sigma Diagnostics. Potassium hexacyanoferrate(III) ($K_3[Fe(CN)_6]$), SA, hexaammineruthenium(III) chloride ($[Ru(NH_3)_6]Cl_3$), (-)-riboflavin (vitamin B2), L-ascorbic acid, HQ, glutaraldehyde solution (25%), mineral oil, polyvinyl butyral (PVB), Glucose Oxidase from *Aspergillus niger* (Type VII, lyophilized powder, ≥100,000 units g^{-1} solid), and graphite (particle size <20 μm) were purchased from Sigma-Aldrich Pte. Ltd, Singapore. Sodium carbonate (Na_2CO_3), calcium chloride dihydrate ($CaCl_2.2H_2O$), disodium hydrogen phosphate ($Na_2HPO_4 \cdot 12H_2O$), sodium phosphate dibasic anhydrous ($NaH_2PO_4 \cdot 2H_2O$), and sodium chloride (NaCl) were from Ajax Finechem Pty Ltd, New Zealand. Copper (II) sulfate pentahydrate ($Cu_2SO_4.5H_2O$) was from APS Finechem, Australia. Iron(III) chloride hexahydrate ($FeCl_3.6H_2O$) was from Loba Chemie, India. Magnesium Chloride ($MgCl_2$) was purchased from Carlo Erba Reagents GmbH, Germany. Ethylenediaminetetraacetic acid (EDTA) was from Fluka, Germany. Styrene ethylene butylene styrene block copolymer (SEBS) was from Kraton, USA. Hydrochloric acid was from RCI Labscan, Thailand. Tetrahydrofuran (THF) was obtained from Fisher Scientific, USA. Toluene was purchased from Guangdong Chemical, China. Polyvinyl alcohol (PVA) was from Chem-Supply Pty Ltd, Australia. Polyethylene terephthalate (PET) sheet with a thickness of 0.18 mm was from C.A.P. Intertrade, Thailand. Potassium hydrogen phthalate was purchased from Anapure, New Zealand. Ultrapure water (18.2 MΩ cm) from a Milli Q Merck system (Germany) was used to prepare all solutions.

4.2. Preparation of CNT electrode ink and CNT electrode

70.0 mg of CNTs, 50.0 mg of graphite, and 7.0 g of pristine conductive ink were mixed. This mixture was then supplemented with 200 μL of 1% SEBS in toluene and 2450 μL of toluene. The resulting dispersion underwent sonication for 30 minutes. Subsequently, the dispersion was homogenized using a high-speed mixer at 1790 rpm for 30 minutes to produce the CNT-modified ink. The CNT-modified electrode, hereafter referred to as the "CNT electrode," was fabricated by screen-printing the electrode ink onto a flexible substrate. The screen-printed CNT electrode was dried in a conventional hot-air oven at 70 °C for 20 minutes. The working area of the screen-printed CNT electrode was defined as 0.3 cm by 0.5 cm.

4.3. Preparation of PCNT electrode ink and PCNT electrode

14.0 mg of CNTs, 7.0 g of screen-printing conductive ink, and 4.2 g of ground sodium hydrogen carbonate were combined. Subsequently, 4200 μL of toluene was added, followed by sonication for 30 minutes to produce the CNT-modified ink with loaded porogen material. The resulting ink was then screen-printed onto the CNT electrode and dried in a conventional hot-air oven at 70°C for 20 minutes. Finally, the screen-printed electrode obtained was treated with 0.2 M HCl for 15 minutes. Following this acid treatment, the screen-printed electrode was thoroughly washed with water to yield the porous flexible electrode, abbreviated as "PCNT electrode".

4.4. Anodization of PCNT electrodes

Each PCNT electrode was subjected to anodization in a 1.0 M Na_2CO_3 aqueous solution using cyclic voltammetry at a scan rate of 20 mV s^{-1} for 5 cycles within a specific potential window. Various PCNT electrodes were anodized with different potential windows: 0.75 V to 1.25 V to obtain $PCNT_{1.0}$; 1.25 V to 1.75 V to obtain $PCNT_{1.5}$; 1.75 V to 2.25 V to obtain $PCNT_{2.0}$; 2.25 V to 2.75 V to obtain $PCNT_{2.5}$; and 2.75 V to 3.25 V to obtain $PCNT_{3.0}$; The subscripts represent the midpoint of each potential window.

4.5. *Fabrication of PVA cryogel*

A PVA solution was prepared by dissolving 0.5 g of PVA in 20 mL of water, which was then stirred at a temperature of approximately 90 °C. 10 mL of the PVA solution was mixed with 180 μL of 2.0 M HCl and 1131 μL of 0.5% glutaraldehyde solution before being poured into a mold. The cast solution was then placed in a freezer at –20°C for 12 hours. Subsequently, the PVA cryogel was immersed in water to eliminate any remaining HCl. Finally, the resulting gel was soaked in the electrolyte to create a gel-electrolyte support for on-demand electrochemical detection on a glove fingertip.

4.6. *Preparation of skincare cream sample*

A commercially available skincare product was purchased from a local supermarket in Thailand. 100 mg of the product was mixed with 2000 μL of 0.1 M PBS (pH 7.0). The mixture was sonicated for 30 minutes and then centrifuged at 10,000 rpm for 20 minutes. The supernatant was collected and used to quantify analytes using titration or electrochemical SWV methods.

4.7. *Fabrication of flexible porous electrodes on a wearable glove and its application*

A wearable electrochemical sensor integrated with a glove was fabricated using a screen-printing technique, incorporating three electrodes. First, the glove fingertip was coated with flexible stretchable fabric glue (Aleene, USA) and allowed to set for 2 hours. Next, a silver conductive ink (Ag/AgCl, Sun Chemical Ltd., UK) was screen-printed as the reference electrode. CNT-modified ink was printed as the counter electrode and the first layer of the working electrode, then allowed to dry. Subsequently, CNT-modified ink was printed on the top layer of the working electrode, followed by acid treatment and anodization over a potential range of 1.75 to 2.25 V for five cycles. The printed reference electrode was coated with 100 μL of a reference membrane solution, which was prepared by dissolving 78 mg of PVB and 50 mg of NaCl in 1 mL of methanol [49] and thoroughly mixed using sonication. The coated electrode was then dried at room temperature.

To demonstrate the application of the printed flexible porous electrode on a wearable glove for sample screening, each sample (comprising 95% real sample and 5% mineral oil fortified with different amounts of HQ and/or SA) was wiped onto PVA gel absorbed with 0.1 M PBS (pH 7.0) as electrolyte support. For on-finger screening, real samples were applied directly to the gel surface, covered by a cellulose membrane. The membrane was placed between the sample-coated gel and the electrodes for detection.

4.8. *Titration protocol for determining SA in the skincare sample*

The amount of salicylic acid in a real sample was determined using titration analysis. The standard titration method was adapted to quantify the amount of salicylic acid (SA) present[50]. Briefly, 250 mg

of the sample was mixed with 5500 μL of 0.1 M NaOH in a flask at 70°C for 15 minutes, then allowed to cool to room temperature. Next, 6500 μL of 0.1 M HCl was gradually added to the solution, followed by a few drops of phenolphthalein. The solution was titrated with a standard 0.1 M NaOH until the endpoint was reached. The 0.1 M NaOH solution was standardized with a primary standard 0.1 M potassium hydrogen phthalate using phenolphthalein as the indicator, while the 0.1 M HCl solution was standardized with a primary standard 0.1 M Na_2CO_3 with a few drops of bromocresol green indicator.

4.9. Preparation of $PCNT_{2.0}$-HQ-GOx electrode and glucose biosensor

100 μL of 5 mM HQ in 0.1 M PBS (pH 7.0) was added to the $PCNT_{2.0}$ electrode (geometric area of 0.15 cm^2). After 30 minutes, the electrode was wiped to remove the solution covering it and dried at room temperature for 30 minutes. Subsequently, 20 μL of 10 mg mL^{-1} GOx solution was drop-cast onto the resulting electrode and dried at room temperature for 12 h to obtain the $PCNT_{2.0}$ electrode immobilized with HQ and GOx, abbreviated as "GOx/HQ/$PCNT_{2.0}$ electrode". The electrochemical performance of the glucose biosensor was evaluated using amperometry with an applied potential of 0.4 V in solutions containing various glucose concentrations.

4.10. Electrochemical Studies and Characterizations

The electrochemical analyses were conducted using the bipotentiostat/galvanostat analyzer (Metrohm DropSens, μStat-I 400, Netherlands), operated with DropView software version 8400. Additionally, the electrochemical analyzer (Metrohm Autolab type III FRA 2, Netherlands) was employed, controlled by NOVA software version 2.1.5 for electrochemical impedance spectroscopy investigations. A multimeter (Keysight 34465A) was also utilized, controlled by Keysight BenchVue software version 3.0 for resistance analysis. All electrochemical investigations were performed using a 0.1 M PBS (pH 7.0), unless stated otherwise. The voltage presented in the CVs, SWVs, and amperometric plots corresponds to the potential measured versus the Ag/AgCl reference electrode in 3.0 M KCl. For the wearable electrochemical sensor integrated with a glove, the voltages are referenced to the screen-printed Ag/AgCl electrode. Water contact angle measurements were recorded using a digital camera operated with MRK software and analyzed with ImageJ software version 1.53a. Electrochemical impedance spectroscopy (EIS) was performed with an amplitude of 10 mV. For the unmodified electrode, a DC of 0.05 V was applied, with a frequency range of 1×10^3 – 2×10^6 Hz. For the CNT and $PCNT_{2.0}$ electrodes, a DC of 0.05 V was applied, with a frequency range of 1×10^2 – 2×10^6 Hz. Surface morphology of samples was observed by scanning electron microscope (SEM, Thermo Fisher Scientific Quanta 400, Czech Republic). Surface area and pore size distribution of samples were analyzed by high throughput surface area and porosity analyzer (Brunauer-Emmett-Teller, BET, Micromeritics ASAP2460, USA), controlled by software MicroActive for ASAP version 2460. The electrode sample size for surface area analysis was 0.25 cm^2 each and the total weight of the sample in the sample holder was 0.25 g.

5. Supporting References

(1) Allahnouri, F.; Farhadi, K.; Imanzadeh, H.; Molaei, R.; Eskandari, H. Synthesis and Introducing Au-Cu Alloy Nanoparticles/Porous Silicon as a Novel Modifier of Screen Printed Carbon Electrode in Simultaneous Electrocatalytic Detection of Codeine and Acetaminophen. *Journal of The Electrochemical Society* **2022**, *169* (1), 016512. DOI: 10.1149/1945-7111/ac4ab2.

(2) Wang, S.; Wu, Y.; Gu, Y.; Li, T.; Luo, H.; Li, L.-H.; Bai, Y.; Li, L.; Liu, L.; Cao, Y.; et al. Wearable Sweatband Sensor Platform Based on Gold Nanodendrite Array as Efficient Solid Contact of Ion-Selective Electrode. *Analytical Chemistry* **2017**, *89* (19), 10224-10231. DOI: 10.1021/acs.analchem.7b01560.

(3) Tashkhourian, J.; Daneshi, M.; Nami-Ana, F.; Behbahani, M.; Bagheri, A. Simultaneous determination of hydroquinone and catechol at gold nanoparticles mesoporous silica modified carbon paste electrode. *Journal of Hazardous Materials* **2016**, *318*, 117-124. DOI: https://doi.org/10.1016/j.jhazmat.2016.06.049.

(4) Lin, F.; Vera Anaya, D.; Gong, S.; Yap, L. W.; Lu, Y.; Yong, Z.; Cheng, W. Gold Nanowire Sponge Electrochemistry for Permeable Wearable Sweat Analysis Comfortably and Wirelessly. *ACS Sensors* **2024**, *9* (10), 5414-5424. DOI: 10.1021/acssensors.4c01635.

(5) Hajilari, F.; Farhadi, K.; Eskandari, H.; Allahnouri, F. Application of Cu/porous silicon nanocomposite screen printed sensor for the determination of formaldehyde. *Electrochimica Acta* **2020**, *355*, 136751. DOI: https://doi.org/10.1016/j.electacta.2020.136751.

(6) Karthika, A.; Ramasamy Raja, V.; Karuppasamy, P.; Suganthi, A.; Rajarajan, M. A novel electrochemical sensor for determination of hydroquinone in water using FeWO4/SnO2 nanocomposite immobilized modified glassy carbon electrode. *Arabian Journal of Chemistry* **2020**, *13* (2), 4065-4081. DOI: https://doi.org/10.1016/j.arabjc.2019.06.008.

(7) Afkhami, A.; Soltani-Felehgari, F.; Madrakian, T. Highly sensitive and selective determination of thiocyanate using gold nanoparticles surface decorated multi-walled carbon nanotubes modified carbon paste electrode. *Sensors and Actuators B: Chemical* **2014**, *196*, 467-474. DOI: https://doi.org/10.1016/j.snb.2014.01.115.

(8) Lu, Z.; Wu, L.; Dai, X.; Wang, Y.; Sun, M.; Zhou, C.; Du, H.; Rao, H. Novel flexible bifunctional amperometric biosensor based on laser engraved porous graphene array electrodes: Highly sensitive electrochemical determination of hydrogen peroxide and glucose. *Journal of Hazardous Materials* **2021**, *402*, 123774. DOI: https://doi.org/10.1016/j.jhazmat.2020.123774.

(9) Chen, J.; Lu, Y.; Huang, L.; Shi, Z.; Zheng, Y.; Song, X.; Wu, C.; Wu, Z. Photo-renewable electrode based on porous carbon-loaded La-TiO2 for detection of catechol and hydroquinone. *Journal of Materials Science: Materials in Electronics* **2021**, *32* (2), 1941-1950. DOI: 10.1007/s10854-020-04962-8.

(10) Xi, X.; Wu, D.; Ji, W.; Zhang, S.; Tang, W.; Su, Y.; Guo, X.; Liu, R. Manipulating the Sensitivity and Selectivity of OECT‐Based Biosensors via the Surface Engineering of Carbon Cloth Gate Electrodes. *Advanced Functional Materials* **2020**, *30* (4), 1905361. DOI: 10.1002/adfm.201905361.

(11) Vreeland, R. F.; Atcherley, C. W.; Russell, W. S.; Xie, J. Y.; Lu, D.; Laude, N. D.; Porreca, F.; Heien, M. L. Biocompatible PEDOT:Nafion Composite Electrode Coatings for Selective Detection of Neurotransmitters in Vivo. *Analytical Chemistry* **2015**, *87* (5), 2600-2607. DOI: 10.1021/ac502165f.

(12) Li, W.; Ding, L.; Wang, Q.; Su, B. Differential pulse voltammetry detection of dopamine and ascorbic acid by permselective silica mesochannels vertically attached to the electrode surface. *Analyst* **2014**, *139* (16), 3926-3931.

(13) Alrashidi, A.; El-Sherif, A. M.; Ahmed, J.; Faisal, M.; Alsaiari, M.; Algethami, J. S.; Moustafa, M. I.; Abahussain, A. A. M.; Harraz, F. A. A Sensitive Hydroquinone Amperometric Sensor Based on a Novel Palladium Nanoparticle/Porous Silicon/Polypyrrole-Carbon Black Nanocomposite. *Biosensors* **2023**, *13* (2), 178. DOI: 10.3390/bios13020178.

(14) Chuenjitt, S.; Kongsuwan, A.; Phua, C. H.; Saichanapan, J.; Soleh, A.; Saisahas, K.; Samoson, K.; Wangchuk, S.; Promsuwan, K.; Limbut, W. A poly(neutral red)/porous graphene modified electrode for a voltammetric hydroquinone sensor. *Electrochimica Acta* **2022**, *434*, 141272. DOI: https://doi.org/10.1016/j.electacta.2022.141272.

(15) Wu, Y.; Zhang, T.; Su, L.; Wu, X. Electrodeposited rGO/AuNP/MnO2 Nanocomposite-Modified Screen-Printed Carbon Electrode for Sensitive Electrochemical Sensing of Arsenic(III) in Water. *Biosensors* **2023**, *13* (5), 563. DOI: 10.3390/bios13050563.

(16) Chang, F.; Wang, H.; He, S.; Gu, Y.; Zhu, W.; Li, T.; Ma, R. Simultaneous determination of hydroquinone and catechol by a reduced graphene oxide–polydopamine–carboxylated multi-walled carbon nanotube nanocomposite. *RSC Advances* **2021**, *11* (51), 31950-31958, 10.1039/D1RA06032E. DOI: 10.1039/D1RA06032E.

(17) Zheng, Y.; Chen, J.; Lu, Y.; Song, X.; Shi, Z. Direct synthesis of highly porous interconnected carbon nanosheets from sodium d-isoascorbic acid for the simultaneous determination of catechol and hydroquinone. *New Journal of Chemistry* **2021**, *45* (3), 1721-1726, 10.1039/D0NJ04479B. DOI: 10.1039/D0NJ04479B.

(18) Su, Y. L.; Tai, C. Y.; Zen, J. M. A Simple Method to Tune Up Screen‐Printed Carbon Electrodes Applicable to the Design of Disposable Electrochemical Sensors. *Electroanalysis* **2013**, *25* (11), 2539-2546. DOI: 10.1002/elan.201300382.

(19) Ma, Y.; Li, J.; Wang, L. Porous carbon derived from ZIF-8 modified molecularly imprinted electrochemical sensor for the detection of tert-butyl hydroquinone (TBHQ) in edible oil. *Food Chemistry* **2021**, *365*, 130462. DOI: https://doi.org/10.1016/j.foodchem.2021.130462.

(20) Hammani, H.; Laghrib, F.; Farahi, A.; Lahrich, S.; El Ouafy, T.; Aboulkas, A.; El Harfi, K.; El Mhammedi, M. A. Preparation of activated carbon from date stones as a catalyst to the reactivity of hydroquinone: Application in skin whitening cosmetics samples. *Journal of Science: Advanced Materials and Devices* **2019**, *4* (3), 451-458. DOI: https://doi.org/10.1016/j.jsamd.2019.07.003.

(21) Velmurugan, M.; Karikalan, N.; Chen, S.-M.; Cheng, Y.-H.; Karuppiah, C. Electrochemical preparation of activated graphene oxide for the simultaneous determination of hydroquinone and catechol. *Journal of Colloid and Interface Science* **2017**, *500*, 54-62. DOI: https://doi.org/10.1016/j.jcis.2017.03.112.

(22) Fan, L.; Li, X.; Kan, X. Disposable graphite paper based sensor for sensitive simultaneous determination of hydroquinone and catechol. *Electrochimica Acta* **2016**, *213*, 504-511. DOI: https://doi.org/10.1016/j.electacta.2016.06.096.

(23) Wu, F.; Zhao, J.; Han, D.; Zhao, S.; Zhu, R.; Cui, G. A three-electrode integrated electrochemical platform based on nanoporous gold for the simultaneous determination of hydroquinone and catechol with high selectivity. *Analyst* **2021**, *146* (1), 232-243, 10.1039/D0AN01746A. DOI: 10.1039/D0AN01746A.

(24) Zhang, Y.; Bai, X. Flexible Microsensor Made of Boron-Doped Graphene Quantum Dots/ZnO Nanorod for Voltammetric Sensing of Hydroquinone. *Journal of The Electrochemical Society* **2020**, *167* (2), 027541. DOI: 10.1149/1945-7111/ab6cf5.

(25) Wang, J.; Yin, H.; Meng, X.; Zhu, J.; Ai, S. Preparation of the mixture of graphene nanosheets and carbon nanospheres with high adsorptivity by electrolyzing graphite rod and its application in hydroquinone detection. *Journal of Electroanalytical Chemistry* **2011**, *662* (2), 317-321. DOI: https://doi.org/10.1016/j.jelechem.2011.08.019.

(26) Gan, T.; Sun, J.; Huang, K.; Song, L.; Li, Y. A graphene oxide–mesoporous MnO2 nanocomposite modified glassy carbon electrode as a novel and efficient voltammetric sensor for simultaneous determination of hydroquinone and catechol. *Sensors and Actuators B: Chemical* **2013**, *177*, 412-418. DOI: https://doi.org/10.1016/j.snb.2012.11.033.

(27) Li, S.-J.; Xing, Y.; Wang, G.-F. A graphene-based electrochemical sensor for sensitive and selective determination of hydroquinone. *Microchimica Acta* **2012**, *176* (1), 163-168. DOI: 10.1007/s00604-011-0709-x.

(28) Cotchim, S.; Promsuwan, K.; Dueramae, M.; Duerama, S.; Dueraning, A.; Thavarungkul, P.; Kanatharana, P.; Limbut, W. Development and Application of an Electrochemical Sensor for Hydroquinone in Pharmaceutical Products. *Journal of The Electrochemical Society* **2020**, *167* (15), 155528. DOI: 10.1149/1945-7111/abd0cd.

(29) Li, Y.; Yang, Q.; Feng, Y.; Ye, B.-C. A robust electrochemical sensor based on N,S-FeNi3/C for simultaneous detection of hydroquinone and arbutin in cosmetics. *Microchimica Acta* **2023**, *190* (4), 150. DOI: 10.1007/s00604-023-05733-y.

(30) Promsuwan, K.; Kaewjunlakan, C.; Saichanapan, J.; Soleh, A.; Saisahas, K.; Thipwimonmas, Y.; Kongkaew, S.; Kanatharana, P.; Thavarungkul, P.; Limbut, W. Poly(phenol red) hierarchical micro-structure interface enhanced electrode kinetics for adsorption and determination of hydroquinone. *Electrochimica Acta* **2021**, *377*, 138072. DOI: https://doi.org/10.1016/j.electacta.2021.138072.

(31) Zhang, H.; Bo, X.; Guo, L. Electrochemical preparation of porous graphene and its electrochemical application in the simultaneous determination of hydroquinone, catechol, and resorcinol. *Sensors and Actuators B: Chemical* **2015**, *220*, 919-926. DOI: https://doi.org/10.1016/j.snb.2015.06.035.

(32) Liu, B.; Guo, H.; Sun, L.; Pan, Z.; Peng, L.; Wang, M.; Wu, N.; Chen, Y.; Wei, X.; Yang, W. Electrochemical sensor based on covalent organic frameworks/MWCNT for simultaneous detection of catechol and hydroquinone. *Colloids and Surfaces A: Physicochemical and Engineering Aspects* **2022**, *639*, 128335. DOI: https://doi.org/10.1016/j.colsurfa.2022.128335.

(33) Xin, Y.; Wang, N.; Wang, C.; Gao, W.; Chen, M.; Liu, N.; Duan, J.; Hou, B. Electrochemical detection of hydroquinone and catechol with covalent organic framework modified carbon paste electrode. *Journal of Electroanalytical Chemistry* **2020**, *877*, 114530. DOI: https://doi.org/10.1016/j.jelechem.2020.114530.

(34) Hu, Y.; Wang, X.; Wang, C.; Hou, P.; Dong, H.; Luo, B.; Li, A. A multifunctional ratiometric electrochemical sensor for combined determination of indole-3-acetic acid and salicylic acid. *RSC Advances* **2020**, *10* (6), 3115-3121, 10.1039/C9RA09951D. DOI: 10.1039/C9RA09951D.

(35) Park, J.; Eun, C. Electrochemical Behavior and Determination of Salicylic Acid at Carbon-fiber Electrodes. *Electrochimica Acta* **2016**, *194*, 346-356. DOI: https://doi.org/10.1016/j.electacta.2016.02.103.

(36) Yang, L.; Chen, D.; Wang, X.; Luo, B.; Wang, C.; Gao, G.; Li, H.; Li, A.; Chen, L. Ratiometric electrochemical sensor for accurate detection of salicylic acid in leaves of living plants. *RSC Advances* **2020**, *10* (64), 38841-38846, 10.1039/D0RA05813K. DOI: 10.1039/D0RA05813K.

(37) Vadivaambigai, A.; Senthilvasan, P. A.; Kothurkar, N.; Rangarajan, M. Graphene-Oxide-Based Electrochemical Sensor for Salicylic Acid. *Nanoscience and Nanotechnology Letters* **2015**, *7* (2), 140-146. DOI: 10.1166/nnl.2015.1909.

(38) Cao, X.; Zhu, X.; He, S.; Xu, X.; Ye, Y. Electro-Oxidation and Simultaneous Determination of Indole-3-Acetic Acid and Salicylic Acid on Graphene Hydrogel Modified Electrode. *Sensors* **2019**, *19* (24), 5483.

(39) Detpisuttitham, W.; Phanthong, C.; Ngamchana, S.; Rijiravanich, P.; Surareungchai, W. Electrochemical Detection of Salicylic Acid in Pickled Fruit/Vegetable and Juice. *Journal of Analysis and Testing* **2020**, *4* (4), 291-297. DOI: 10.1007/s41664-020-00127-y.

(40) Rawlinson, S.; McLister, A.; Kanyong, P.; Davis, J. Rapid determination of salicylic acid at screen printed electrodes. *Microchemical Journal* **2018**, *137*, 71-77. DOI: https://doi.org/10.1016/j.microc.2017.09.019.

(41) Kruanetr, S.; Prabhu, R.; Pollard, P.; Fernandez, C. Pharmaceutical electrochemistry: The electrochemical detection of aspirin utilising screen printed graphene electrodes as sensors platforms. *Surface Engineering and Applied Electrochemistry* **2015**, *51* (3), 283-289. DOI: 10.3103/S1068375515030114.

(42) Lee, J.; Arrigan, D. W. M.; Silvester, D. S. Mechanical polishing as an improved surface treatment for platinum screen-printed electrodes. *Sensing and Bio-Sensing Research* **2016**, *9*, 38-44. DOI: https://doi.org/10.1016/j.sbsr.2016.05.006.

(43) Banks, C. E.; Compton, R. G.; Fisher, A. C.; Henley, I. E. The transport limited currents at insonated electrodes. *Physical Chemistry Chemical Physics* **2004**, *6* (12), 3147-3152, 10.1039/B403751K. DOI: 10.1039/B403751K.

(44) Adane, W. D.; Chandravanshi, B. S.; Tessema, M. Highly sensitive and selective electrochemical sensor for the simultaneous determination of tinidazole and chloramphenicol in food samples (egg, honey and milk). *Sensors and Actuators B: Chemical* **2023**, *390*, 134023.

(45) Randviir, E. P. A cross examination of electron transfer rate constants for carbon screen-printed electrodes using Electrochemical Impedance Spectroscopy and cyclic voltammetry. *Electrochimica Acta* **2018**, *286*, 179-186. DOI: https://doi.org/10.1016/j.electacta.2018.08.021.

(46) Gualandi, I.; Scavetta, E.; Zappoli, S.; Tonelli, D. Electrocatalytic oxidation of salicylic acid by a cobalt hydrotalcite-like compound modified Pt electrode. *Biosensors and Bioelectronics* **2011**, *26* (7), 3200-3206. DOI: https://doi.org/10.1016/j.bios.2010.12.026.

(47) Doulache, M.; Benchettara, A.; Trari, M. Detection of salicylic acid by electrocatalytic oxidation at a nickel-modified glassy carbon electrode. *Journal of Analytical Chemistry* **2014**, *69* (1), 51-56. DOI: 10.1134/S1061934814010067.

(48) Tian, Z.; Tong, X.; Sheng, G.; Shao, Y.; Yu, L.; Tung, V.; Sun, J.; Kaner, R. B.; Liu, Z. Printable magnesium ion quasi-solid-state asymmetric supercapacitors for flexible solar-charging integrated units. *Nature Communications* **2019**, *10* (1), 4913. DOI: 10.1038/s41467-019-12900-4.

(49) Parrilla, M.; Cánovas, R.; Jeerapan, I.; Andrade, F. J.; Wang, J. A Textile-Based Stretchable Multi-Ion Potentiometric Sensor. *Advanced Healthcare Materials* **2016**, *5* (9), 996-1001. DOI: 10.1002/adhm.201600092.

(50) Gupta, V. D. Quantitative Determination of Salicylic Acid and Benzoic Acid in Ointments. *Journal of Pharmaceutical Sciences* **1972**, *61* (10), 1625-1626. DOI: https://doi.org/10.1002/jps.2600611017.
